# Yeast “Make-Accumulate-Consume” Life Strategy Evolved as a Multi-Step Process That Predates the Whole Genome Duplication

**DOI:** 10.1371/journal.pone.0068734

**Published:** 2013-07-15

**Authors:** Arne Hagman, Torbjörn Säll, Concetta Compagno, Jure Piskur

**Affiliations:** 1 Department of Biology, Molecular Cell Biology, Lund University, Lund, Sweden; 2 Department of Food, Environmental and Nutritional Sciences, University of Milan, Milano, Italy; Institut de Genetique et Microbiologie, France

## Abstract

When fruits ripen, microbial communities start a fierce competition for the freely available fruit sugars. Three yeast lineages, including baker’s yeast *Saccharomyces cerevisiae*, have independently developed the metabolic activity to convert simple sugars into ethanol even under fully aerobic conditions. This fermentation capacity, named Crabtree effect, reduces the cell-biomass production but provides in nature a tool to out-compete other microorganisms. Here, we analyzed over forty *Saccharomycetaceae* yeasts, covering over 200 million years of the evolutionary history, for their carbon metabolism. The experiments were done under strictly controlled and uniform conditions, which has not been done before. We show that the origin of Crabtree effect in *Saccharomycetaceae* predates the whole genome duplication and became a settled metabolic trait after the split of the *S. cerevisiae* and *Kluyveromyces* lineages, and coincided with the origin of modern fruit bearing plants. Our results suggest that ethanol fermentation evolved progressively, involving several successive molecular events that have gradually remodeled the yeast carbon metabolism. While some of the final evolutionary events, like gene duplications of glucose transporters and glycolytic enzymes, have been deduced, the earliest molecular events initiating Crabtree effect are still to be determined.

## Introduction

**Figure 1:**
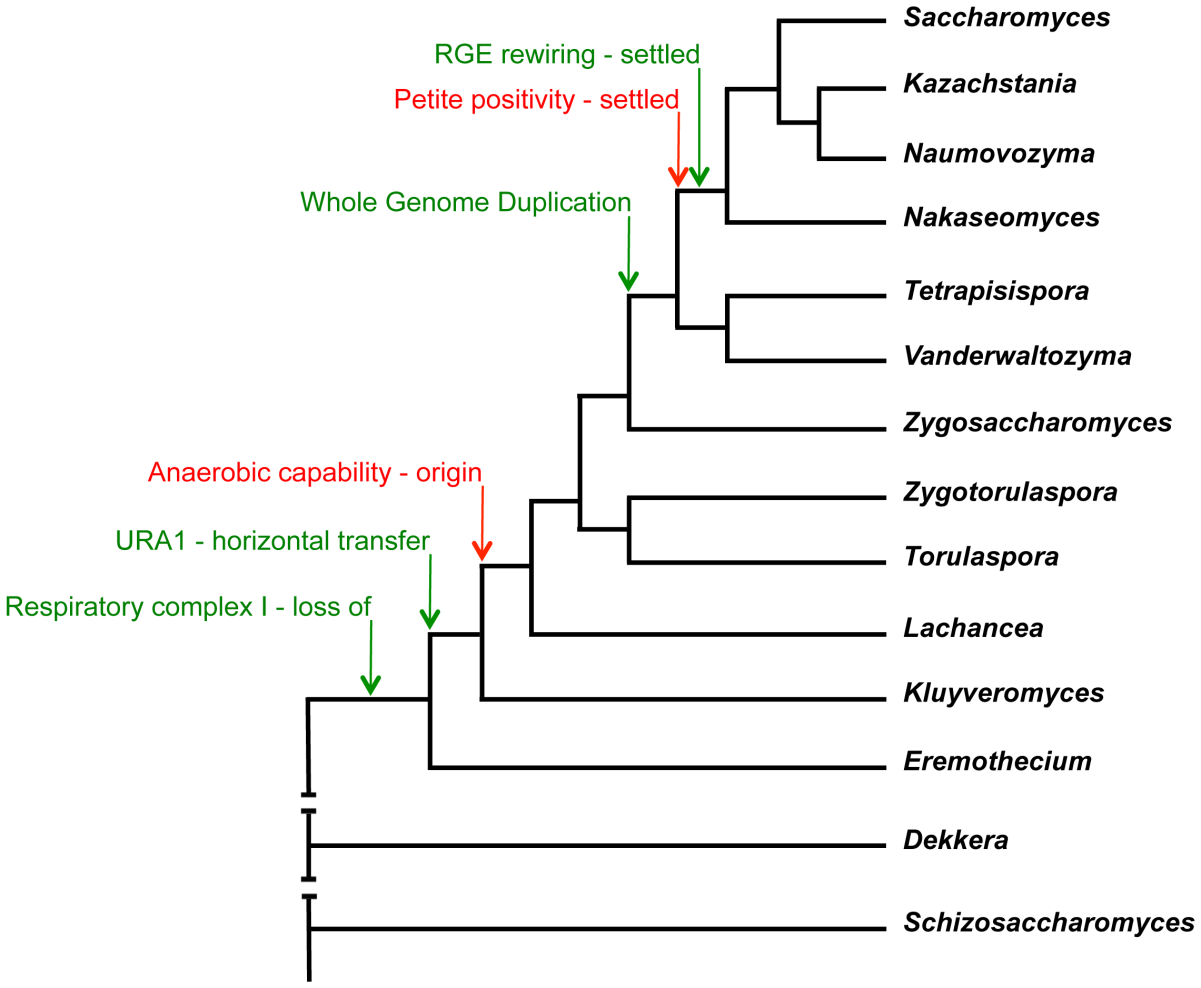
Phylogenetic relationship among yeast. A schematic phylogenetic relationship, based on the phylogenetic tree from Kurtzman and Robnett (2003) [16], covering twelve genera of *Saccharomycetaceae* and all employed species. Note that alternative models to explain the phylogenetic relationship between the *Lachancea*, *Kluyveromyces* and *Eremothecium* genera have been proposed [23] but here we follow the tree in ref. 15. Several evolutionary events, which are relevant for the modern traits, are shown. Note that the relative timing of some events, especially those which left a clear finger-print in the modern genomes (green arrows) is relatively precise, such as WGD [10], the horizontal transfer of a bacterial »anaerobic« DHODase (encoded by *URA1*) [19], complete rewiring of the respiration related promoters (RGE stands for Rapid Growth Elements) [3], and the loss of respiratory Complex I [9], while the timing of more complex traits (red arrows), such as the capability for anaerobic growth [12], [13] and petite positivity [13], might be less precise.

The evolution of plants led to accumulation of larger amounts of mono- and oligo-saccharides, which are now among the favorite substrates for several microbes. Each time when fruits ripen, a fierce competition for the fruit sugars starts, but usually yeasts become the predominant group in these niches. Among the “winners” are usually three yeast lineages, including baker’s yeast *Saccharomyces cerevisiae*, which have independently developed the metabolic activity to convert simple sugars into ethanol even under fully aerobic conditions [1], [2]. During the fermentation the energy for growth is provided by the glycolysis and fermentation pathways, and not by the oxidative respiration pathway. This metabolic activity, called Crabtree effect [3], reduces the production of cell-biomass, but provides a tool, ethanol, to out-compete other microorganisms [1], [4], [5]. Both budding yeast ethanol-producing groups, including *S. cerevisiae* and *Dekkera bruxellensis*, can also efficiently catabolize ethanol and therefore their corresponding lifestyle has been named as “make-accumulate-consume (ethanol)” strategy [1], [4], [5]. On the other hand, the third Crabtree positive group, including the fission yeast *Schizosaccharomyces pombe*, can only poorly metabolize ethanol [6]. Apart from the three mentioned groups, also a few other single lineages, for example *Candida maltosa* exhibit a weak Crabtree effect [7]. These observations could be interpreted as that (i) Crabtree effect originated early in evolution of *Ascomycetes* but has been later lost in several lineages, or (ii) the fermentative life style has appeared and been selected simultaneously in several lineages. The onset of yeast genomics [8] has provided a tool to reconstruct several molecular events that have shaped the budding yeasts during their evolutionary history [9]. Several molecular events have left a clear finger-print in the modern genomes, while the origin of more complex traits, like the fermentation ability, is often not easy to determine using only a genome analysis approach (figure 1). The whole genome duplication (WGD) [10], which took place app. 100 million years ago (mya), and duplication of the alcohol dehydrogenase encoding gene and genes encoding hexose transporters [4], [11] have been proposed as a possible molecular background for development of Crabtree effect and the “make-accumulate-consume” strategy in the *S. cerevisiae* lineage. On the other hand, the ability of *Lachancea* yeasts to grow without oxygen [12], [13] suggests that the “invention” of the ability to grow in the absence of oxygen, took place much earlier, at least 125–150 mya, before the split of the *Saccharomyces* and *Lachancea* lineages.

Here, we studied over forty yeast species, which in nature may occupy similar niches and rely on glucose as the “preferred” substrate [14], and analyzed their carbon metabolism using uniform experimental conditions during the fully controlled growth in fermenters. Our results can be interpreted as that the ability to produce ethanol under aerobic conditions originated before the WGD and evolved progressively.

## Results and Discussion

### Crabtree Positive and Negative Yeasts

The studied species belong to the *Saccharomycotina*, covering over 200 million years of the yeast evolutionary history [15], and including six WGD genera, *Saccharomyces*, *Kazachstania*, *Naumovozyma*, *Nakaseomyces*, *Tetrapisispora* and *Vanderwaltozyma*, and six non-WGD genera, *Zygosaccharomyces*, *Zygotorulaspora*, *Torulaspora*, *Lachancea*, *Kluyveromyces* and *Eremothecium* [16]. The phylogenetic relationship among these yeasts and several molecular events, which has shaped their evolutionary history, and their timing are shown (figure 1). We know that in the modern yeast *Saccharomyces cerevisiae* at least two of these events, WGD and RGE rewiring, contributed to the observed Crabtree effect, but were during evolution completed at two different time points. In addition, also the expansion of hexose transporters plays a role in efficient Crabtree effect, and this gene duplication occurred in several steps [11]. One should note that several alternative phylogenetic trees have been proposed, trying to explain the yeast phylogeny but we followed the one proposed by Kurtzman in ref. 15 (the phylogenetic tree aspect will be discussed further on in the text).

**Figure 2:**
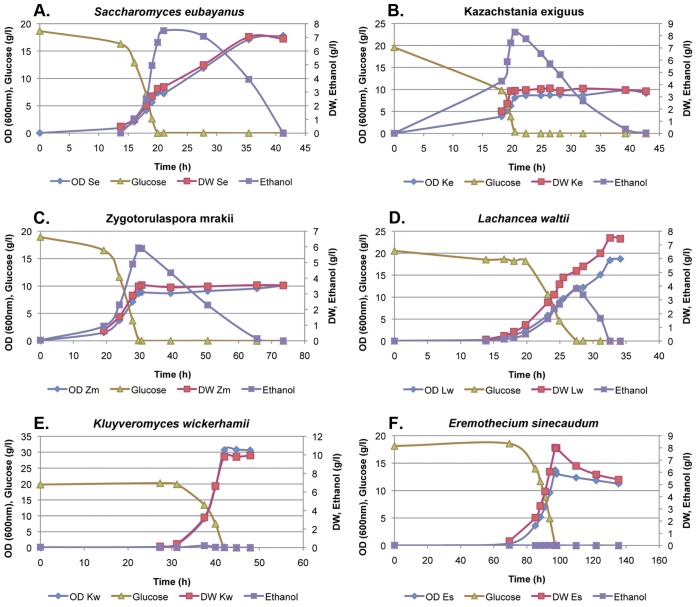
Yeast growth profiles. A few examples of a representative batch culture experiment showing different capacity to produce ethanol and biomass in the presence of excess glucose and oxygen: *Sac. eubayanus* (A), *Kaz. exiguus* (B), *Zto. mrakii* (C), *Lac. waltii* (D), *Klu. wickerhamii* (E) and *Ere. sinecaudum* (F). The graphs show time dependence of yeast glucose consumption, and appearance of the fermentation products and biomass. The ethanol and biomass yields vary enormously among the six shown species, as well as among other studied yeasts (table 1), and are related to the phylogenetic position of each studied yeast species (see also figure 1).

**Figure 3:**
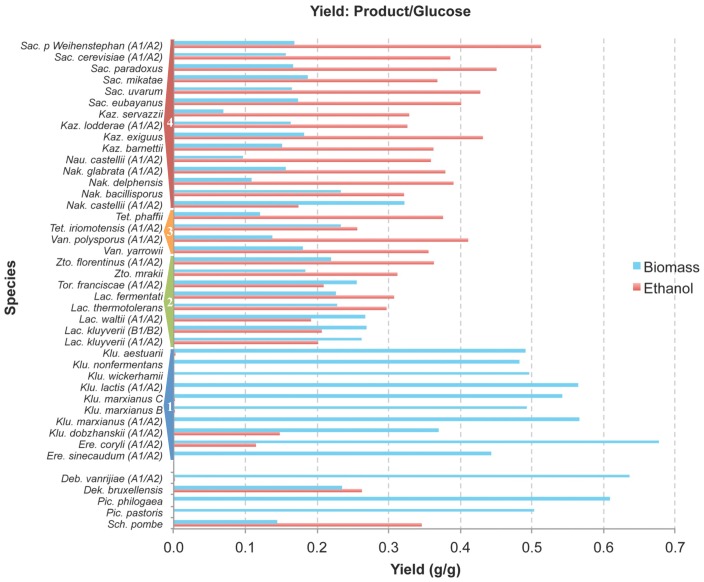
Yeast ethanol and biomass yield. Different yeast species were studied for their carbon metabolism: ethanol yield as g of ethanol per g of glucose (red), and biomass yield as g of biomass per g of glucose (blue). Detailed results and biological replicates are shown in table 1 but hereby either a single measurement or an average of two replicates are presented. The yeast species are presented starting with the *Saccharomyces* genus at the top and then following a decreasing phylogenetic relationship, following figure 1. The species related the least to *S. cerevisiae* are at the bottom, and the gap divides the *Saccharomycotina* and non-*Saccharomycotina* yeasts. The four groups of yeasts (1, 2, 3 and 4) used in the statistical analysis are shown. In general, the ethanol yield gradually drops and the biomass yield gradually increases with the genetic distance from the *Saccharomyces* yeasts (see also figure 5 A and B).

**Figure 4:**
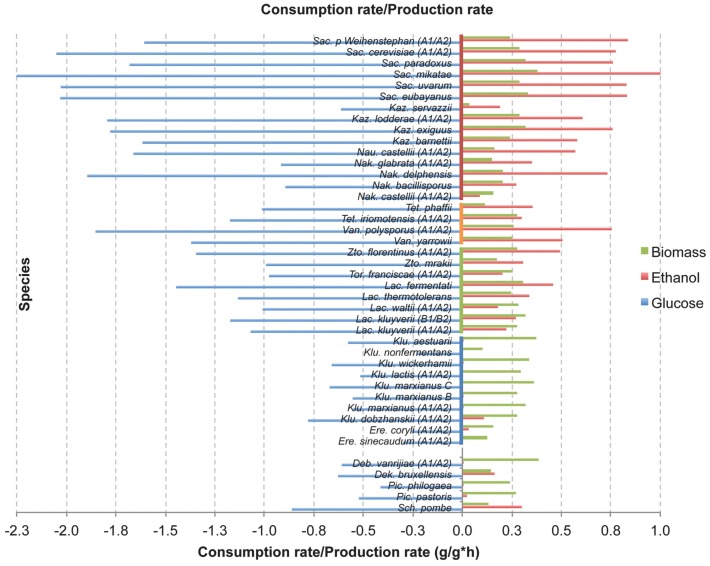
Yeast glucose consumption, ethanol production and growth rates. Different yeast species were studied for their carbon metabolism and the results are shown as: specific glucose consumption rate (g/g h^-1^ amount of consumed glucose by 1 g of biomass and multiplied with the corresponding specific growth rate, shown in blue), specific ethanol production rate (g/g h^-1^ ethanol produced by 1 g biomass and multiplied with the corresponding specific growth rate, shown in red), and specific growth rate (per hour, shown in green). Detailed results and biological replicates are shown in table 1 but hereby either a single measurement or an average of two replicates are presented. The yeast species are presented starting with the *Saccharomyces* genus at the top and then following a decreasing phylogenetic relationship, according to figure 1. The species related the least to *S. cerevisiae* are at the bottom, and the gap divides the *Saccharomycotina* and non-*Saccharomycotina* yeasts. The four groups of yeasts (1, 2, 3 and 4) used in the statistical analysis are shown (see also figure 3). Specific glucose consumption rate decreases with the phylogenetic distance from the *Saccharomyces* genus, indicating that Crabtree negative yeasts have only a moderate rate, while Crabtree positive have faster glucose consumption rate. On the other hand, the growth rate (in green) is very similar among all *Saccharomycotina* yeasts (see also figure 5 C and D).

We analyzed several representatives of each genus to get a coherent picture of their carbon metabolism but we excluded the *Zygosaccharomyces* genus because of its preference for fructose [17] to keep the experimental conditions strictly the same and therefore the results highly comparable (figures 2 and 3). Such a fully controlled experimental approach, covering so many yeast species, has so far not been presented. Crabtree effect results in lower biomass production because a fraction of sugar is converted into ethanol [3]. This means that more glucose needs to be consumed to achieve the same yield of cells and this could theoretically result in lower growth rate in Crabtree positive yeasts. In nature, a lower growth rate could have a negative effect in competition with other microbes. In our experiments *S. cerevisiae* was a reference yeast exhibiting a fully expressed Crabtree effect, with ethanol yield of app. 0.39 g per g of glucose and biomass yield of only 0.16 g per g of glucose while *Kluyveromyces lactis* represented a standard Crabtree negative yeast, which under fully aerobic conditions did not produce any ethanol and its biomass yield was 0.57 g per g glucose. In other words, *S. cerevisiae* used over 6 g glucose to generate 1 g of biomass, and this biomass produced over 2.5 g of ethanol, while in *K. lactis* less than 2 g of glucose was needed for 1 g of biomass (table 1; figures 3 and S1). However, even if they used a different fraction of glucose for the generation of new biomass, the growth rate of both yeasts was very similar, 0.287 for *S. cerevisiae* and 0.298 for *K. lactis* (table 1 and figure 4). Regarding Crabtree positive yeasts, in a majority of strains, including *S. cerevisiae*, the glucose was completely depleted at the point where ethanol concentration reached the maximum and started to be utilized as a carbon source. This gives a very sharp border between the fermentative and respiratory metabolism. In a few yeasts, like *E. coryli* and *Z. rouxii*, the ethanol maximum was reached when some glucose was still present. This means that there was a time period of mixed metabolism, fermentative and respiratory (table S1). Anyhow, we attempted to treat all species and the obtained fermentation results in a comparable way (see also Materials and Methods).

**Table 1:** Central carbon metabolism of characterized yeast species in this study.

Species	Y	CBS	Other	Yield: EtOH/Glc (g/g)	Yield: Biomass/Glc (g/g)	Cons. rate: Glc/Biomass/h (g/gDW,h)	Prod. rate: EtOH/Biomass/h (g/gDW,h)	Growth rate*: (1/h)
*Sac. weihenstephan A2*	Y1288		34/70	0.52	0.17	1.44	0.75	0.232
*Sac. weihenstephan A1*	Y1288		34/70	0.50	0.17	1.62	0.84	0.222
*Sac. cerevisiae A2*	Y706	8340	CEN.PK113-7D	0.38	0.15	2.17	0.81	0.289
*Sac. cerevisiae A1*	Y706	8340	CEN.PK113-7D	0.39	0.16	1.84	0.71	0.271
*Sac. Paradoxus*	Y052	432	NRRLY-17217	0.45	0.17	1.79	0.81	0.338
*Sac. Mikatae*	Y393	8839	IFO1815	0.37	0.19	2.90	1.12	0.374
*Sac. Uvarum*	Y1124		CECT12600	0.43	0.17	1.96	0.80	0.281
*Sac. eubayanus*	Y1693	12357		0.40	0.17	2.00	0.82	0.324
*Kaz. servazzii*	Y055	4311	NRRLY-12661	0.33	0.07	0.62	0.19	0.036
*Kaz. lodderae A2*	Y489	2757	NRRLY-8280	0.31	0.16	1.87	0.62	0.303
*Kaz. lodderae A1*	Y489	2757	NRRLY-8280	0.34	0.16	1.75	0.60	0.281
*Kaz. exiguus*	Y670	1514	NRRLY-1538	0.43	0.43	0.18	1.75	0.740
*Kaz. barnettii*	Y477		NRRLY-27223	0.36	0.15	1.56	0.56	0.234
*Nau. castellii A2*	Y056	4309	NRRLY-27223	0.36	0.10	1.83	0.66	0.187
*Nau. castellii A1*	Y056	4309	NRRLY-27223	0.36	0.09	1.58	0.56	0.147
*Nak. glabrata A2*	Y475	138	NRRLY-1417	0.40	0.15	0.95	0.37	0.152
*Nak. glabrata A1*	Y475	138	NRRLY-1417	0.36	0.16	0.88	0.33	0.145
*Nak. delphensis*	476	2170	NRRLY-2379	0.39	0.11	1.87	0.72	0.202
*Nak. bacillisporus*	Y483	7720	UWOPS85-349.2	0.32	0.23	0.89	0.27	0.203
*Nak. castellii A2*	Y484	4332	NRRLY-17070	0.16	0.34	0.46	0.09	0.140
*Nak. castellii A1*	Y484	4332	NRRLY-17070	0.18	0.31	0.52	0.09	0.159
*Tet. blattae A2*	Y481	6284	NRRLY-10934	0.22	0.16	0.92	0.18	0.140
*Tet. blattae A1*	Y481	6284	NRRLY-10934	0.24	0.17	0.91	0.19	0.141
*Tet. phaffii*	Y482	4417	NRRLY-8282	0.38	0.12	0.89	0.31	0.099
*Tet. iriomotensis A2*	Y1299	8762	IFO10929	0.25	0.23	1.09	0.26	0.265
*Tet. iriomotensis A1*	Y1299	8762	IFO10929	0.26	0.24	1.21	0.33	0.284
*Van. polysporus A2*	Y1293	2163	NRRLY-8283	0.41	0.14	1.90	0.79	0.261
*Van. polysporus A1*	Y1293	2163	NRRLY-8283	0.41	0.13	1.84	0.73	0.258
*Van. yarrowii*	1677		NRRLY-17763	0.36	0.18	1.38	0.51	0.252
*Zsa. rouxii A2*	Y111	732	NRRLY-229	0.04	0.51	0.30	0.01	0.150
*Zsa. rouxii A1*	Y111	732	NRRLY-229	0.04	0.51	0.29	0.01	0.147
*Zsa. bisporus*	Y062	702	NRRLY-12626	0.16	0.31	0.49	0.06	0.153
*Zto. florentinus A2*	Y479	746	NRRLY-1560	0.35	0.21	1.35	0.51	0.276
*Zto. florentinus A1*	Y479	746	NRRLY-1560	0.37	0.22	1.35	0.48	0.275
*Zto. mrakii*	Y480	4218	NRRLY-12654	0.31	0.18	0.97	0.30	0.171
*Tor. franciscae A2*	Y1055	2926	NRRLY-6686	0.22	0.22	0.27	0.95	0.200
*Tor. franciscae A1*	Y1055	2926	NRRLY-6686	0.20	0.20	0.25	1.03	0.210
*Lac. fermentati*	Y083	4506	NRRLY-7434	0.31	0.23	1.41	0.45	0.300
*Lac. thermotolerans*	Y688	6340	NRRLY-8284	0.30	0.23	1.06	0.32	0.229
*Lac. waltii A2*	Y1062	6430	NRRLY-8285	0.19	0.25	0.87	0.15	0.245
*Lac. waltii A1*	Y1062	6430	NRRLY-8285	0.20	0.28	1.14	0.21	0.325
*Lac. kluyverii B2*	Y1651		UWOPS79-150	0.16	0.27	1.15	0.18	0.315
*Lac. kluyverii B1*	Y1651		UWOPS79-150	0.26	0.27	1.18	0.36	0.322
*Lac. kluyverii A2*	Y057	3082	NRRLY-12651	0.20	0.26	1.09	0.24	0.278
*Lac. kluyverii A1*	Y057	3082	NRRLY-12651	0.20	0.26	1.03	0.20	0.271
*Klu. aestuarii*	Y797	4438	NRRLYB-4510	0.00	0.49	0.66	0.00	0.429
*Klu. nonfermentans*	Y1057	8778	JCM10232	0.00	0.48	0.22	0.00	0.101
*Klu. wickerhamii*	Y113	2745	UCD54-210	0.00	0.50	0.63	0.00	0.321
*Klu. lactis A2*	Y707	2359	NRRLY-1140	0.00	0.57	0.48	0.00	0.255
*Klu. lactis A1*	Y707	2359	NRRLY-1140	0.00	0.56	0.57	0.00	0.341
*Klu. marxianus C*	Y1674	397		0.00	0.54	0.67	0.00	0.358
*Klu. marxianus B*	Y1675	2762	NCYC-970	0.00	0.49	0.54	0.00	0.269
*Klu. marxianus A2*	Y1058	712	NRRLY-8281	0.00	0.57	0.55	0.00	0.316
*Klu. marxianus A1*	Y1058	712	NRRLY-8281	0.00	0.56	0.54	0.00	0.314
*Klu. dobzhanskii A2*	Y796	2104	NRRLY-1974	0.13	0.38	0.75	0.09	0.279
*Klu. dobzhanskii A1*	Y796	2104	NRRLY-1974	0.16	0.36	0.80	0.13	0.271
*Ere. coryli A2*	Y999	2608	NRRLY-12970	0.12	0.70	0.25	0.03	0.158
*Ere. coryli A1*	Y999	2608	NRRLY-12970	0.11	0.65	0.25	0.03	0.151
*Ere. sinecaudum A2*	Y1002	8199	NRRLY-17231	0.00	0.44	0.28	0.00	0.117
*Ere. sinecaudum A1*	Y1002	8199	NRRLY-17231	0.00	0.44	0.29	0.00	0.122
*Deb. vanrijiae A2*	Y060	3024	NRRLY-7430	0.00	0.64	0.64	0.00	0.409
*Deb. vanrijiae A1*	Y060	3024	NRRLY-7430	0.00	0.64	0.57	0.00	0.347
*Dek. bruxellensis*	Y881	2796		0.26	0.23	0.64	0.17	0.147
*Pic. philogaea*	Y074	6696	NRRLY-7813	0.00	0.61	0.42	0.00	0.249
*Pic. pastoris*	Y1294			0.00	0.50	0.52	0.02	0.268
*Sch. pombe*	Y709		Eg282	0.35	0.15	0.82	0.28	0.122
*Growth rate determined from DW measurements								

Different yeast species were studied for their carbon metabolism and the results illustrated in figures 3 and 4 are summarized in this table. Yield of products (biomass and ethanol) relative to consumed substrate (glucose) is presented in the unit g/g (gram product per gram substrate) and is calculated by dividing the amount of product (at the maximum of ethanol concentration for Crabtree positive yeasts, or when glucose was depleted for Crabtree negative yeasts) with the amount of substrate consumed. The consumption rates of substrate and production rate of products is presented in the unit g/g,h (gram substrate or product per gram biomass per hour), and is calculated during the exponential phase of growth by dividing the amount of consumed glucose or produced product with the amount of produced biomass and multiplied with the corresponding specific growth rate. Characterized species names and different collections (Y, CBS and other) that provide them are mentioned in separate columns.

### The Origin of Crabtree Effect

The analyzed yeasts converted glucose into biomass, ethanol and CO_2_, and only traceable amounts of other products, such as acetate and glycerol (table S1). When we analyzed how many grams of glucose were necessary to obtain 1 g of biomass, and what the yield of ethanol was (table 1; figures 2 and 3), we could arbitrarily divide the studied species into three different groups: (i) a group which needed over 5.5 g of glucose for 1 g of biomass (or <0.18 g biomass generated from 1 g glucose) and this then produced over 2 g of ethanol (or >0.33 g ethanol generated from 1 g glucose), and included a great majority of WGD yeasts (genera *Saccharomyces*, *Kazachstania*, *Naumovozyma* and *Nakaseomyces* (but not *N. bacillisporus* and *N. castellii*), *Tetrapisispora* (but not *T.* iriomotensis) and *Vanderwaltozyma*); (ii) a group which needed between 3–5 g of glucose for 1 g biomass (or 0.20–0.33 g of biomass from 1 g glucose), and included *Zygotorulaspora*, *Torulaspora*, *Lachancea* yeasts (which are non-WGD) and the lower branches of WGD yeasts (see the *Tetrapisispora* and *Nakaseomyces* strains mentioned above); (iii) a group which converted a majority of glucose into biomass, so that less than 3 g glucose was needed for 1 g of biomass (or >0.33 g of biomass from 1 g glucose) and almost no ethanol or less than 0.15 g from 1 g of glucose was generated. On average, group 1 produced more ethanol per 1 g glucose than group 2. We then performed a statistical analysis of the results shown in figure 3, but for this purpose we build new groups based on their phylogenetic position.

**Figure 5:**
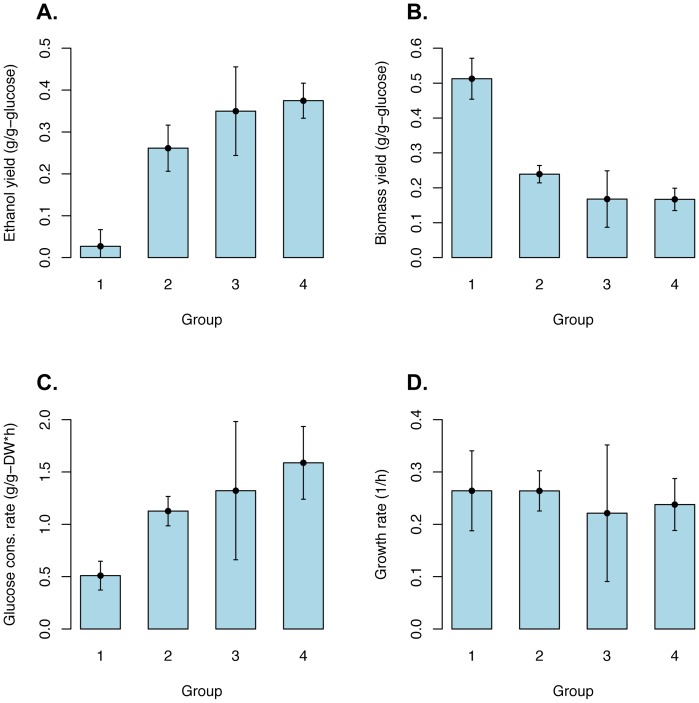
Statistical analysis of the fermentation parameters. Yeasts have been grouped into the following groups: Group 1 included all tested species belonging to *Eremothecium* and *Kluyveromyces*, group 2 all strains of the *Lachancea*, *Torulaspora* and *Zygotorulaspora* genera, group 3 contain the *Vandervaltozyma* and *Tetrapisispora*, and group 4 all species belonging to the *Saccharomyces*, *Kazachstania*, *Naumovozyma* and *Nakaseomyces* genera. The group mean values on four parameters: ethanol yield (A), biomass yield (B), glucose consumption rate (C) and growth rate (D) are illustrated. All error bars in the figure cover a 95% confidence interval for each group. Group 3 appears to be an intermediate between group 2 and 4 for all parameters except growth rate. Hence, no significant difference in growth rates among groups can be observed. These results are also supported by statistical analysis of variance and pairwise t-test (table 2).

The yeast genera were grouped into four groups, based on their phylogenetic relationship and some of the evolutionary steps shown in figure 1, like WGD, the settlement of the RGE rewiring and the origin of anaerobic growth. Group 1 included all tested species belonging to *Eremothecium* and *Kluyveromyces*, group 2 all strains of the *Lachancea*, *Torulaspora* and *Zygotorulaspora* genera, group 3 contained the *Vandervaltozyma* and *Tetrapisispora*, and group 4 all species belonging to the *Saccharomyces*, *Kazachstania*, *Naumovozyma* and *Nakaseomyces* genera. When biomass and ethanol yields were compared for the four groups a large gap, regarding the average values for ethanol yield and biomass yield between group 1 and the rest was found (figures 5 A and B; tables 1 and 2). These differences were highly significant, both tested with regular ANOVA but also when a non-parametric test was used (table 2). Table 2 also shows pairwise t-tests showing significant differences between group 2 and 4, while group 3 overlapped with group 2 as well as 4.

**Table 2:** Statistical test and correlation analysis.

Statistical analysis	Parameter X	Parameter Y	Alpha (5%)	p-value	df	R2	F-val	Correlation
Pearson correlation and Regression	Glucose cons. rate	Biomass yield	Yes	1.42E-06	35.00	0.49	33.64	−0.70
	Glucose cons. rate	Growth rate	Yes	1.98E-02	35.00	0.15	5.96	0.38
	Ethanol yield	Growth rate	Yes	5.72E-01	35.00	0.01	0.33	0.38
Spearman’s rank correlaton rho	Glucose cons. rate	Biomass yield	Yes	2.60E-06	–	–	–	−0.69
	Glucose cons. rate	Growth rate	Yes	3.63E-02	–	–	–	0.35
	Ethanol yield	Growth rate	Yes	3.63E-02	–	–	–	−0.10
ANOVA	Groups	Biomass yield	Yes	1.44E-14	33.00	0.87	72.01	–
		Ethanol yield	Yes	4.90E-13	33.00	0.84	55.94	–
		Glucose cons. rate	Yes	2.00E-05	33.00	0.52	11.88	–
		Growth rate	No	7.60E-01	33.00	0.03	0.40	–
Kruskal-Wallis test	Groups	Biomass yield	Yes	7.14E-06	3.00	–	3.00	–
		Ethanol yield	Yes	6.62E-06	3.00	–	3.00	–
		Glucose cons. rate	Yes	2.13E-04	3.00	–	3.00	–
		Growth rate	No	6.00E-01	3.00	–	3.00	–
Welch Two Sample t-test on Biomass yield	Group 1	Group 2	Yes	5.26E-07	11.82	–	–	–
		Group 3	Yes	4.80E-06	9.18	–	–	–
		Group 4	Yes	7.68E-09	14.93	–	–	–
	Group 2	Group 3	No	5.95E-02	4.07	–	–	–
		Group 4	Yes	7.39E-04	21.00	–	–	–
	Group 3	Group 4	No	9.76E-01	5.30	–	–	–
Welch Two Sample t-test on Ethanol yield	Group 1	Group 2	Yes	1.46E-06	13.79	–	–	–
		Group 3	Yes	4.37E-04	4.79	–	–	–
		Group 4	Yes	3.81E-12	22.69	–	–	–
	Group 2	Group 3	No	7.18E-02	6.04	–	–	–
		Group 4	Yes	1.77E-03	16.28	–	–	–
	Group 3	Group 4	No	5.45E-01	5.29	–	–	–
Welch Two Sample t-test on Glucose consumption rate	Group 1	Group 2	Yes	2.03E-06	15.84	–	–	–
		Group 3	Yes	2.49E-02	3.53	–	–	–
		Group 4	Yes	7.67E-06	17.68	–	–	–
	Group 2	Group 3	No	4.24E-01	3.50	–	–	–
		Group 4	Yes	1.59E-02	17.39	–	–	–
	Group 3	Group 4	No	3.45E-01	7.19	–	–	–
Welch Two Sample t-test on Growth rate	Group 1	Group 2	No	9.97E-01	12.78	–	–	–
		Group 3	No	4.46E-01	7.33	–	–	–
		Group 4	No	5.31E-01	17.02	–	–	–
	Group 2	Group 3	No	3.89E-01	3.98	–	–	–
		Group 4	No	3.67E-01	21.00	–	–	–
	Group 3	Group 4	No	7.39E-01	5.10	–	–	–
Welch Two Sample t-test on Growth rate	*Saccharomyces/Kazachstania*	*Kluyveromyces*	No	5.68E-01	15.12	–	–	–

This table summarizes the results from the statistical analysis conducted on our results. Tests between two parameters (under x- and y-column) are considered to be significant at a significance level (alpha = 5%) or p-value lower than 0.05. ANOVA and Kruskal-Wallis test amongst the four groups reveal significant differences in all parameters (except for growth rate). Hence, there is no significant difference in growth rate among groups 1, 2, 3 and 4. The groups were defined in the main text as well as in figure 5. Pairwise t-tests performed separately on all combinations of groups, on all variables (except growth rate) reveal significant differences between all group combinations (except between groups 2–3, and groups 3–4). Once more, t-test failed to detect any significant differences in growth rates between groups 1, 2, 3 and 4, and the results indicate that group 3 can be seen as an intermediate between group 2 and 4 (see also figure 5). A highly significant correlation between glucose consumption rate and biomass yield is seen on both parametric and non-parametric tests. Thus, our data support a linear model that explains 49% of the variation (See also table S3 for comparison within groups). Furthermore a significant correlation between glucose consumption rate and growth rate, and no significant correlation between growth rate and ethanol yield can be seen with both parametric and non-parametric tests.

In general, the ethanol and biomass yields of each species correspond to its phylogenetic position, or in other words, closely related species exhibit similar traits. This pattern can be interpreted as that a clear Crabtree effect originated just after the split of the *S. cerevisiae* lineage (including all WGD yeasts and *Zygotorulaspora*, *Torulaspora*, *Lachancea* lineages) from the *Kluyveromyces* lineage. However, the observed Crabtree effect was much more pronounced in a majority of WGD yeasts, than in the ethanol producing non-WGD species, suggesting a gradual or at least a two-step “invention”.

Carbon metabolism in the “lower” branches of *Saccharomycetaceae* yeasts, belonging to the modern *Kluyveromyces* and *Eremothecium* species, is similar to other *Saccharomycotina* yeasts, like *Candida albicans*, *Yarrowia lipolytica* and *Pichia pastoris*, which are Crabtree negative yeasts [18] (table 1 and figure 3), confirming that this could be the original property of the *Saccharomycetaceae* yeasts. Therefore, the origin of the “make-accumulate-consume” strategy could take place within the time interval spanning the origin of the ability to grow under anaerobic conditions [12], the *URA1* horizontal transfer [19] and loss of respiratory chain Complex I (figure 1). On the other hand, the second step, leading towards even a more pronounced Crabtree effect, occurred relatively close to the WGD event [10], the settlement of rewiring of the promoters involved in the respiratory part of the carbon metabolism [20], and the settlement of the petite-positive character [13] (figure 1). It appears that *Vanderwaltozyma polyspora* has not yet completely rewired its promoters regarding the RGE-element, and that this process was in yeast first completed after the separation of the *Vanderwaltozyma* and *Saccharomyces* lineages [21]. Thus we consider this evolutionary event to be first completed/settled after the separation of these two lineages (figure 1). Similarly, the petite positive character can be found in all *Saccharomyces*, *Kazachstania*, *Naumovozyma* and *Nakaseomyces* species, while *Tetrapisispora* and *Vanderwaltozyma* contain petite-positive and -negative species [13], thus the trait is first settled after the separation of the *Vanderwaltozyma* and *Saccharomyces* lineages. Apparently, all three characters, the complete promoter rewiring [20], settled petite-positivity [13] and strong Crabtree effect (figure 3), occurred slightly after the WGD event. The first branches after the WGD event, *Tetrapisispora* and *Vanderwaltozyma*, represent a kind of intermediate lineages where the three traits are still in transition.

### Improved Consumption of Glucose

Another interesting aspect is the relationship between Crabtree effect and the yeast growth rate and glucose consumption rate (g glucose/g biomass/time). These parameters were calculated in all species when they grew exponentially on glucose solely (before they started using any accumulated ethanol). In our experiments, Crabtree negative group, *Kluyveromyces* and *Eremothecium*, exhibits a moderate glucose consumption rate, under 0.70 g glucose/g biomass/hour (figure 4). This rate was almost doubled in the *Zygotorulaspora*, *Torulaspora*, *Lachancea* yeasts, and almost tripled, over 1.75 g glucose/g biomass/hour, in some WGD genera, *Saccharomyces* and *Kazachstania*, while *Naumovozyma*, *Nakaseomyces*, *Tetrapisispora* and *Vanderwaltozyma* were rather similar to the “intermediate” group (*Zygotorulaspora*, *Torulaspora* and *Lachancea* yeasts).

The four groups were also compared with respect to, glucose consumption rate and growth rate (figures 5 C and D; tables 1 and 2). Glucose consumption rate showed a similar pattern to ethanol and biomass yield above, *i.e*. there was a large gap, regarding the average values for glucose consumption rate between group 1 and the rest, differences being highly significant. There were also significant differences between group 2 and 4, while group 3 overlapped with group 2 as well as 4. On the other hand, there were no significant differences between the four groups regarding the growth rate (figure 5 D and table 2). When the pronounced Crabtree positive yeasts were considered separately it was found that *Saccharomyces* and *Kazachstania* exhibited a very similar growth rate of approximately 0.25–0.35/h comparing with the clear Crabtree negative yeasts, for example the *Kluyveromyces* species (figure 4), even if these convert a majority of sugars into biomass. In other words, Crabtree positive yeasts exhibited a similar growth rate capacity even if they “wasted” a part of the carbon source for ethanol production (see table 2).

When all species were analyzed there was a clear correlation between biomass yield and glucose consumption rate (figure S4 and table 2). On the other hand, no significant correlation between growth rate and any of the other variables could be found. This is of course in consequence with the absence of significant differences in growth rate between groups. Again, a progressive evolution towards improved consumption can be noticed and it likely occurred in at least two steps. These observations coincide with the gradual gene duplications of hexose transporters [11].

### Evolutionary Timing of Crabtree Effect

Crabtree effect could originate early in the evolution of *Ascomycetes* but has been later lost in several lineages, or the fermentative life style has originated independently in several lineages. The previously obtained results on a very few *Saccharomycetaceae* yeasts could also be interpreted as that Crabtree effect might have originated independently within this monophyletic group [13]. However, in *Saccharomycetaceae* a few independent steps, including WGD, RGE-element rewiring and several independent gene duplications of glucose transporters, which took place at different time points, have been deduced. This step-wise event strategy, apparently strengthened Crabtree effect in the *Saccharomyces* lineage, and suggests that the *Saccharomycetaceae* progenitor was a Crabtree negative yeast. The origin of modern plants with fruits, more than 125 mya [22], brought to microbial communities a new larger and increasingly abundant source of food based on simple sugars. However, ancient yeasts could hardly produce the same amount of new biomass as bacteria during the same time interval. Slower growth rate could in principle be counter-acted by production of a compound, which could inhibit the growth rate of bacteria, like ethanol and acetate. Here we demonstrate that the origin of the Crabtree effect in the *Saccharomyces* lineage took place at an earlier period (figures 1 and 3), 125–150 mya, then suggested before, and it possibly coincided with the independent origin of the Crabtree effect in the *Sch. pombe* lineage and in the *D. bruxellensis* lineage [1], [2] and occurred at the similar time point as the origin of the first modern fruits [22]. While we know the later events, which strengthened the trait, what could be the initial molecular mechanisms, which promoted the evolution of this new lifestyle and rewiring of the carbon metabolism?

### Concluding Remarks

The tremendous change in aerobic/anaerobic properties and carbon metabolism took place just after the split of the *Kluyveromyces* lineage and the lineages leading to *Lachancea*/*Zygotorulaspora*/*Torulaspora*/WGD lineages (table 1; figures 2 and 3). However, this interpretation is based on the phylogenetic relationship [16] that the *Saccharomyces*-*Lachancea* clade is monophyletic regarding *Kluyveromyces* and *Eremothecium*. However, several authors (reviewed in [23]) propose that the correct topology is that *Lachancea*, *Kluyveromyces* and *Eremothecium* form a monophyletic group that is sister to the WGD yeasts. The yeast phylogeny is still controversial but if the later topology is true, it means that the progenitor of all three genera, *Lachancea*, *Kluyveromyces* and *Eremothecium*, was anaerobic and Crabtree positive. This would also claim that the origin of these traits is much older, probably close to the loss of Respiratory complex I. One could then further interpret our results as that the two genera, *Kluyveromyces* and *Eremothecium*, later lost the two traits.

What can one deduce when comparing the published results on physiology of the *Kluyveromyces* and *Lachancea*/WGD species? *S. cerevisiae* and *L. kluyverii* have a Rox1p-mediated system responding to oxygen limiting conditions [24], which regulates gene expression through specific promoter motifs present in hundreds of genes. Similarly, a Mig1p-mediated glucose repression system that is at least partially down-regulating the respiration associated genes, operating in *Saccharomyces* but not in *Kluyveromyces* yeasts, is one of the mechanisms involved in the switch between the fermentative and respiratory mode [25], [26]. The enlargement of a global regulatory system, through “spreading” of the regulatory motifs into new genes, to be controlled for example by Rox1p or Mig1p, could promote colonization of progressively anaerobic niches or promote the ability to more efficiently poison competing bacteria. The *URA1* horizontal transfer [19], [27] and establishment of efficient sterol uptake system [28] also strengthened the Crabtree effect and facultative anaerobiosis phenotypes. Several gene duplications gained during further evolutionary steps increased the carbon flow through glycolysis [29], optimized the conversion between acetaldehyde and ethanol [4], and elevated the sugar uptake. It can be concluded that the ancient environment consisted of lucrative new niches, with an excess of simple sugars originating from the modern plant fruits, which promoted independent evolution of carbon metabolism remodeling in at least three yeast lineages. These could either employ similar or different evolutionary pathways [1] to achieve very similar modern traits.

## Materials and Methods

### Yeast Strains

The yeast species, which were characterized for their respirofermentative properties in this study, belong to the *Ascomycota* phylum, sub-phylum *Saccharomycotina*, clade *Saccharomycetaceae*. The species are presented according to the phylogenetic analysis presented by Kurtzman [16]: *Saccharomyces pastorianus Weihenstephan* 34/70, *Saccharomyces cerevisiae* CBS 8340 (CEN.PK 113-7D), *Saccharomyces paradoxus* CBS 432 (NRRL Y-17217), *Saccharomyces mikatae* CBS 8839, *Saccharomyces uvarum* CECT12600, *Saccharomyces eubayanus* CBS 12357, *Saccharomyces servazzii* CBS 4311 (NRRL Y-12661), *Kazachstania lodderae* CBS 2757 (NRRL Y-8280), *Kazachstania exiguus* CBS 1514, *Kazachstania barnettii* (NRRL Y-27223), *Naumovozyma castellii* CBS 4309 (NRRL Y-12630), *Nakaseomyces glabrata* CBS 138 (NRRL Y-1417), *Nakaseomyces delphensis* CBS 2170 (NRRL Y-2379), *Nakaseomyces bacillisporus* CBS 7720 (NRRL Y-17846), *Nakaseomyces castellii* CBS 4332 (NRRL Y-17070), *Tetrapisispora blattae* CBS 6284 (NRRL Y-10934), *Tetrapisispora phaffii* CBS 4417 (NRRL Y-8282), *Tetrapisispora iriomotensis* CBS 8762 (IFO 10929), *Vanderwaltozyma polysporus* CBS 2163 (NRRL Y-8283), *Vanderwaltozyma yarrowii* (NRRL Y-17763), Zygosaccharomyces bisporus CBS 702 (NRRL Y-12626), Zygosaccharomyces rouxii CBS 732 (NRRL Y-229), *Zygotorulaspora florentinus* CBS 746 (NRRL Y-1560), *Zygotorulaspora mrakii* CBS 4218 (NRRL Y-12654), *Torulaspora franciscae* CBS 2926 (NRRL Y-6686), *Lachancea fermentati* CBS 4506 (NRRL Y-7434), *Lachancea thermotolerans* CBS 6340 (NRRL Y-8284), *Lachancea waltii* CBS 6430 (NRRL Y-8285), *Lachancea kluyverii* UWOPS79-150, *Lachancea kluyvery* CBS 3082 (NRRL Y-12651), *Kluyveromyces aestuarii* CBS 4438 (NRRL YB-4510), *Kluyveromyces nonfermentans* CBS 8778 (NRRL Y-27343), *Kluyveromyces wickerhamii* CBS 2745 (NRRL Y-8286), *Kluyveromyces lactis* CBS 2359 (NRRL Y-1140), *Kluyveromyces marxianus* CBS 712 (NRRL Y-8281), *Kluyveromyces marxianus* CBS 397, *Kluyveromyces marxianus* CBS 2762, *Kluyveromyces dobzhanskii* CBS 2104 (NRRL Y-1974), *Eremothecium coryli* CBS 2608 (NRRL Y-12970), *Eremothecium sinecaudum* CBS 8199 (NRRL Y-17231).

In addition, some species belonging to other *Ascomycota* clades were studied: *Debaromyces vanrijiae* CBS 3024 (NRRL Y-7430), *Dekkera bruxellensis* CBS 2796, *Pichia philogaea* CBS 6696 (NRRL Y-7813), *Pichia pastoris* Y1294, *Schizosaccharomyces pombe* Eg282 and represent a control group. All batch cultivations were verified by sequencing of the inoculum and the final culture (table S1).

Regarding *Zygosaccharomyces bisporus* CBS 702 (NRRL Y-12626) and *Zygosaccharomyces rouxii* CBS 732 (NRRL Y-229), this genus is fructophilic [17] and was therefore not included in the comparisons shown in figures 3 and 4. Moreover, *Tetrapisispora blattae* CBS 6284 (NRRL Y-10934) was isolated from the gut of *Blatta orientalis* (cockroach) and was therefore also excluded from the comparisons in figures 3 and 4. However, the results on these three strains can be found in supplementary information.

### Sequencing

All batch cultivations were verified by sequencing of the rDNA R26 region, amplified by NL-1 (5′-GCATATCAATAAGCGGAGGAAAAG-3′) and NL-4 (5′-GGTCCGTGTTTCAAGACGG-3′) primers. Samples from the end of each batch-cultivation were streaked out on YPD-agar plates (containing 2% D-Glucose) and incubated at 25°C for 2–3 days. Template for colony PCR was prepared by picking small amounts of cells from a single colony with a sterile pipette tip. Cells were resuspended in 20 µl of distilled water and 1 µl of the suspension was used in PCR reactions containing: 1.25 U Taq DNA polymerase (Fermentas), 1X Taq buffer with (NH_4_)_2_SO_4_ (Fermentas), 2.5 mM MgCl_2_ (Fermentas), 2 mM dNTP mix (Promega), 0.4 µM of each primer (Eurofins MWG operon) and sterile MQ-H_2_O were added to a final volume of 50 µl. The PCR-reactions were run with the program: 94°C –4 min, 36 * (94°C –30 sec, 52°C –30 sec, 72°C –1 min), 72°C –7 min, 4°C ∞. PCR-product quality was verified with gel electrophoresis; 1% agarose gel and 1X TBE buffer as a mobile phase. Nanodrop was used for quantification and Eurofins MWG Operon provided the sequencing service. A normal nucleotide blast (Blastn) of NL-1 derived sequence was performed to finalize the verification of species used in the experiments.

### Batch Cultivations

Aerobic batch cultivations were performed in Multifors (INFORS HT) bioreactors with a working volume of either 0.5 or 1 liter. A majority of all batch cultivations were conducted in duplicate at 25°C, with airflow of 1 vvm. The stirrer speed was on cascade mode, automatically varying from 200 to 1200 rpm to maintain a dissolved oxygen concentration above 30%, which was monitored with an InPro 6800S sensor (Mettler Toledo). The pH was maintained at 5 (±0.5 units) by KOH (2 M) and H_2_SO_4_ (1 M), monitored with a 405-DPAS-SC-K8S/225 (Mettler Toledo) sensor. For the calculation of CO_2_ production and O_2_ consumption, which were used to estimate respiration contra fermentation and Carbon-balancing calculations, gas analyzers BC-CO_2_ and BCP-O_2_ (BlueSens) were used to determine the CO_2_ and O_2_ levels in the gas outflow. All overnight pre-inoculum were cultivated and washed before inoculation in defined synthetic minimal medium for aerobic conditions, used in the bioreactors and prepared as reported [30]. Pre-inoculums were approx. 500-fold diluted at the inoculation step, resulting in approximately 500 times higher biomass concentration in the bioreactors, in the end of each experiment. Furthermore, 2.002 g/l Kaiser Synthetic Complete supplement had to be added to batch cultivations of *Eremothecium coryli,* and 150 mg/l histidine to batch cultivations of *Tetrapisispora blattae* due to the auxotrophic nature of the species. In all experiments the only carbon source to be utilized by the studied species was 2% glucose.

### Yeast Central Carbon Metabolism

The determination of glucose, ethanol, acetate and glycerol in the supernatant were conducted with a HPLC 1200 series (Agilent) equipped with a 300*7.7 mm Aminex HPX-87H Column (BioRad). The mobile phase used was 5 mM H_2_SO_4_ set to a flow rate of 0.6 ml/min through a column temperature of 60°C and a RID temperature of 55°C. Growth kinetics was monitored by two methodologies, dry weight (DW) and optical density (OD_600_) measurements (tables 1 and S1). The maximum growth rates were calculated in the exponential growth phase on glucose solely, before the consumption of any accumulated ethanol (figure S1). For the determination of DW, glass microfiber filters GF/A (Whatman) were weighted before and after filtering of the washed culture samples (these were dried at 70°C for 1 day).

Yields of products (biomass, ethanol, acetate and glycerol) relative to consumed substrate (glucose) are presented in the unit g/g and were calculated by dividing the amount of product (at the maximum of ethanol concentration for Crabtree positive yeast, or when glucose was depleted for Crabtree negative yeast) with the amount of substrate consumed (tables 1 and S1).

The consumption rates of substrate (glucose) and production rate of products (biomass, ethanol, acetate and glycerol) are presented in the unit g/gDW,h (gram substrate or product per gram biomass per hour). These rates were calculated between the time points (the same as for the determination of the maximum growth rates) that span the exponential growth phase on glucose solely (figure S1), by dividing the amount of consumed glucose or formed product with the amount of produced biomass, and multiplied with the corresponding specific growth rate. Thus, by dividing the yields of products relative to the consumed glucose with the yield of biomass relative to the consumed glucose (table S2) and multiply with the corresponding specific growth rate (table 1), the consumption and production rates were obtained.

The respiration ratios were calculated by dividing the total amount of CO_2_ produced with total amount O_2_ consumed, during growth on glucose solely, in the unit mole/mole (figure S2). To verify the quality of each experiment, carbon balance was calculated by taking the ratio of formed products in C-mole and consumed substrate in C-mole (figure S3).

### Statistical Analysis

Statistical tests and correlation analysis were performed in R (2.15.2) and summarized in table 2 and S3. Analysis of variance (ANOVA) and non-parametric (Kruskal-Wallis) tests were applied on four parameters (biomass yield, ethanol yield, glucose consumption rate and growth rate) to assess any significant difference among groups (shown in figures 3, 4 and 5). Subsequent pairwise t-tests (Welch two sample t-test) on the group-means of the same four parameters (mentioned above) were then performed between all possible combinations of groups. More specific information on the statistical analysis can be found in Supplementary Material S1.

## Supporting Information

Figure S1 Yeast growth profiles. All characterized yeast species and their growth profiles are shown, both in natural and logarithmic scale. Specific rates were determined from dry weight (DW) and optical density (OD_600_) (illustrated in figure 4, and summarized in table 1 and S1). Substrate (glucose) and metabolite (pyruvate, succinate, lactate, glycerol, acetate and ethanol) concentrations were monitored during growth and were used for yield, production/consumption rates calculation to quantify the Crabtree effect for each species (illustrated in figures 3, 4, S2 and summarized in tables 1 and S1).
(PDF)

Figure S2 Respiration ratio. The respiration ratios for different species illustrates the activity of alcohol fermentation pathway as compared to respiratory pathway and was calculated by dividing the total amount of CO_2_ produced (blue bar) with total amount O_2_ consumed (red bar) in the unit mole/mole (see also table S1). The yeast species are presented starting with the *Saccharomyces* genus at the top and then following a decreasing phylogenetic relationship, following figure 1. The species related the least to *Saccharomyces cerevisiae* are at the bottom, and the gap divides the *Saccharomycotina* and non-*Saccharomycotina* yeasts. In general, the respiration ratio gradually drops with the phylogenetic distance from the *Saccharomyces* yeasts.
(PDF)

Figure S3 Carbon balance. To verify the quality of each experiment, carbon balance was calculated by taking the yield ratio between measured products in C-mole and consumed substrate in C-mole, see also tables 1 and S1 for data on products yield and substrate consumption.
(PDF)

Figure S4 Correlation between biomass yield and glucose consumption rate**.** A significant correlation can be observed between the determined biomass yield and glucose consumption rate for each species (table 1). The rate-yield trade-off is a known phenomenon, which has been observed previously and hypothesized to act as an evolutionary constraint [31].
(PDF)

Table S1 Central carbon metabolism of characterized yeast species in this study. Different yeast species were studied for their carbon metabolism and the results for all analyzed metabolites, not illustrated in figures 3 and 4, are summarized in this table: Yield of products (glycerol, acetate, pyruvate, succinate and lactate) relative to consumed substrate (glucose) is presented in the unit g/g (gram product per gram substrate) and is calculated by dividing the amount of product (at the maximum of ethanol concentration for Crabtree positive yeasts, or when glucose was depleted for Crabtree negative yeasts) with the amount of substrate consumed. Yield of CO_2_ and consumed O_2_ relative to consumed substrate is presented in the unit mole/mole (mole CO_2_ or O_2_ per mole glucose) and is determined in the same time interval as for other yield calculations. Characterized species names, accession number to their best blast hit and Y-collection numbers are mentioned in separate columns.
(XLSX)

Table S2 Central carbon metabolism under exponential growth phase on glucose solely. Yields of all determined products (formed metabolites) relative to consumed substrate (glucose) are presented in the unit g/g (gram product per gram substrate) and is calculated by dividing the amount of product formed during the exponential growth phase on glucose solely, before the consumption of any accumulated ethanol. This may not correspond to the maximum of ethanol concentration for Crabtree positive yeasts, or when glucose was completely depleted for Crabtree negative yeasts (as compared to the yield data summarized in tables 1 and S1).
(XLSX)

Table S3 Correlation between glucose consumption rate and biomass yield within groups. No significant correlationx between growth rate and ethanol yield can be observed within phylogenetic groups on both parametric and non-parametric tests.
(XLSX)

Supplementary Material S1 R-script.
(ZIP)
