# Supplementary material for: Yeast “Make-Accumulate-Consume” Life Strategy Evolved as a Multi-Step Process That Predates the Whole Genome Duplication
Source: PLoS One. 2013 Jul 15;8(7):e68734. doi: 10.1371/journal.pone.0068734 (PMC3711898; doi:10.1371/journal.pone.0068734)
Supplement: Figure S1 — Yeast growth profiles. All characterized yeast species and their growth profiles are shown, both in natural and logarithmic scale. Specific rates were determined from dry weight (DW) and optical density (OD600) (illustrated in figure 4, and summarized in table 1 and S1). Substrate (glucose) and metabolite (pyruvate, succinate, lactate, glycerol, acetate and ethanol) concentrations were monitored during growth and were used for yield, production/consumption rates calculation to quantify the Crabtree effect for each species (illustrated in figures 3, 4, S2 and summarized in tables 1 and S1). (PDF) [file pone.0068734.s001.pdf]

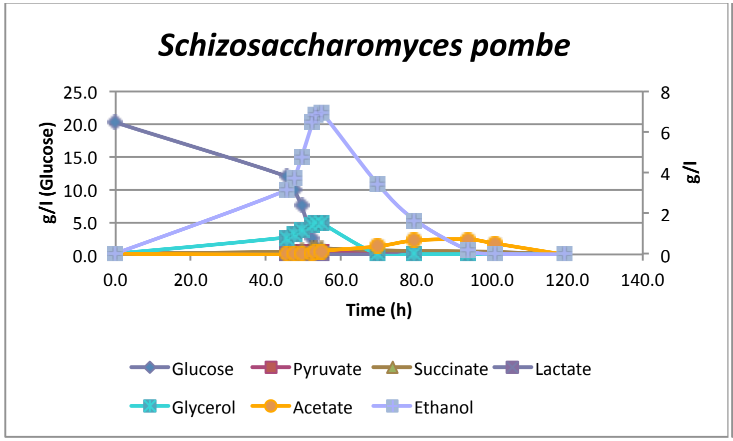
Schizosaccharomyces pombe
g/l (Glucose)
g/l
Time (h)
Glucose
Pyruvate
Succinate
Lactate
Glycerol
Acetate
Ethanol

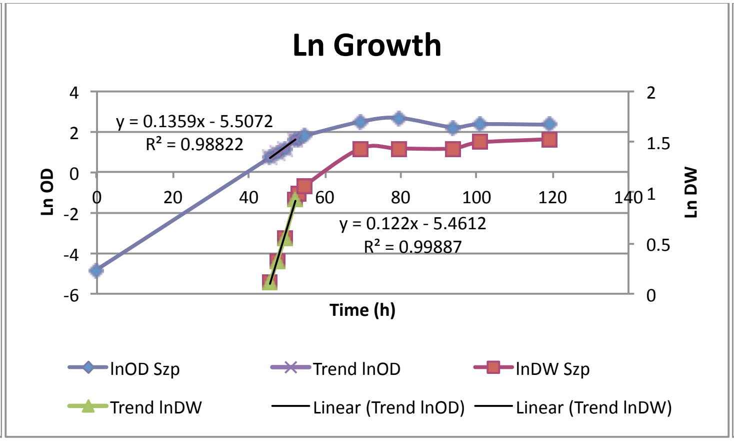
Ln Growth
y = 0.1359x - 5.5072
R² = 0.98822
y = 0.122x - 5.4612
R² = 0.99887
Ln OD
Ln DW
Time (h)
lnOD Szp
Trend lnOD
lnDW Szp
Trend lnDW
Linear (Trend lnOD)
Linear (Trend lnDW)

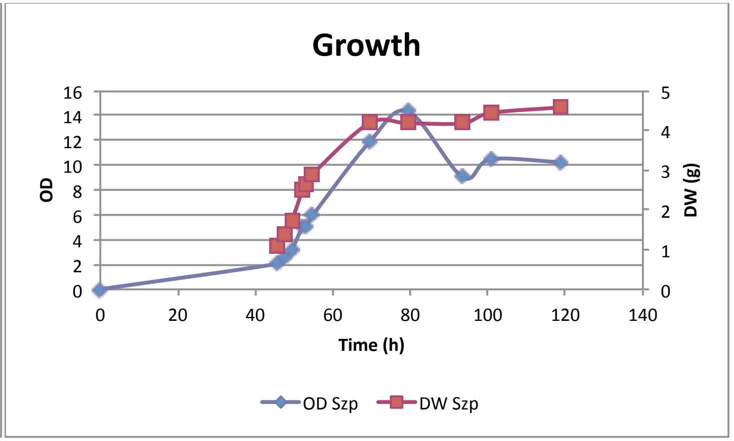
Growth
OD
DW (g)
Time (h)
OD Szp
DW Szp

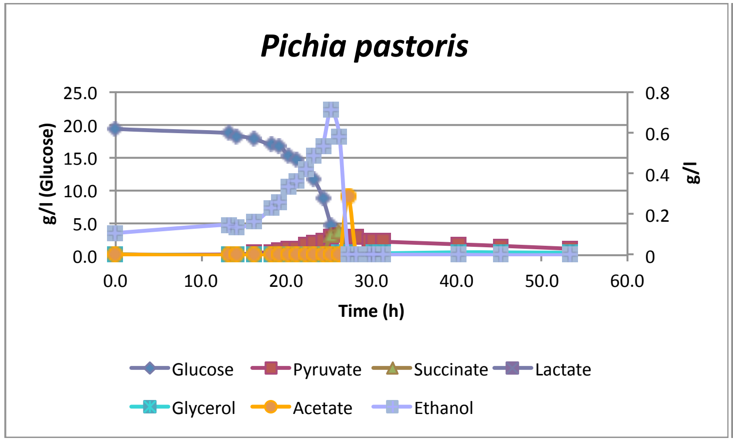
Pichia pastoris
g/l (Glucose)
g/l
Time (h)
Glucose
Pyruvate
Succinate
Lactate
Glycerol
Acetate
Ethanol

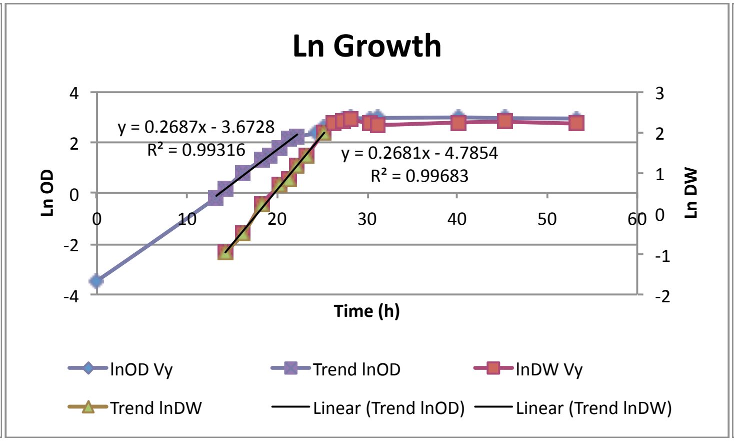
Ln Growth
y = 0.2687x - 3.6728
R² = 0.99316
y = 0.2681x - 4.7854
R² = 0.99683
Ln OD
Ln DW
Time (h)
lnOD Vy
Trend lnOD
lnDW Vy
Trend lnDW
Linear (Trend lnOD)
Linear (Trend lnDW)

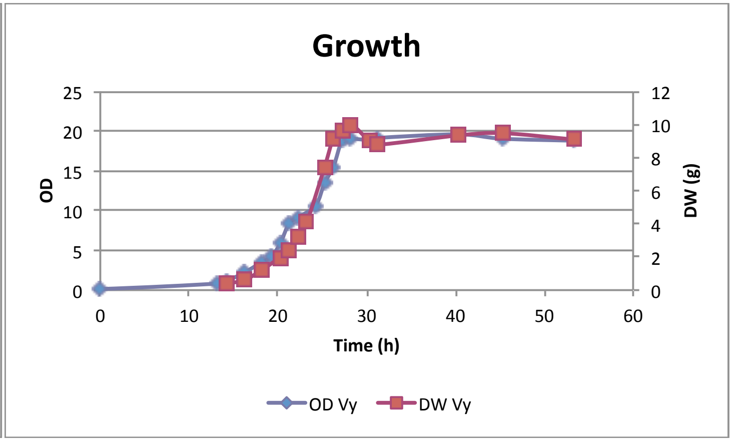
Growth
OD
DW (g)
Time (h)
OD Vy
DW Vy

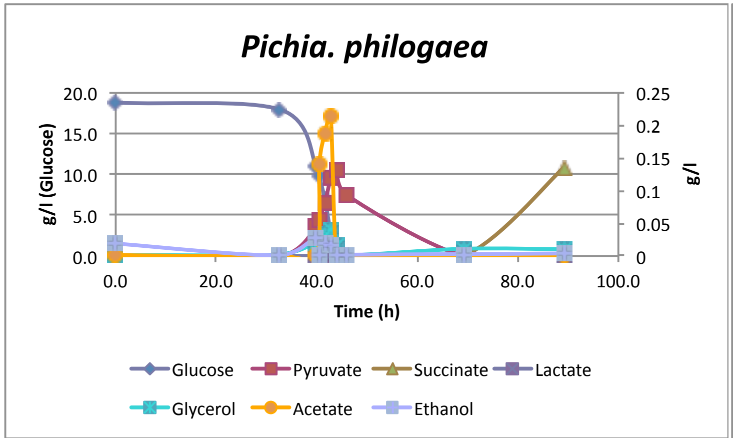
Pichia. philogaea
g/l (Glucose)
g/l
Time (h)
Glucose
Pyruvate
Succinate
Lactate
Glycerol
Acetate
Ethanol

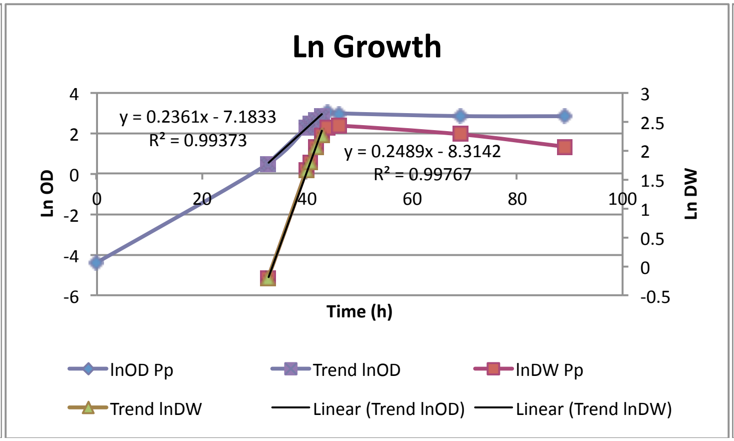
Ln Growth
y = 0.2361x - 7.1833
R² = 0.99373
y = 0.2489x - 8.3142
R² = 0.99767
Ln OD
Ln DW
Time (h)
lnOD Pp
Trend lnOD
lnDW Pp
Trend lnDW
Linear (Trend lnOD)
Linear (Trend lnDW)

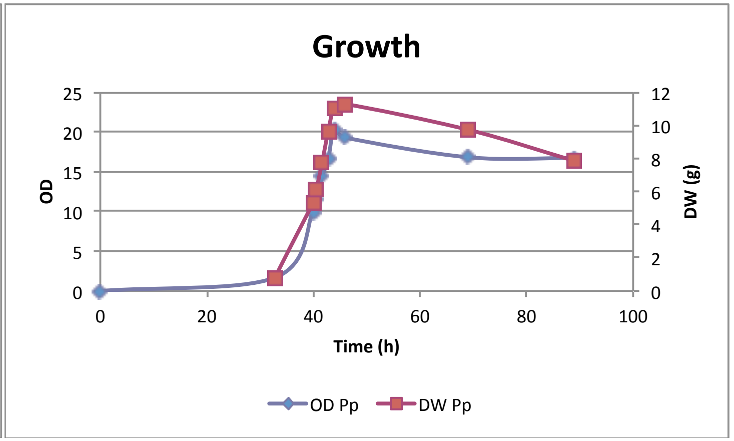
Growth
OD
DW (g)
Time (h)
OD Pp
DW Pp

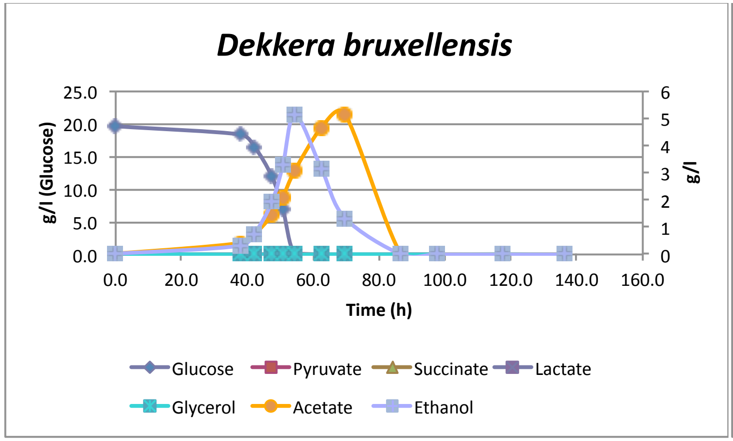

Dekkera bruxellensis
g/l (Glucose)
g/l
Time (h)
Glucose
Pyruvate
Succinate
Lactate
Glycerol
Acetate
Ethanol

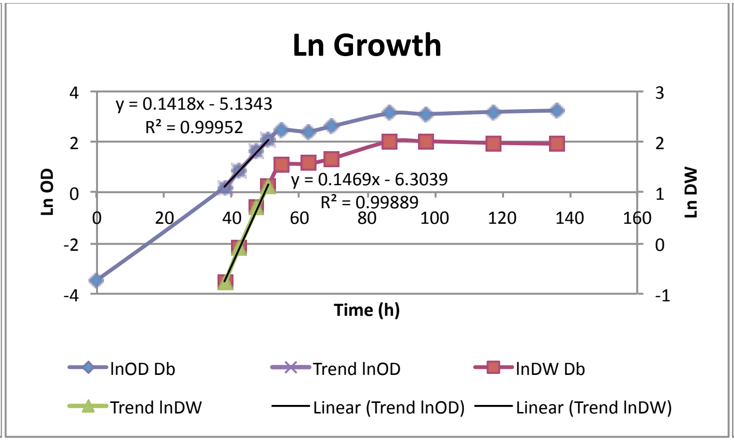

Ln Growth
y = 0.1418x - 5.1343
R² = 0.99952
y = 0.1469x - 6.3039
R² = 0.99889
Ln OD
Ln DW
Time (h)
lnOD Db
Trend lnOD
lnDW Db
Trend lnDW
Linear (Trend lnOD)
Linear (Trend lnDW)

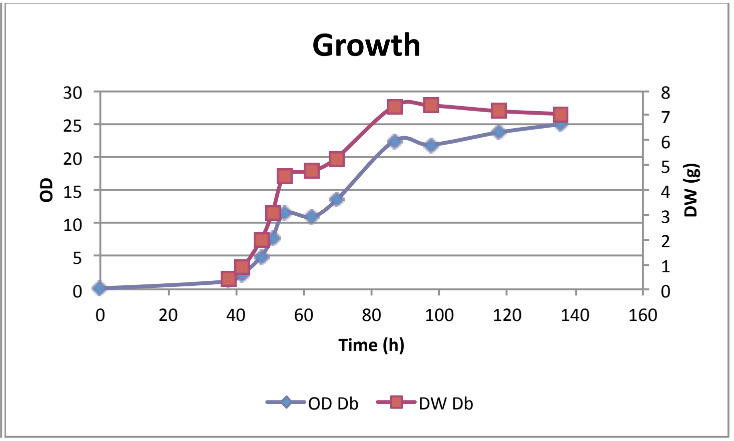

Growth
OD
DW (g)
Time (h)
OD Db
DW Db

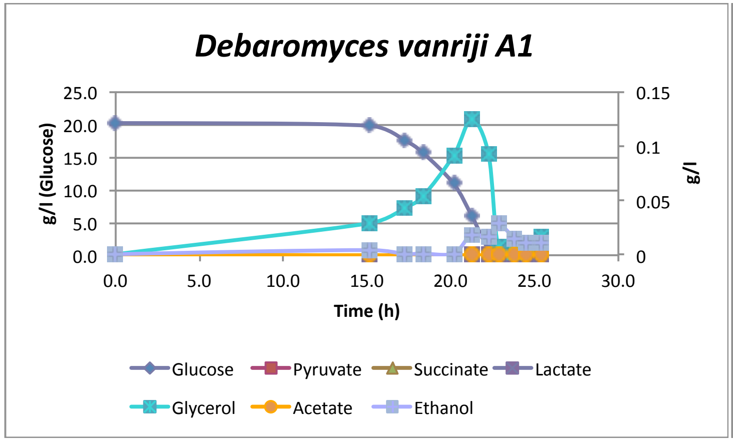

Debaromyces vanriji A1
g/l (Glucose)
g/l
Time (h)
Glucose
Pyruvate
Succinate
Lactate
Glycerol
Acetate
Ethanol

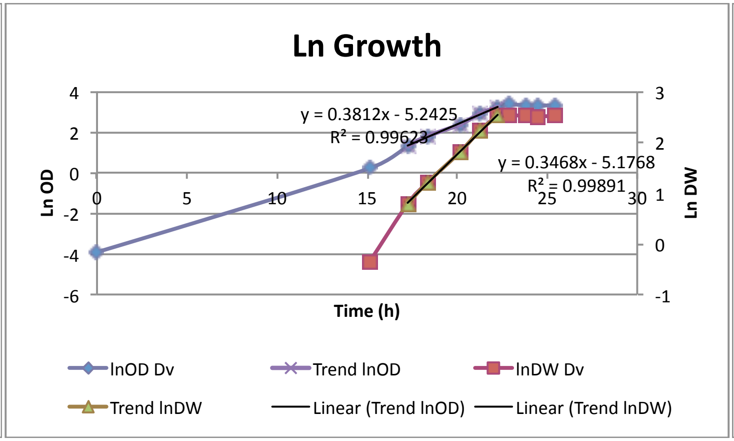

Ln Growth
y = 0.3812x - 5.2425
R² = 0.99623
y = 0.3468x - 5.1768
R² = 0.99891
Ln OD
Ln DW
Time (h)
lnOD Dv
Trend lnOD
lnDW Dv
Trend lnDW
Linear (Trend lnOD)
Linear (Trend lnDW)

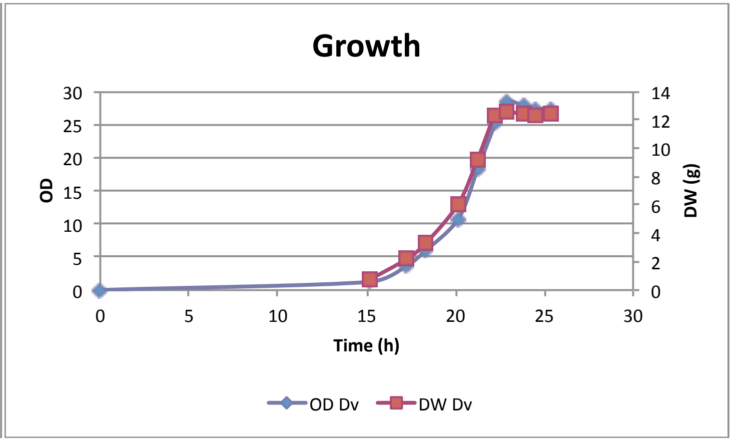

Growth
OD
DW (g)
Time (h)
OD Dv
DW Dv

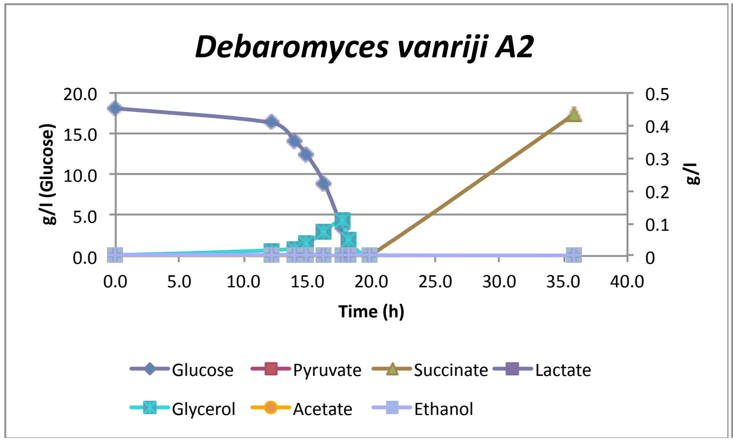

Debaromyces vanriji A2
g/l (Glucose)
g/l
Time (h)
Glucose
Pyruvate
Succinate
Lactate
Glycerol
Acetate
Ethanol

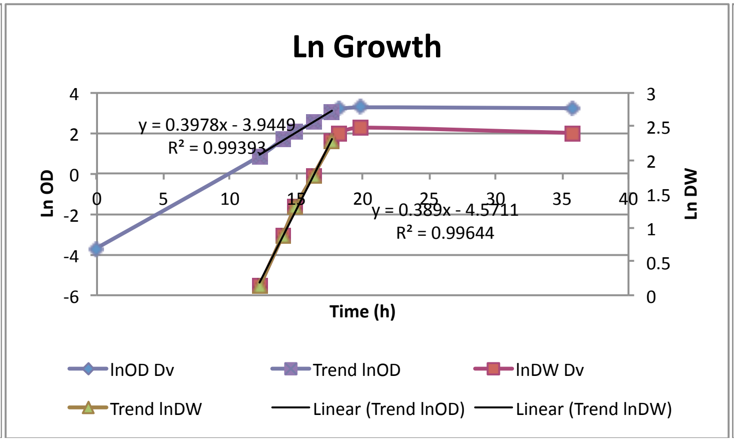

Ln Growth
y = 0.3978x - 3.9449
R² = 0.99393
y = 0.389x - 4.5711
R² = 0.99644
Ln OD
Ln DW
Time (h)
lnOD Dv
Trend lnOD
lnDW Dv
Trend lnDW
Linear (Trend lnOD)
Linear (Trend lnDW)

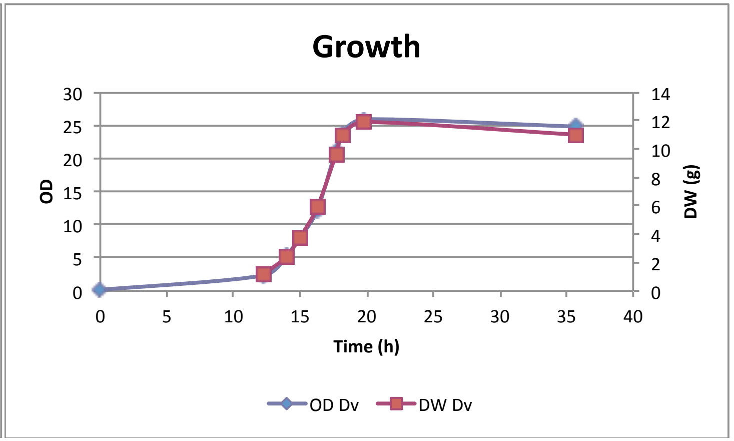

Growth
OD
DW (g)
Time (h)
OD Dv
DW Dv

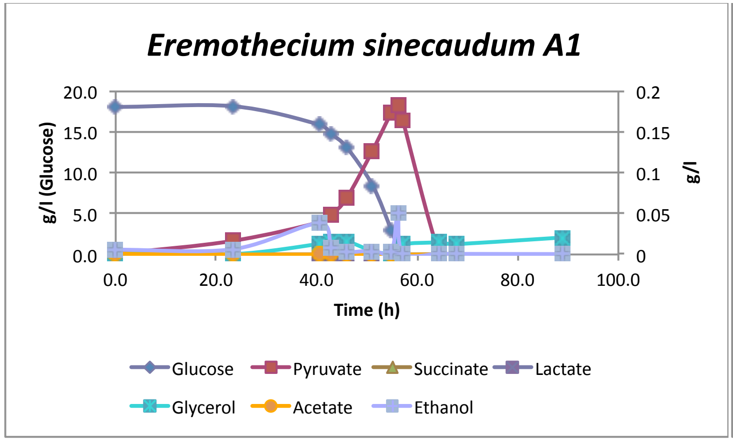
Eremothecium sinecaudum A1
g/l (Glucose)
g/l
Time (h)
Glucose
Pyruvate
Succinate
Lactate
Glycerol
Acetate
Ethanol

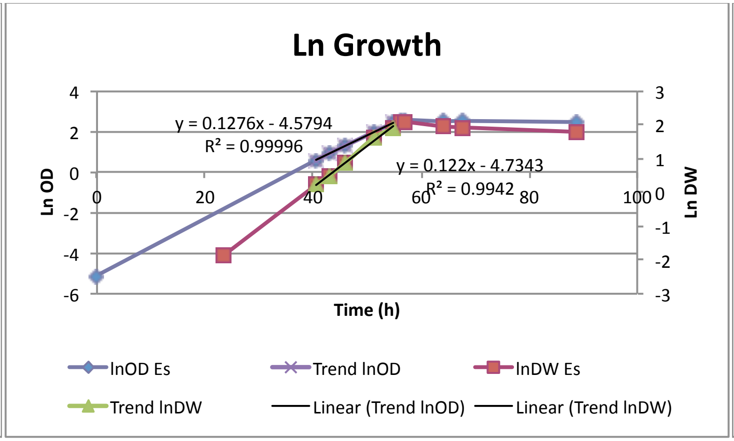
Ln Growth
y = 0.1276x - 4.5794
R² = 0.99996
y = 0.122x - 4.7343
R² = 0.9942
Ln OD
Ln DW
Time (h)
lnOD Es
Trend lnOD
lnDW Es
Trend lnDW
Linear (Trend lnOD)
Linear (Trend lnDW)

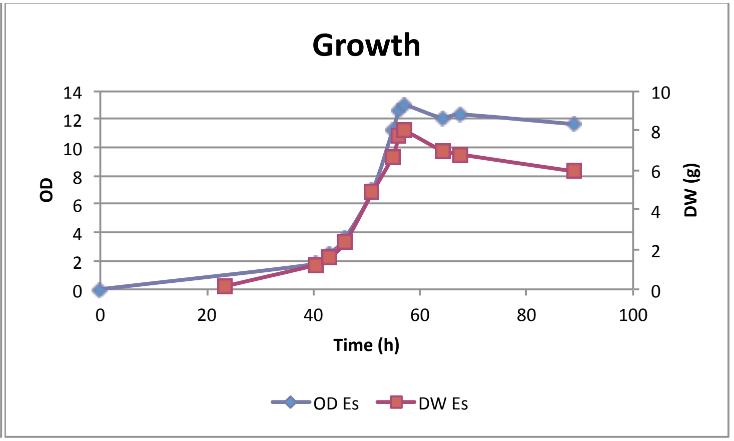
Growth
OD
DW (g)
Time (h)
OD Es
DW Es

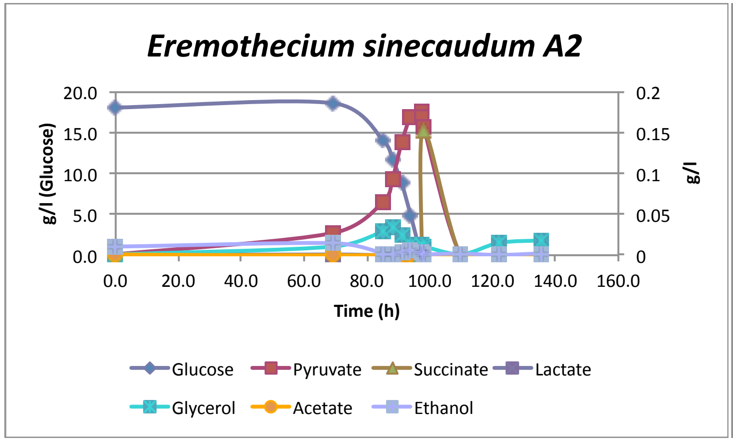
Eremothecium sinecaudum A2
g/l (Glucose)
g/l
Time (h)
Glucose
Pyruvate
Succinate
Lactate
Glycerol
Acetate
Ethanol

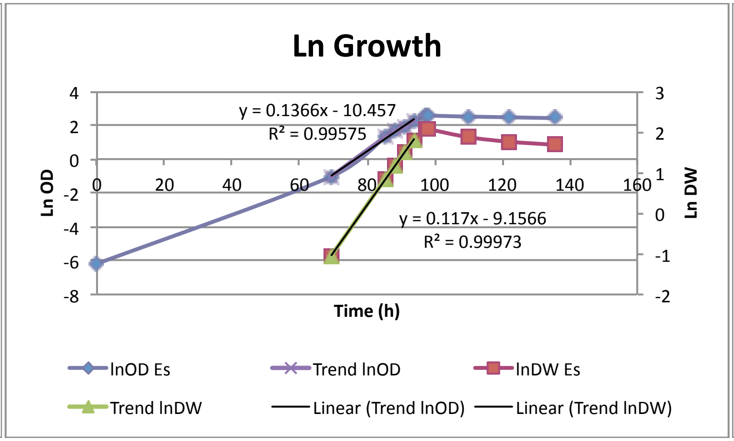
Ln Growth
y = 0.1366x - 10.457
R² = 0.99575
y = 0.117x - 9.1566
R² = 0.99973
Ln OD
Ln DW
Time (h)
lnOD Es
Trend lnOD
lnDW Es
Trend lnDW
Linear (Trend lnOD)
Linear (Trend lnDW)

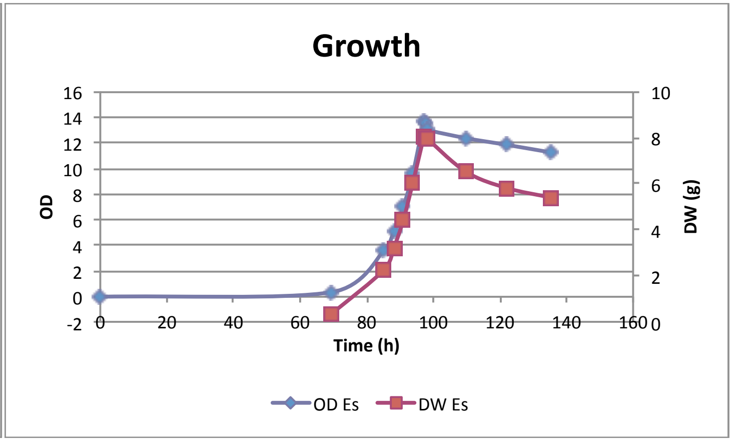
Growth
OD
DW (g)
Time (h)
OD Es
DW Es

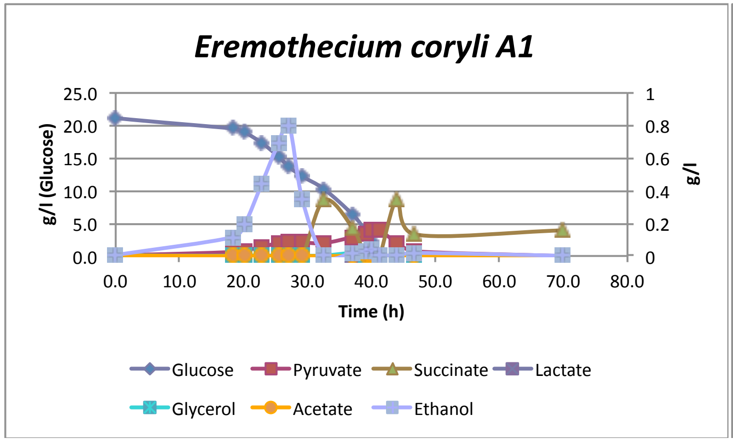
Eremothecium coryli A1
g/l (Glucose)
g/l
Time (h)
Glucose
Pyruvate
Succinate
Lactate
Glycerol
Acetate
Ethanol

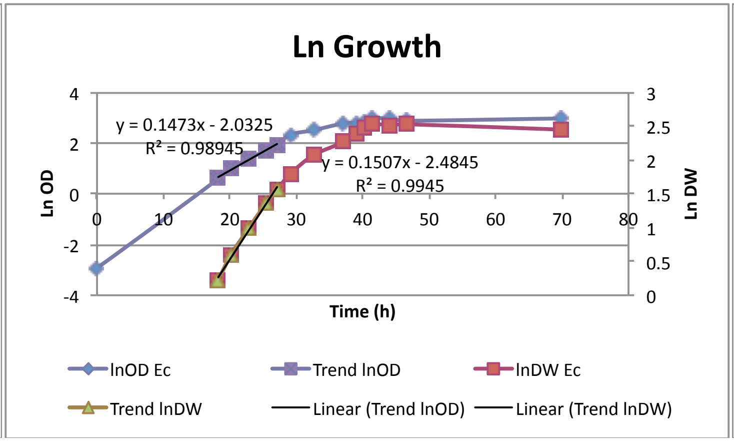
Ln Growth
y = 0.1473x - 2.0325
R² = 0.98945
y = 0.1507x - 2.4845
R² = 0.9945
Ln OD
Ln DW
Time (h)
lnOD Ec
Trend lnOD
lnDW Ec
Trend lnDW
Linear (Trend lnOD)
Linear (Trend lnDW)

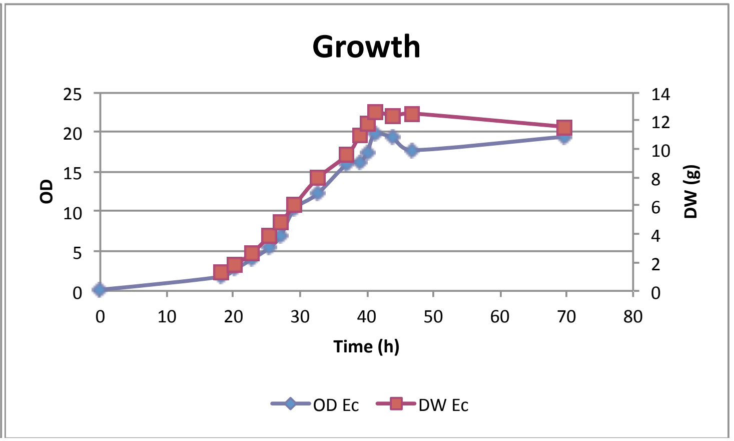
Growth
OD
DW (g)
Time (h)
OD Ec
DW Ec

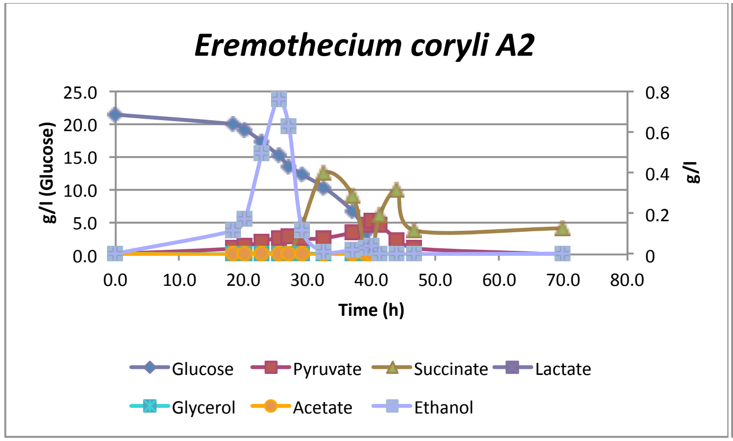
Eremothecium coryli A2
g/l (Glucose)
g/l
Time (h)
Glucose
Pyruvate
Succinate
Lactate
Glycerol
Acetate
Ethanol

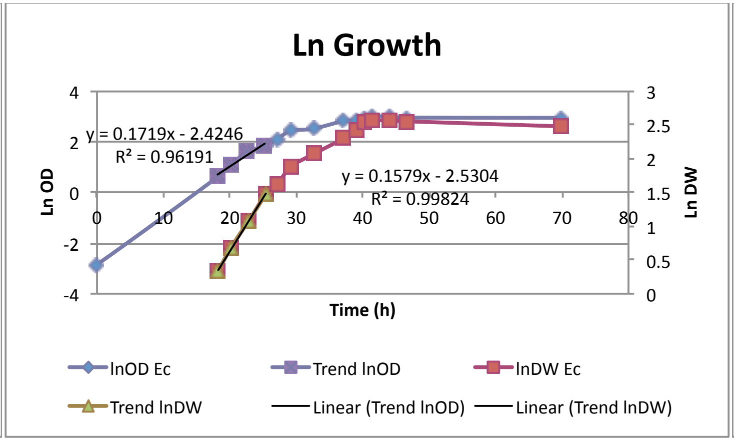
Ln Growth
y = 0.1719x - 2.4246
R² = 0.96191
y = 0.1579x - 2.5304
R² = 0.99824
Ln OD
Ln DW
Time (h)
lnOD Ec
Trend lnOD
lnDW Ec
Trend lnDW
Linear (Trend lnOD)
Linear (Trend lnDW)

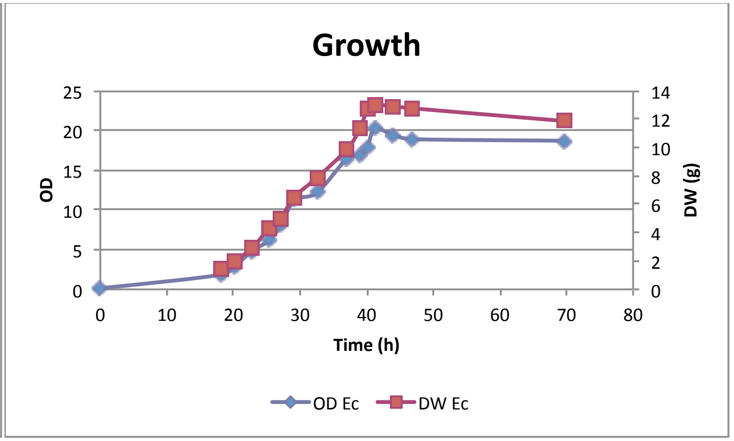
Growth
OD
DW (g)
Time (h)
OD Ec
DW Ec

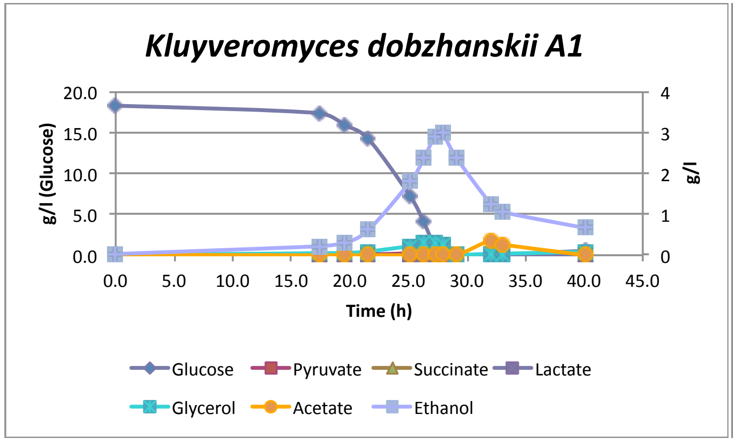
Kluyveromyces dobzhanskii A1
g/l (Glucose)
g/l
Time (h)
Glucose
Pyruvate
Succinate
Lactate
Glycerol
Acetate
Ethanol

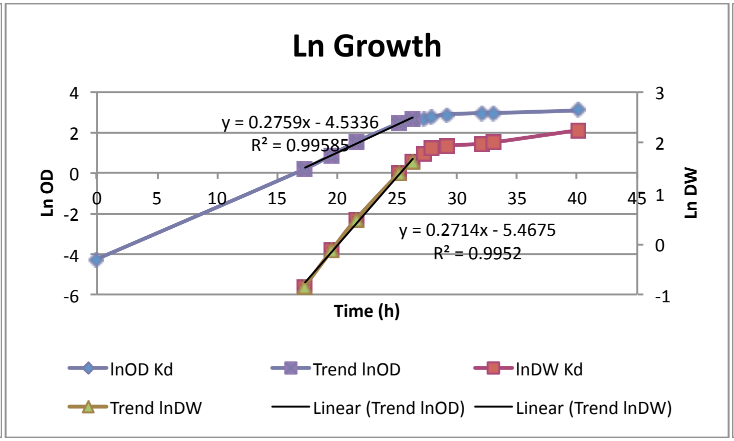
Ln Growth
y = 0.2759x - 4.5336
R² = 0.99585
y = 0.2714x - 5.4675
R² = 0.9952
Ln OD
Ln DW
Time (h)
lnOD Kd
Trend lnOD
lnDW Kd
Trend lnDW
Linear (Trend lnOD)
Linear (Trend lnDW)

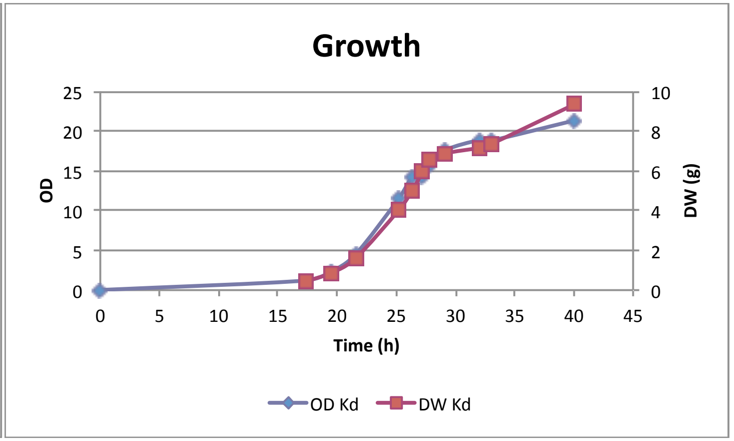
Growth
OD
DW (g)
Time (h)
OD Kd
DW Kd

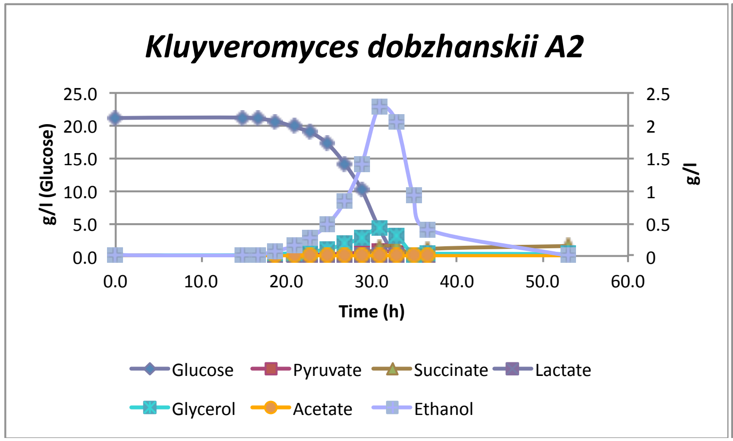
Kluyveromyces dobzhanskii A2
g/l (Glucose)
g/l
Time (h)
Glucose
Pyruvate
Succinate
Lactate
Glycerol
Acetate
Ethanol

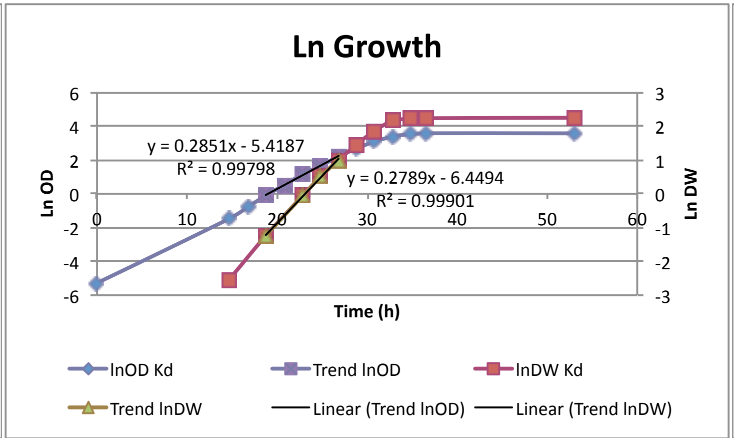
Ln Growth
y = 0.2851x - 5.4187
R² = 0.99798
y = 0.2789x - 6.4494
R² = 0.99901
Ln OD
Ln DW
Time (h)
lnOD Kd
Trend lnOD
lnDW Kd
Trend lnDW
Linear (Trend lnOD)
Linear (Trend lnDW)

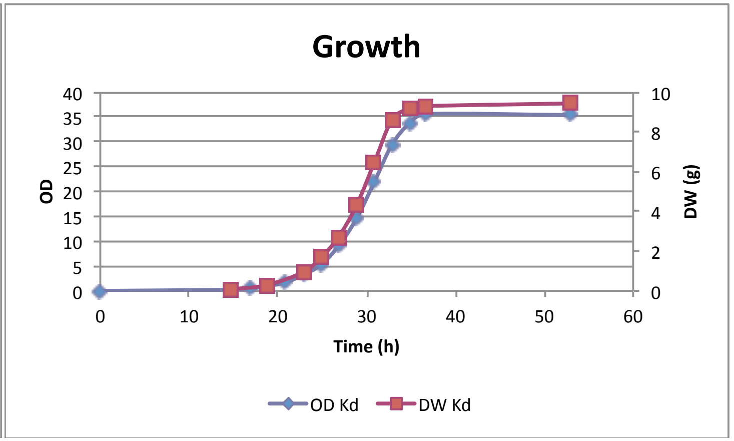
Growth
OD
DW (g)
Time (h)
OD Kd
DW Kd

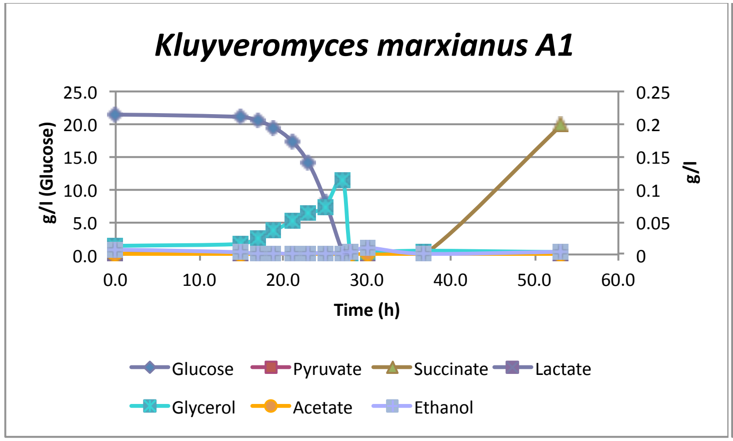

Kluyveromyces marxianus A1
g/l (Glucose)
g/l
Time (h)
Glucose
Pyruvate
Succinate
Lactate
Glycerol
Acetate
Ethanol

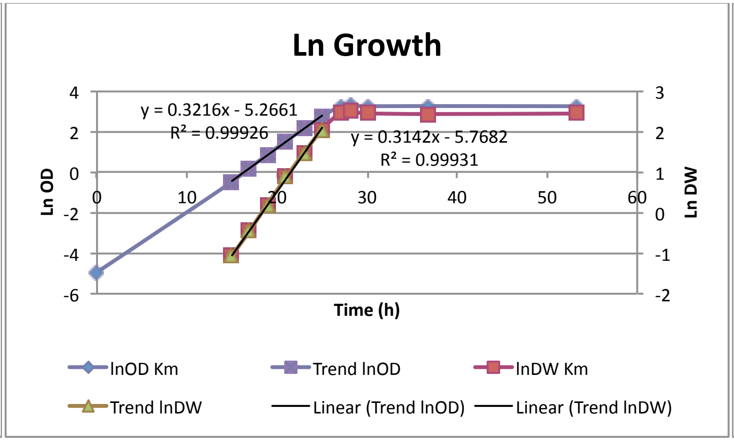

Ln Growth
y = 0.3216x - 5.2661
R² = 0.99926
y = 0.3142x - 5.7682
R² = 0.99931
Ln OD
Ln DW
Time (h)
lnOD Km
Trend lnOD
lnDW Km
Trend lnDW
Linear (Trend lnOD)
Linear (Trend lnDW)

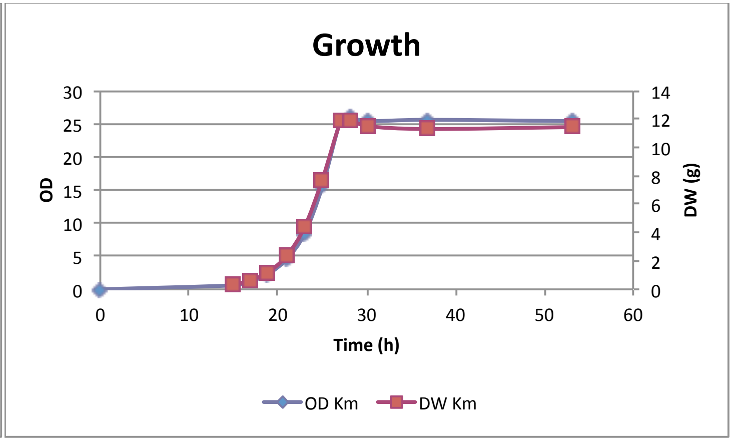

Growth
OD
DW (g)
Time (h)
OD Km
DW Km

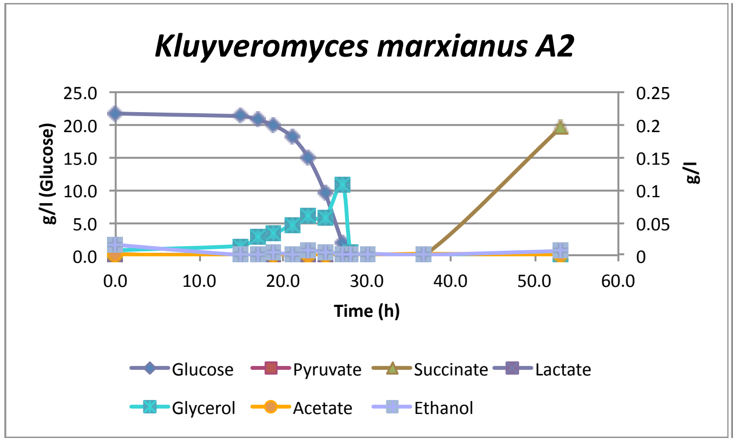

Kluyveromyces marxianus A2
g/l (Glucose)
g/l
Time (h)
Glucose
Pyruvate
Succinate
Lactate
Glycerol
Acetate
Ethanol

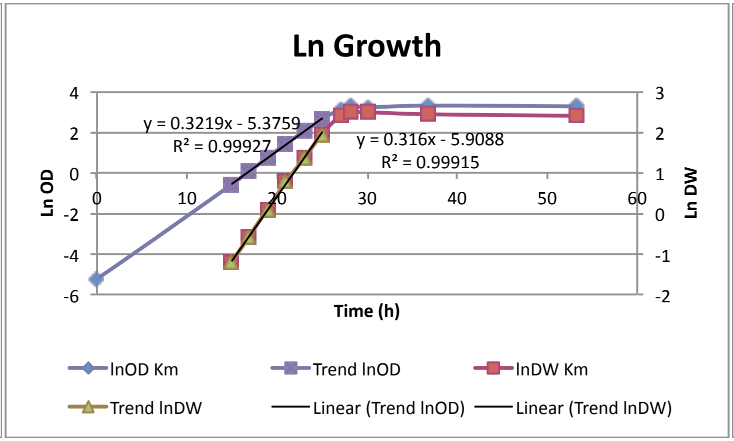

Ln Growth
y = 0.3219x - 5.3759
R² = 0.99927
y = 0.316x - 5.9088
R² = 0.99915
Ln OD
Ln DW
Time (h)
lnOD Km
Trend lnOD
lnDW Km
Trend lnDW
Linear (Trend lnOD)
Linear (Trend lnDW)

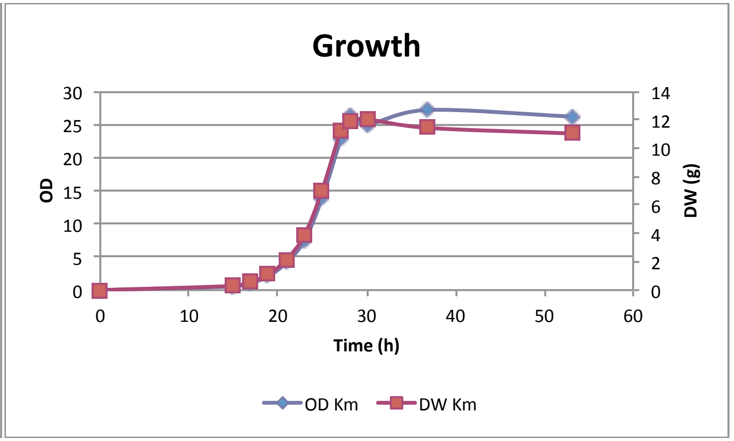

Growth
OD
DW (g)
Time (h)
OD Km
DW Km

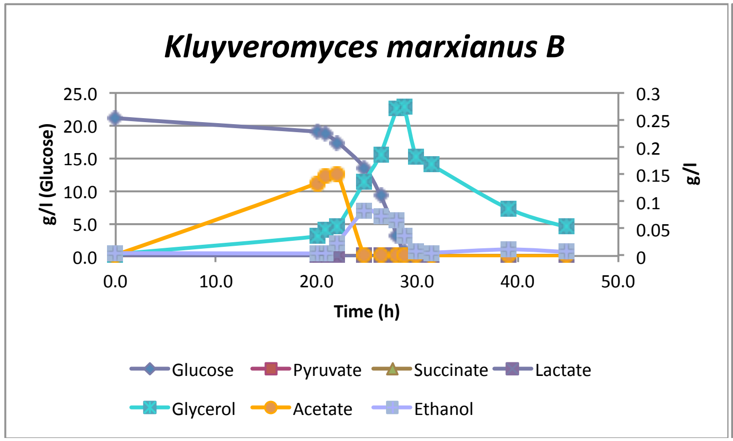

Kluyveromyces marxianus B
g/l (Glucose)
g/l
Time (h)
Glucose
Pyruvate
Succinate
Lactate
Glycerol
Acetate
Ethanol

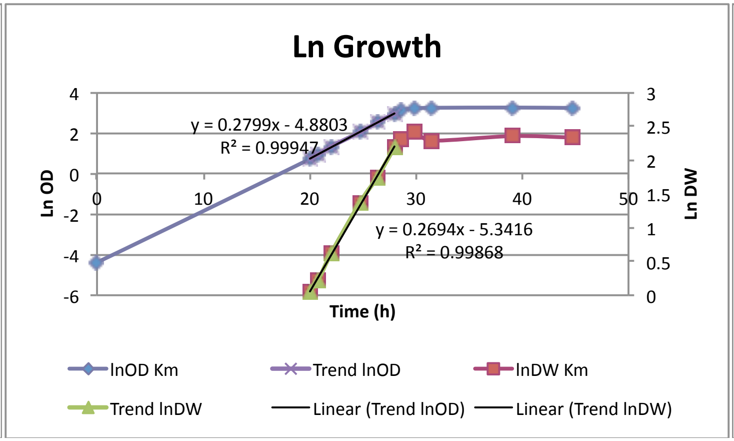

Ln Growth
y = 0.2799x - 4.8803
R² = 0.99947
y = 0.2694x - 5.3416
R² = 0.99868
Ln OD
Ln DW
Time (h)
lnOD Km
Trend lnOD
lnDW Km
Trend lnDW
Linear (Trend lnOD)
Linear (Trend lnDW)

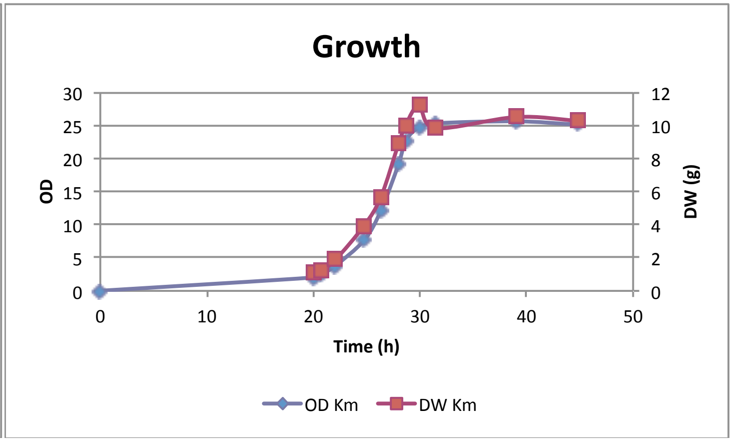

Growth
OD
DW (g)
Time (h)
OD Km
DW Km

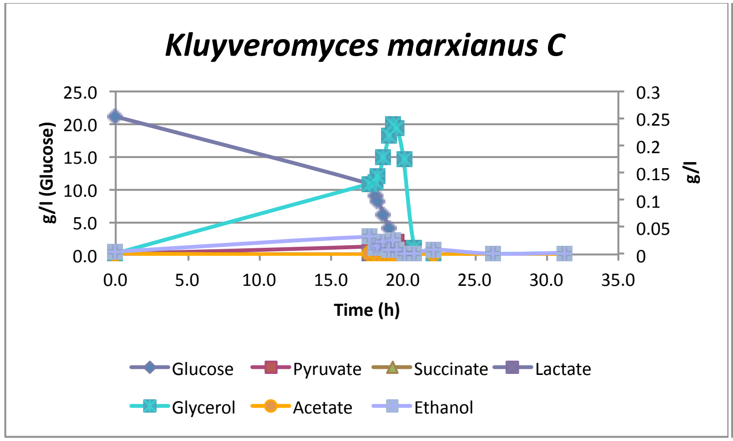
Kluyveromyces marxianus C
g/l (Glucose)
g/l
Time (h)
Glucose
Pyruvate
Succinate
Lactate
Glycerol
Acetate
Ethanol

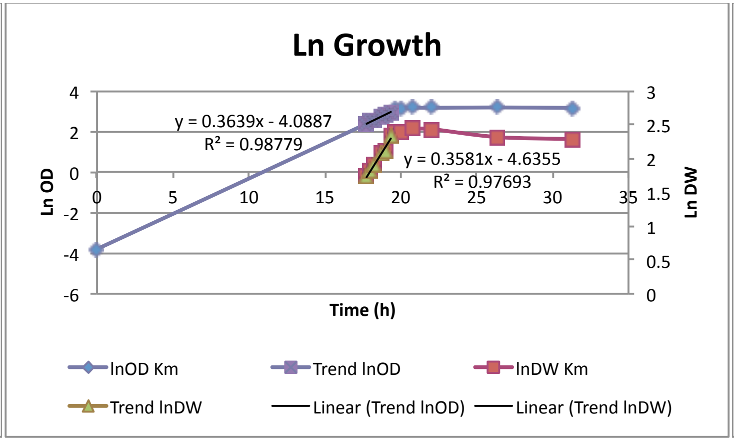
Ln Growth
y = 0.3639x - 4.0887
R² = 0.98779
y = 0.3581x - 4.6355
R² = 0.97693
Ln OD
Ln DW
Time (h)
lnOD Km
Trend lnOD
lnDW Km
Trend lnDW
Linear (Trend lnOD)
Linear (Trend lnDW)

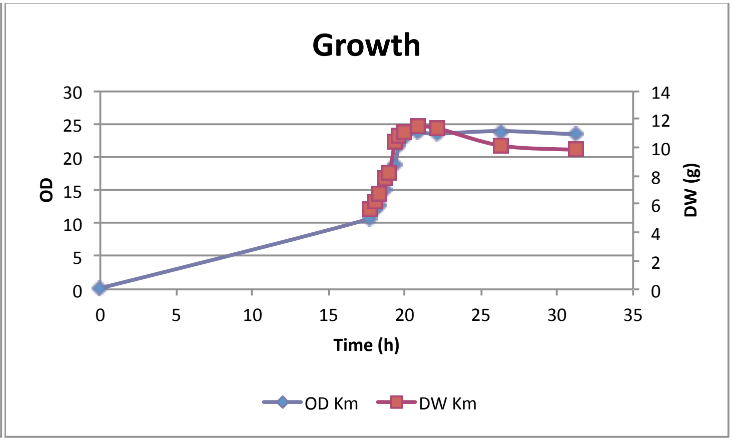
Growth
OD
DW (g)
Time (h)
OD Km
DW Km

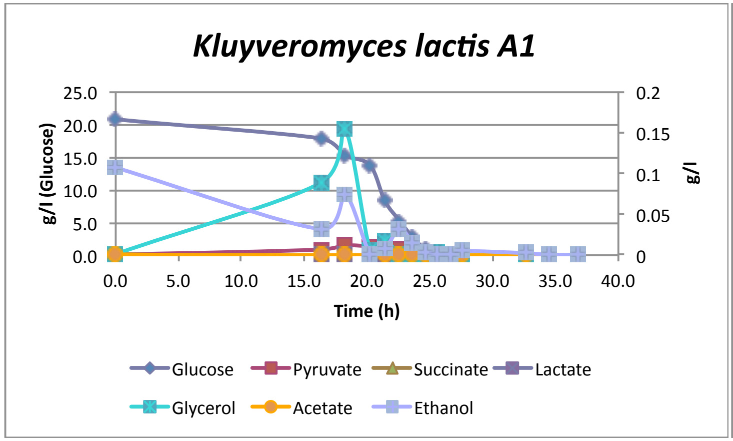
Kluyveromyces lactis A1
g/l (Glucose)
g/l
Time (h)
Glucose
Pyruvate
Succinate
Lactate
Glycerol
Acetate
Ethanol

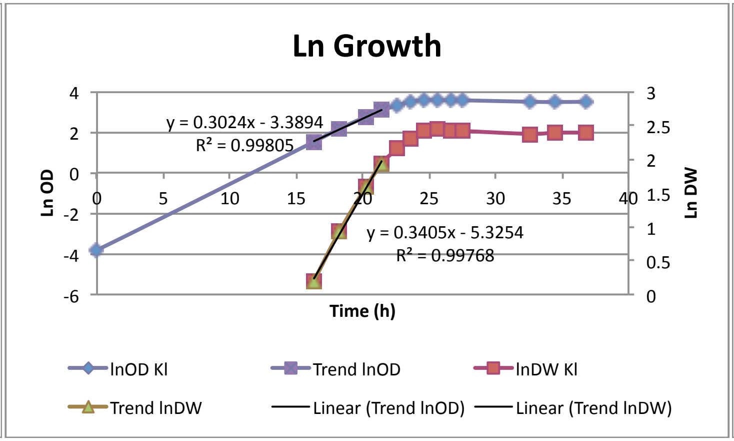
Ln Growth
y = 0.3024x - 3.3894
R² = 0.99805
y = 0.3405x - 5.3254
R² = 0.99768
Ln OD
Ln DW
Time (h)
lnOD Kl
Trend lnOD
lnDW Kl
Trend lnDW
Linear (Trend lnOD)
Linear (Trend lnDW)

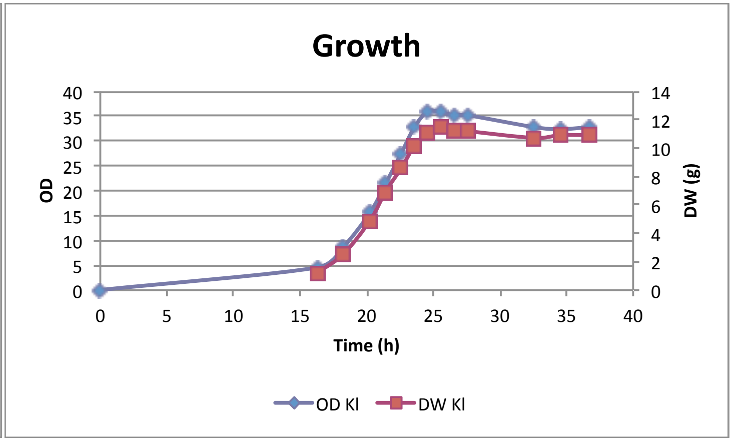
Growth
OD
DW (g)
Time (h)
OD Kl
DW Kl

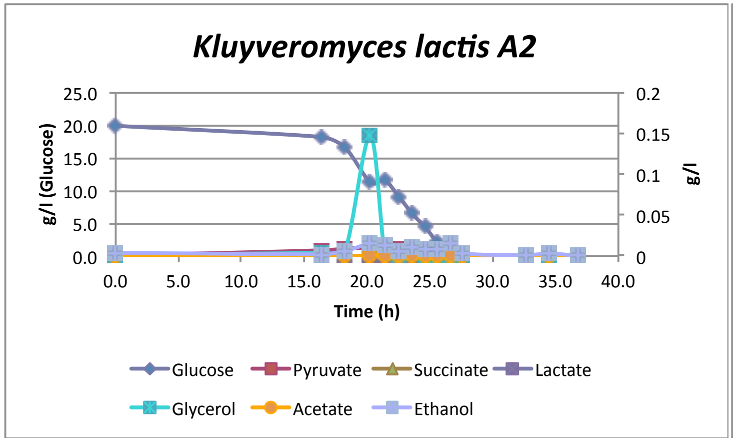
Kluyveromyces lactis A2
g/l (Glucose)
g/l
Time (h)
Glucose
Pyruvate
Succinate
Lactate
Glycerol
Acetate
Ethanol

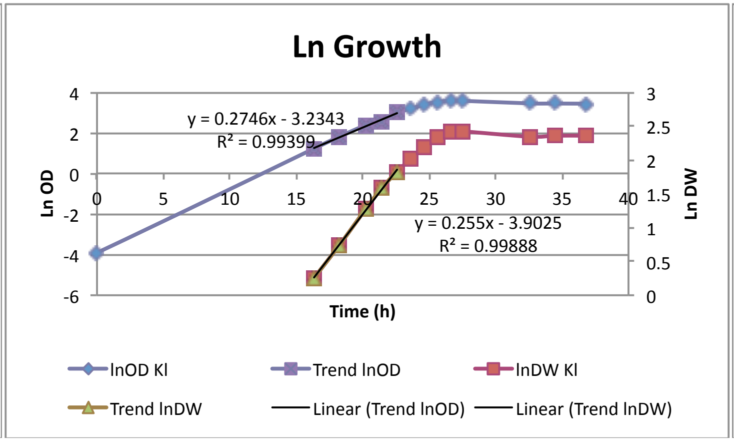
Ln Growth
y = 0.2746x - 3.2343
R² = 0.99399
y = 0.255x - 3.9025
R² = 0.99888
Ln OD
Ln DW
Time (h)
lnOD Kl
Trend lnOD
lnDW Kl
Trend lnDW
Linear (Trend lnOD)
Linear (Trend lnDW)

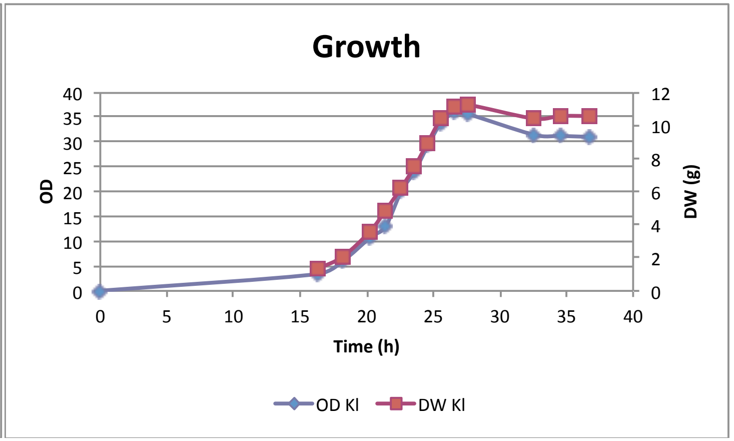
Growth
OD
DW (g)
Time (h)
OD Kl
DW Kl

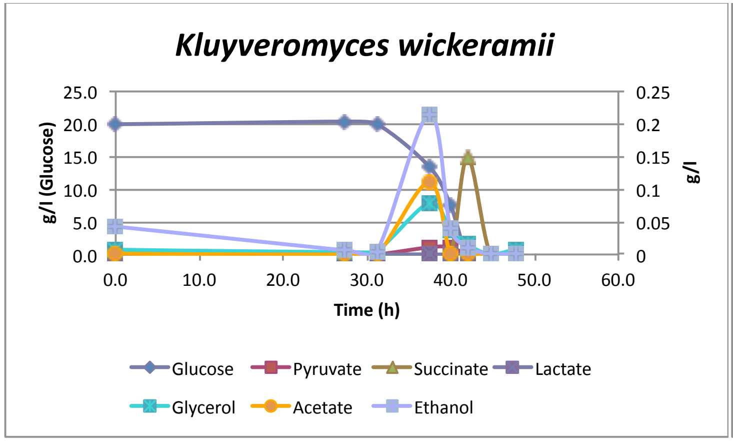
Kluyveromyces wickeramii
g/l (Glucose)
g/l
Time (h)
Glucose
Pyruvate
Succinate
Lactate
Glycerol
Acetate
Ethanol

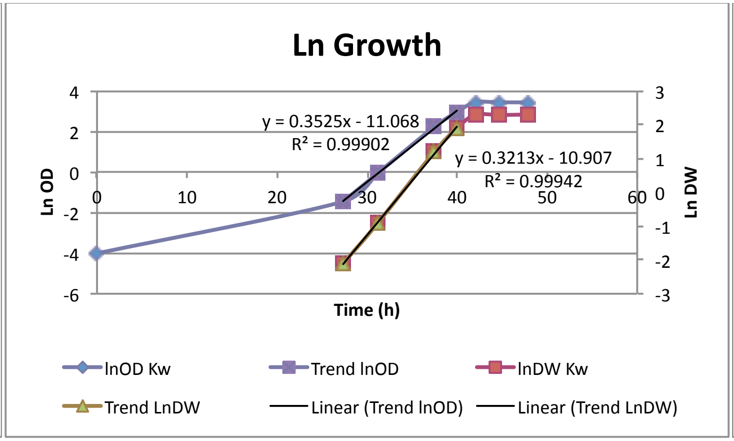
Ln Growth
y = 0.3525x - 11.068
R² = 0.99902
y = 0.3213x - 10.907
R² = 0.99942
Ln OD
Ln DW
Time (h)
lnOD Kw
Trend lnOD
lnDW Kw
Trend LnDW
Linear (Trend lnOD)
Linear (Trend LnDW)

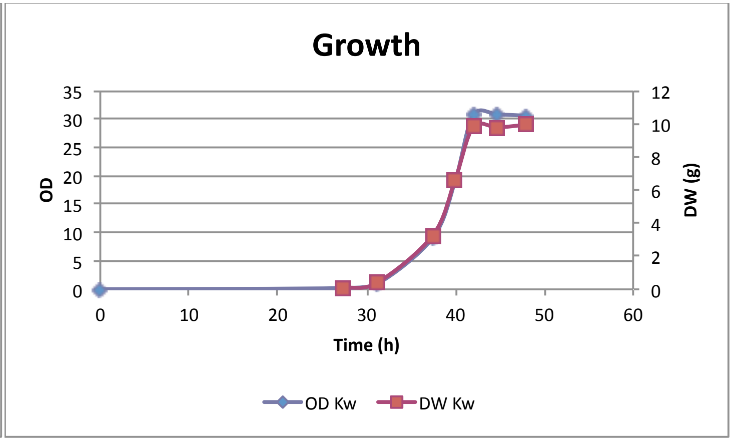
Growth
OD
DW (g)
Time (h)
OD Kw
DW Kw

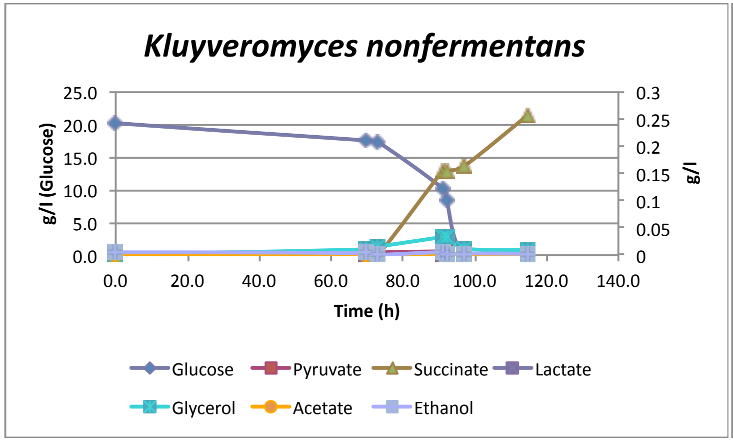
Kluyveromyces nonfermentans
g/l (Glucose)
g/l
Time (h)
Glucose
Pyruvate
Succinate
Lactate
Glycerol
Acetate
Ethanol

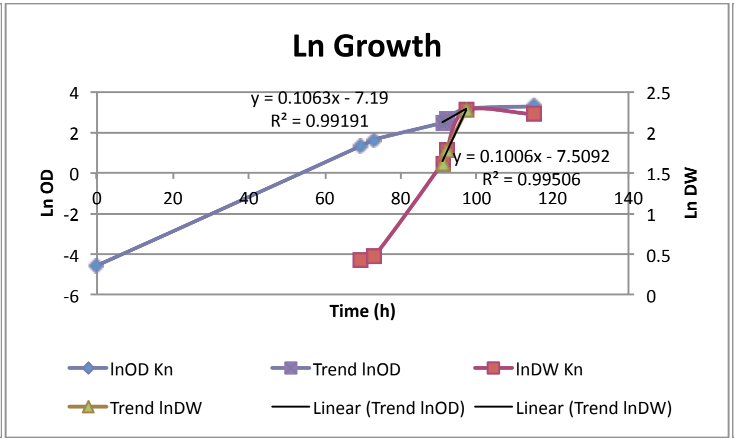
Ln Growth
y = 0.1063x - 7.19
R² = 0.99191
y = 0.1006x - 7.5092
R² = 0.99506
Ln OD
Ln DW
Time (h)
lnOD Kn
Trend lnOD
lnDW Kn
Trend lnDW
Linear (Trend lnOD)
Linear (Trend lnDW)

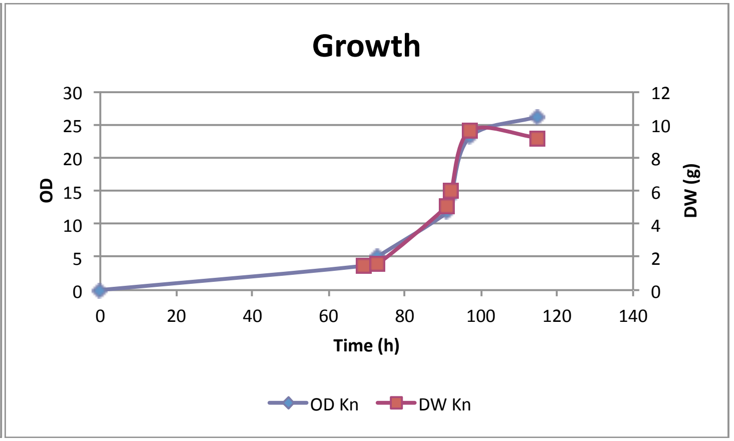
Growth
OD
DW (g)
Time (h)
OD Kn
DW Kn

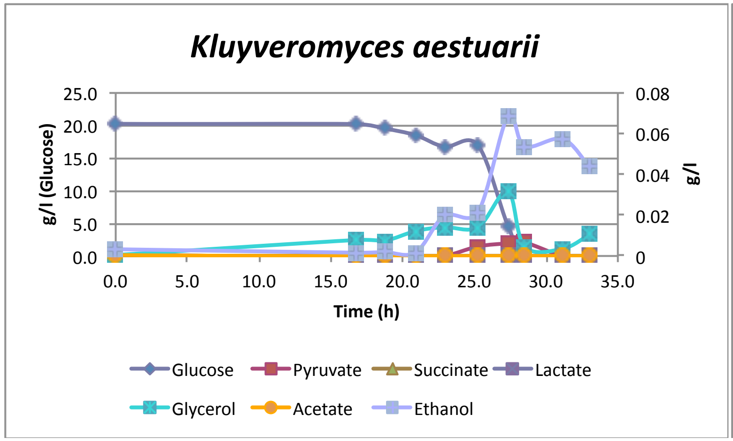
Kluyveromyces aestuarii
g/l (Glucose)
g/l
Time (h)
Glucose
Pyruvate
Succinate
Lactate
Glycerol
Acetate
Ethanol

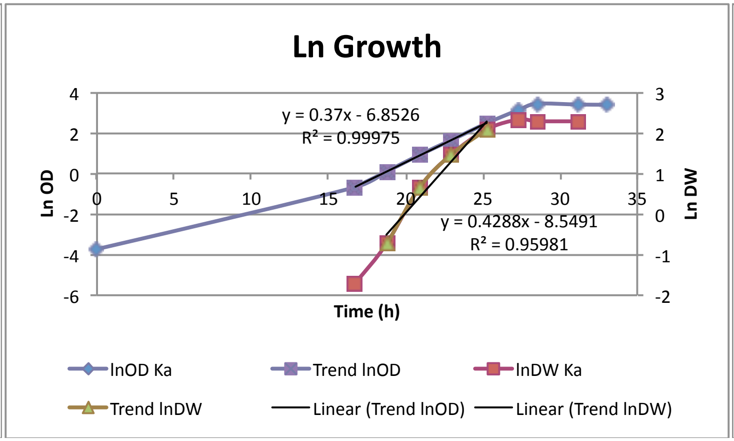
Ln Growth
y = 0.37x - 6.8526
R² = 0.99975
y = 0.4288x - 8.5491
R² = 0.95981
Ln OD
Ln DW
Time (h)
lnOD Ka
Trend lnOD
lnDW Ka
Trend lnDW
Linear (Trend lnOD)
Linear (Trend lnDW)

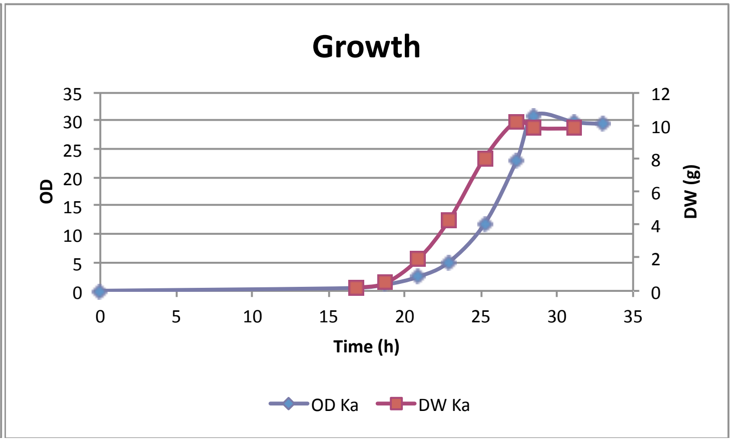
Growth
OD
DW (g)
Time (h)
OD Ka
DW Ka

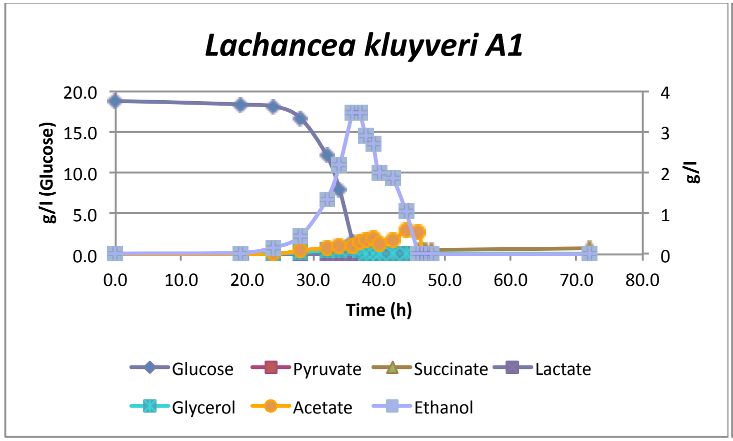
Lachancea kluyveri A1
g/l (Glucose)
g/l
Time (h)
Glucose
Pyruvate
Succinate
Lactate
Glycerol
Acetate
Ethanol

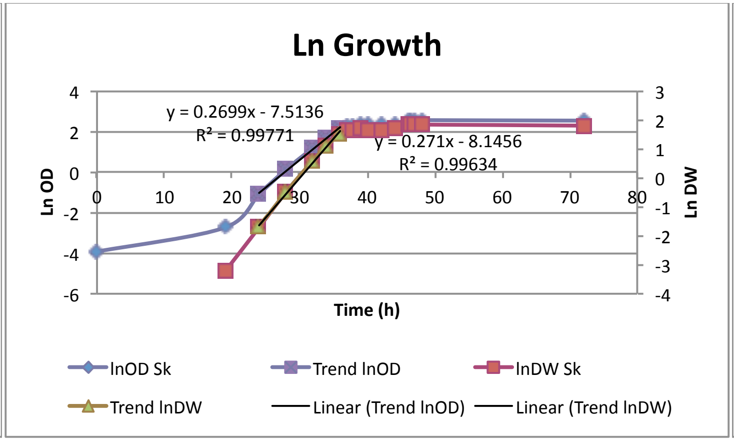
Ln Growth
y = 0.2699x - 7.5136
R² = 0.99771
y = 0.271x - 8.1456
R² = 0.99634
Ln OD
Ln DW
Time (h)
lnOD Sk
Trend lnOD
lnDW Sk
Trend lnDW
Linear (Trend lnOD)
Linear (Trend lnDW)

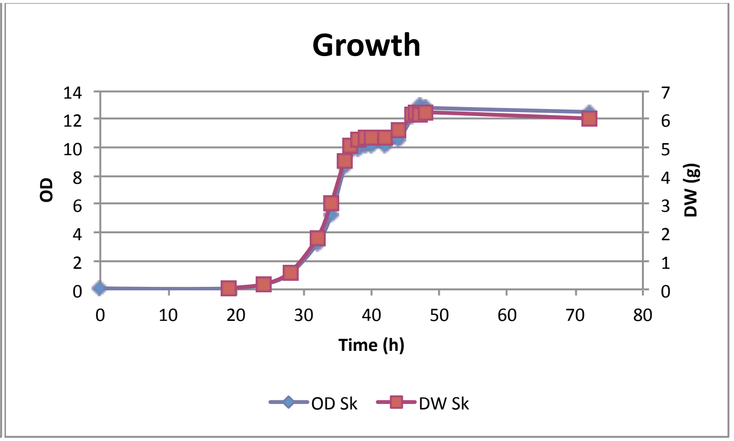
Growth
OD
DW (g)
Time (h)
OD Sk
DW Sk

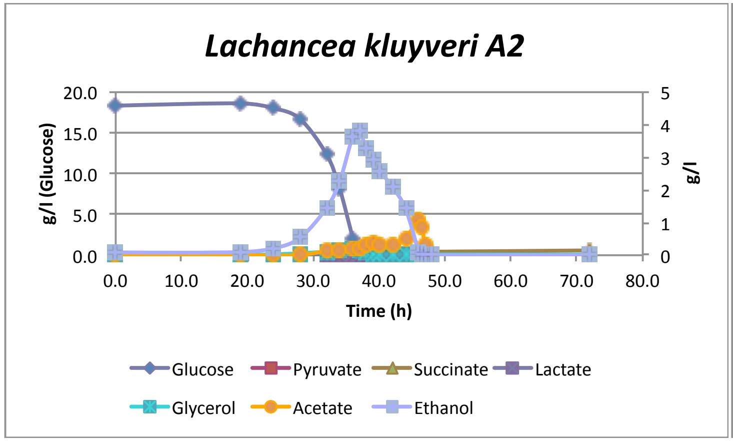
Lachancea kluyveri A2
g/l (Glucose)
g/l
Time (h)
Glucose
Pyruvate
Succinate
Lactate
Glycerol
Acetate
Ethanol

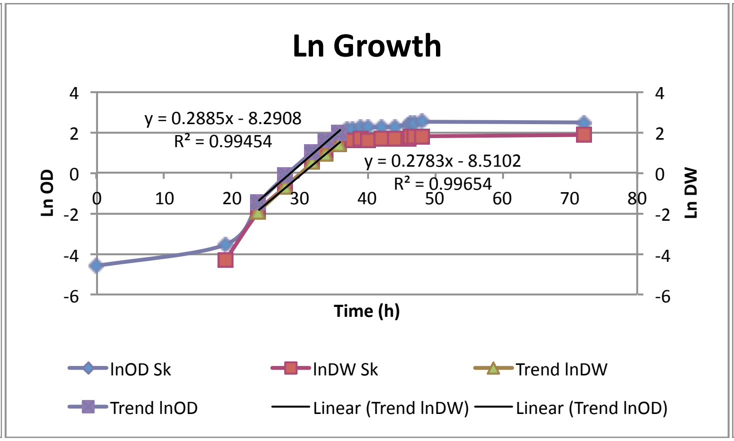
Ln Growth
y = 0.2885x - 8.2908
R² = 0.99454
y = 0.2783x - 8.5102
R² = 0.99654
Ln OD
Ln DW
Time (h)
lnOD Sk
lnDW Sk
Trend lnDW
Trend lnOD
Linear (Trend lnDW)
Linear (Trend lnOD)

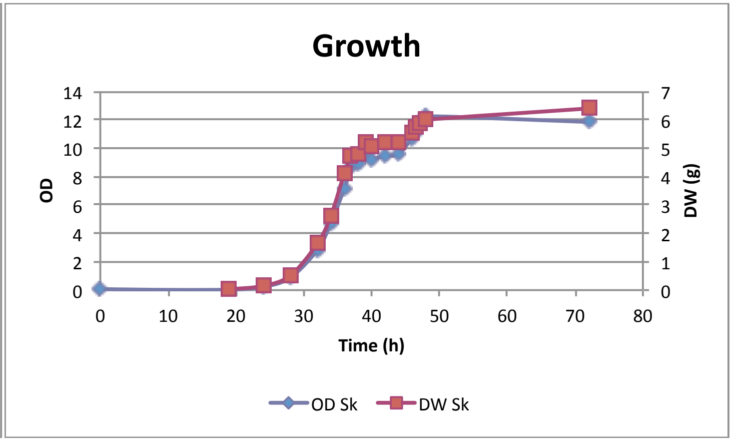
Growth
OD
DW (g)
Time (h)
OD Sk
DW Sk

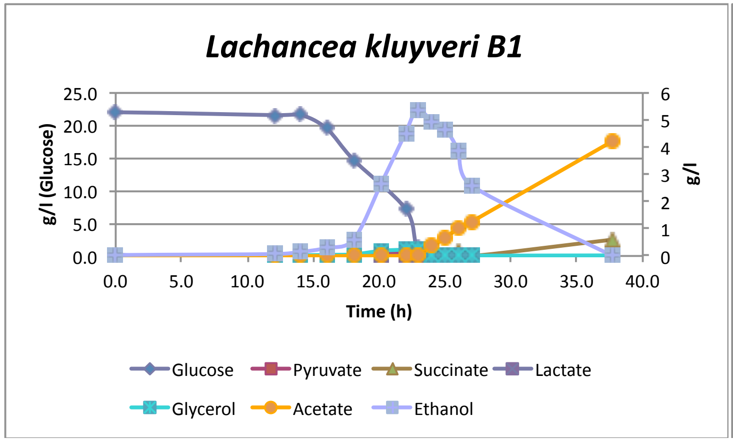
Lachancea kluyveri B1
g/l (Glucose)
g/l
Time (h)
Glucose
Pyruvate
Succinate
Lactate
Glycerol
Acetate
Ethanol

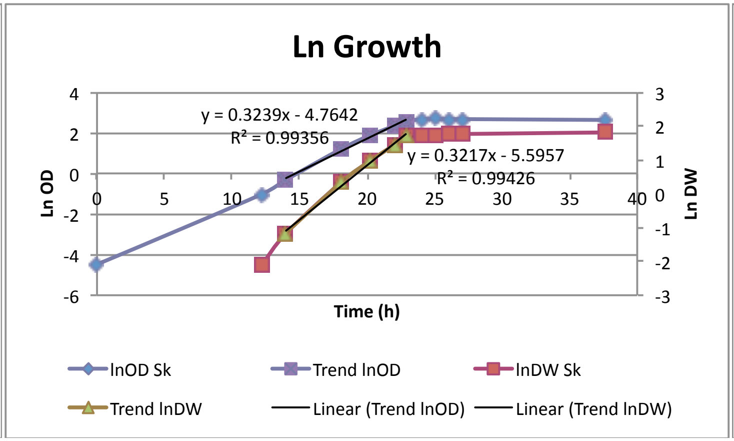
Ln Growth
y = 0.3239x - 4.7642
R² = 0.99356
y = 0.3217x - 5.5957
R² = 0.99426
Ln OD
Ln DW
Time (h)
lnOD Sk
Trend lnOD
lnDW Sk
Trend lnDW
Linear (Trend lnOD)
Linear (Trend lnDW)

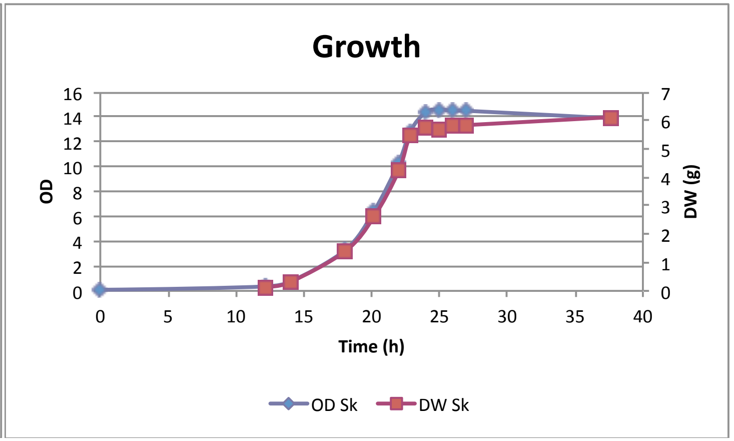
Growth
OD
DW (g)
Time (h)
OD Sk
DW Sk

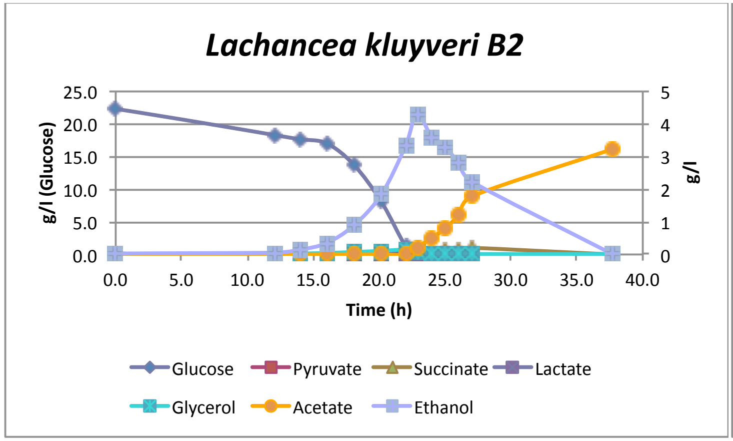
Lachancea kluyveri B2
g/l (Glucose)
g/l
Time (h)
Glucose
Pyruvate
Succinate
Lactate
Glycerol
Acetate
Ethanol

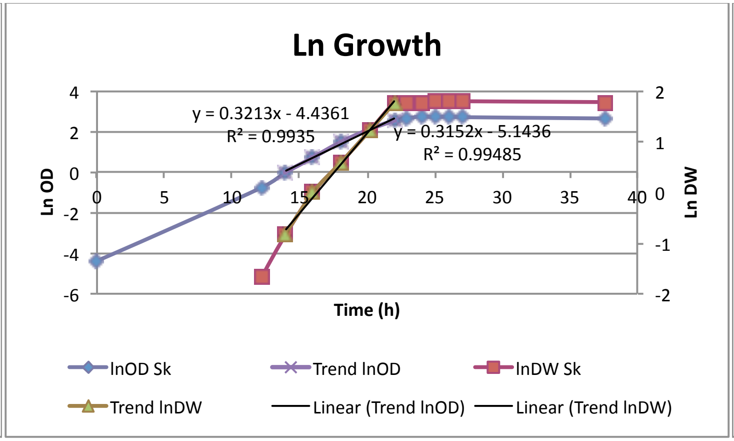
Ln Growth
y = 0.3213x - 4.4361
R^2 = 0.9935
y = 0.3152x - 5.1436
R^2 = 0.99485
Ln OD
Ln DW
Time (h)
lnOD Sk
Trend lnOD
lnDW Sk
Trend lnDW
Linear (Trend lnOD)
Linear (Trend lnDW)

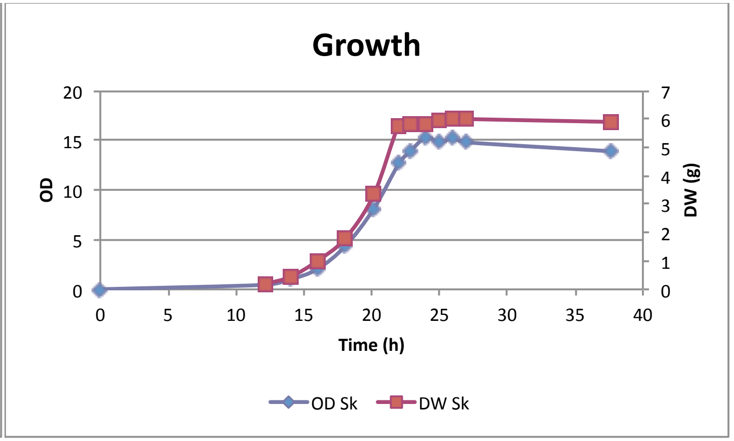
Growth
OD
DW (g)
Time (h)
OD Sk
DW Sk

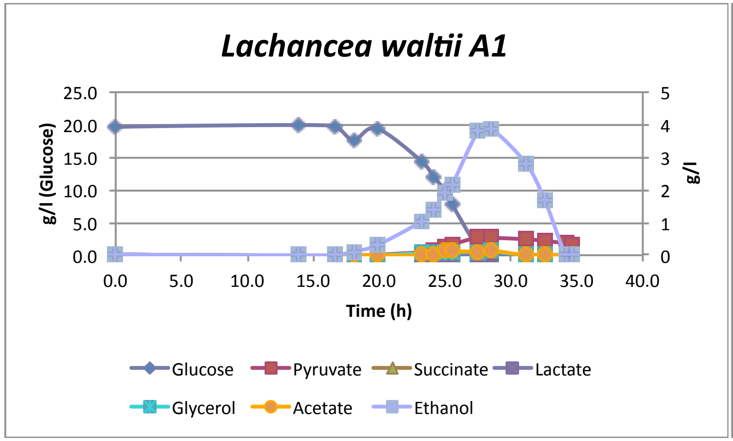
Lachancea waltii A1
g/l (Glucose)
g/l
Time (h)
Glucose
Pyruvate
Succinate
Lactate
Glycerol
Acetate
Ethanol

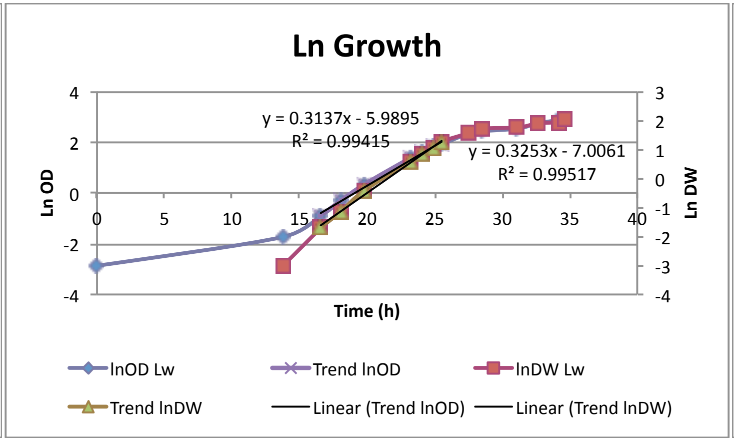
Ln Growth
y = 0.3137x - 5.9895
R^2 = 0.99415
y = 0.3253x - 7.0061
R^2 = 0.99517
Ln OD
Ln DW
Time (h)
lnOD Lw
Trend lnOD
lnDW Lw
Trend lnDW
Linear (Trend lnOD)
Linear (Trend lnDW)

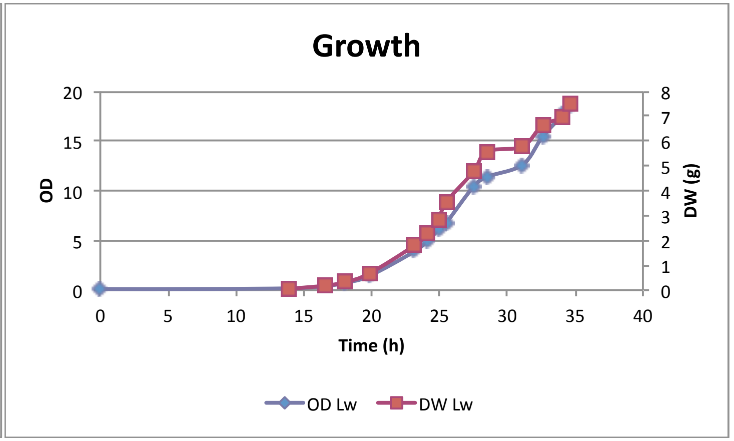
Growth
OD
DW (g)
Time (h)
OD Lw
DW Lw

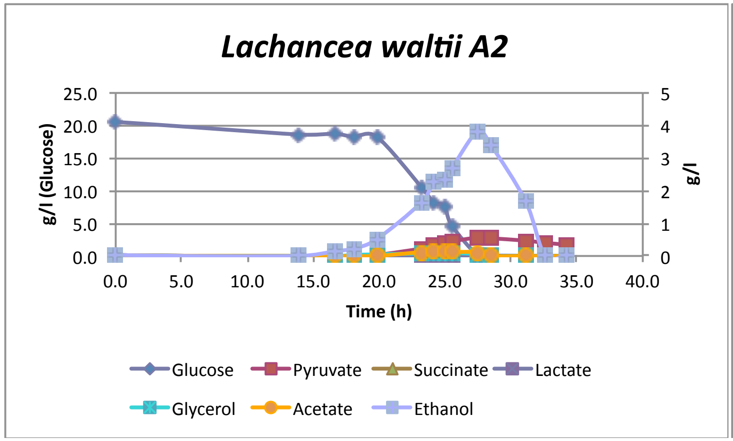
Lachancea waltii A2
g/l (Glucose)
g/l
Time (h)
Glucose
Pyruvate
Succinate
Lactate
Glycerol
Acetate
Ethanol

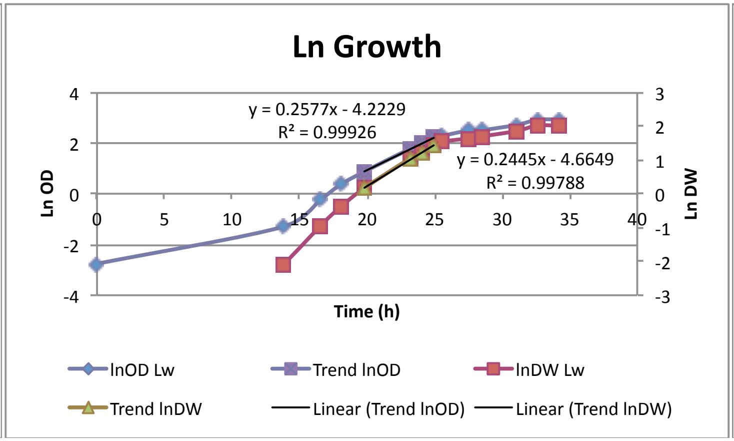
Ln Growth
y = 0.2577x - 4.2229
R^2 = 0.99926
y = 0.2445x - 4.6649
R^2 = 0.99788
Ln OD
Ln DW
Time (h)
lnOD Lw
Trend lnOD
lnDW Lw
Trend lnDW
Linear (Trend lnOD)
Linear (Trend lnDW)

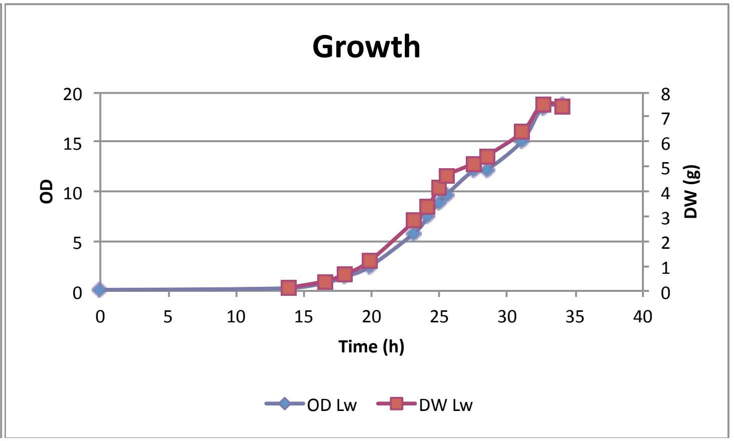
Growth
OD
DW (g)
Time (h)
OD Lw
DW Lw

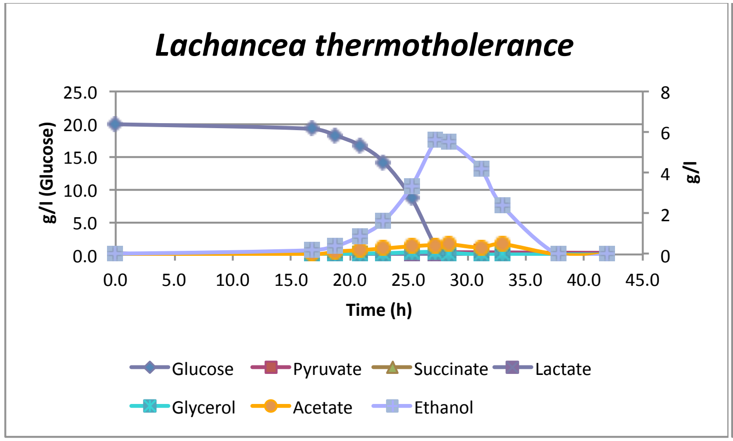
Lachancea thermotholerance
g/l (Glucose)
g/l
Time (h)
Glucose
Pyruvate
Succinate
Lactate
Glycerol
Acetate
Ethanol

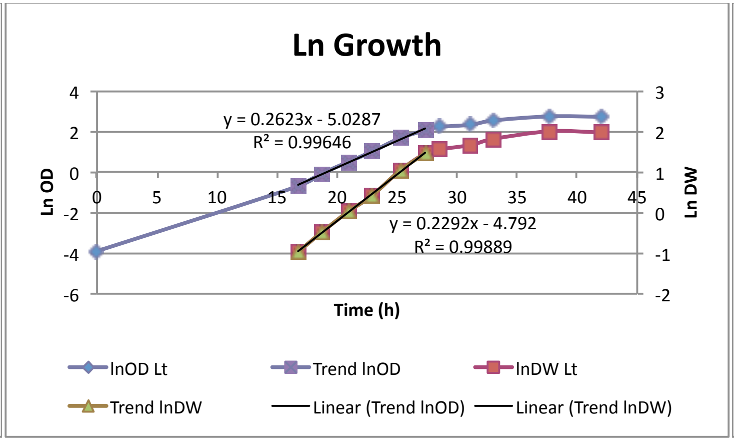
Ln Growth
y = 0.2623x - 5.0287
R² = 0.99646
y = 0.2292x - 4.792
R² = 0.99889
Ln OD
Ln DW
Time (h)
lnOD Lt
Trend lnOD
lnDW Lt
Trend lnDW
Linear (Trend lnOD)
Linear (Trend lnDW)

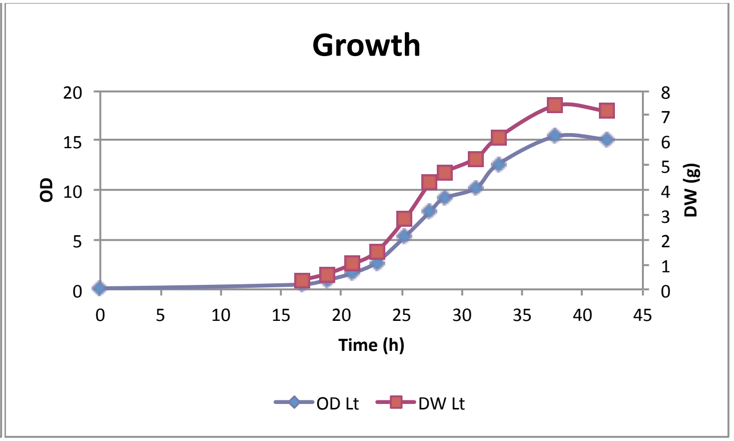
Growth
OD
DW (g)
Time (h)
OD Lt
DW Lt

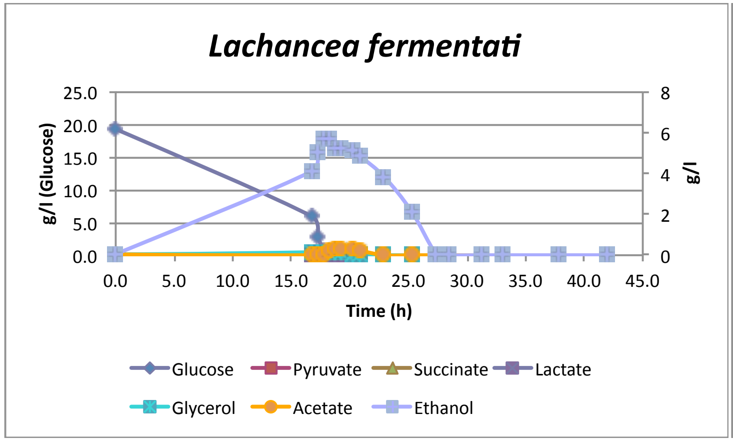
Lachancea fermentati
g/l (Glucose)
g/l
Time (h)
Glucose
Pyruvate
Succinate
Lactate
Glycerol
Acetate
Ethanol

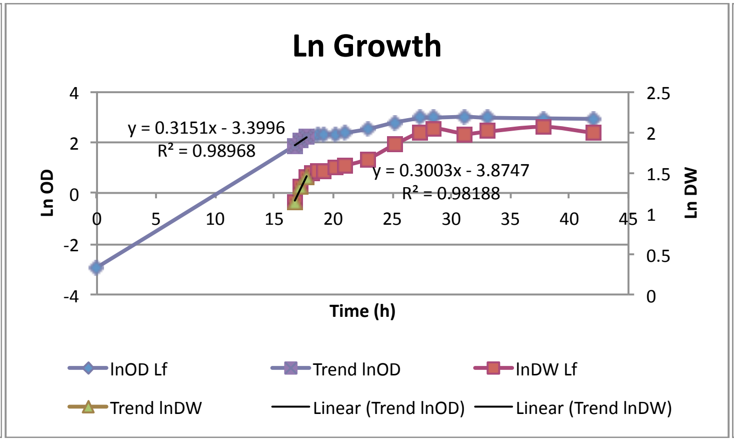
Ln Growth
y = 0.3151x - 3.3996
R² = 0.98968
y = 0.3003x - 3.8747
R² = 0.98188
Ln OD
Ln DW
Time (h)
lnOD Lf
Trend lnOD
lnDW Lf
Trend lnDW
Linear (Trend lnOD)
Linear (Trend lnDW)

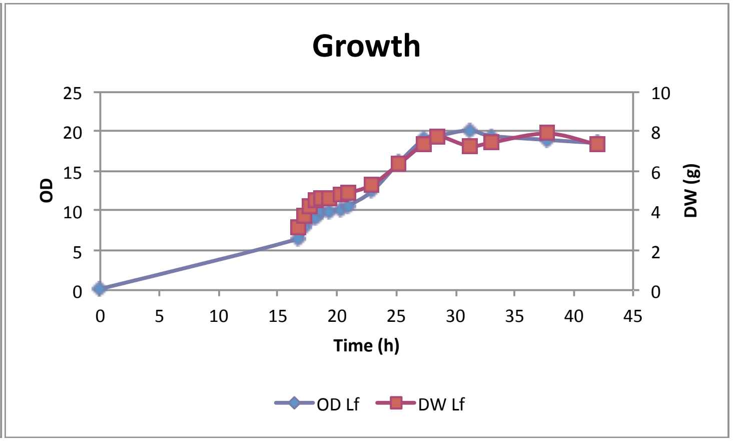
Growth
OD
DW (g)
Time (h)
OD Lf
DW Lf

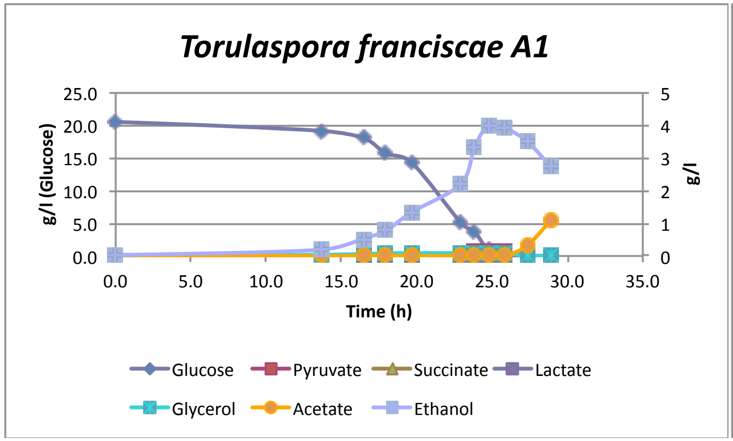
Torulaspora franciscae A1
g/l (Glucose)
g/l
Time (h)
Glucose
Pyruvate
Succinate
Lactate
Glycerol
Acetate
Ethanol

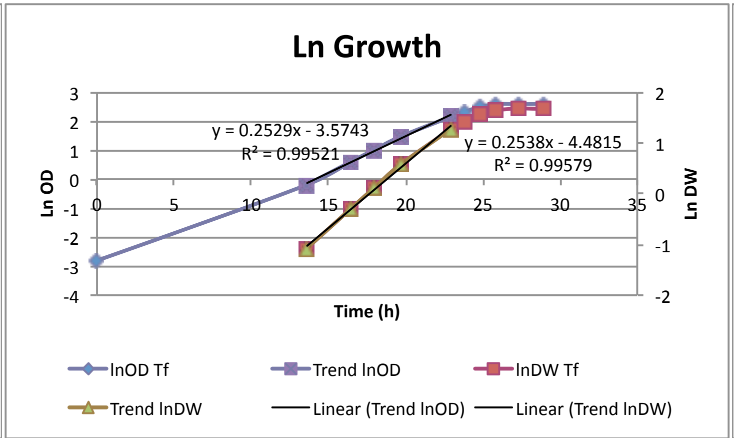
Ln Growth
y = 0.2529x - 3.5743
R² = 0.99521
y = 0.2538x - 4.4815
R² = 0.99579
Ln OD
Ln DW
Time (h)
lnOD Tf
Trend lnOD
lnDW Tf
Trend lnDW
Linear (Trend lnOD)
Linear (Trend lnDW)

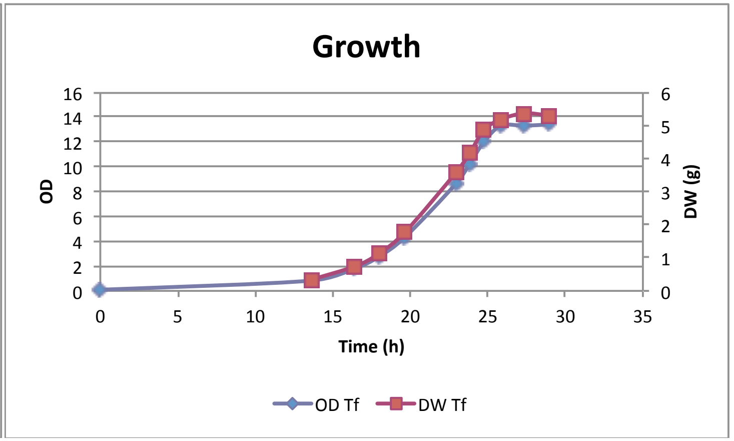
Growth
OD
DW (g)
Time (h)
OD Tf
DW Tf

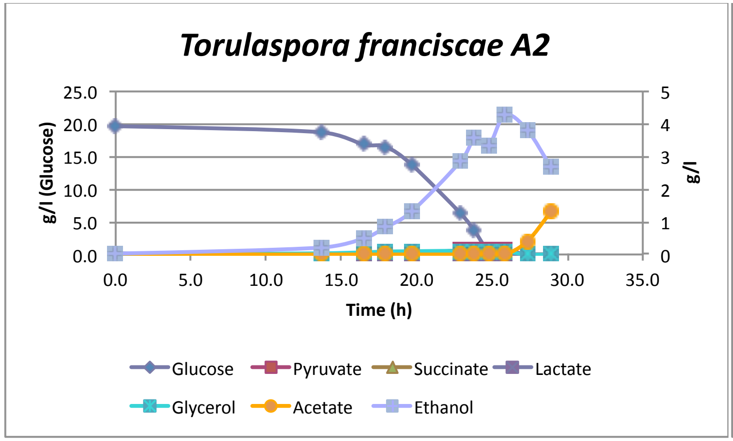
Torulaspora franciscae A2
g/l (Glucose)
g/l
Time (h)
Glucose
Pyruvate
Succinate
Lactate
Glycerol
Acetate
Ethanol

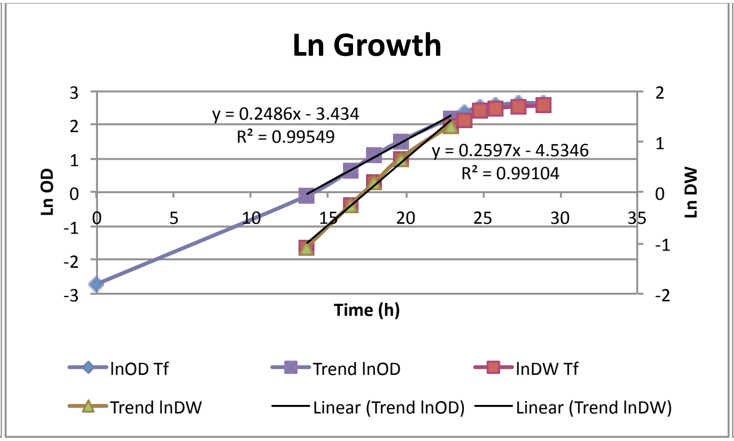
Ln Growth
y = 0.2486x - 3.434
R² = 0.99549
y = 0.2597x - 4.5346
R² = 0.99104
Ln OD
Ln DW
Time (h)
lnOD Tf
Trend lnOD
lnDW Tf
Trend lnDW
Linear (Trend lnOD)
Linear (Trend lnDW)

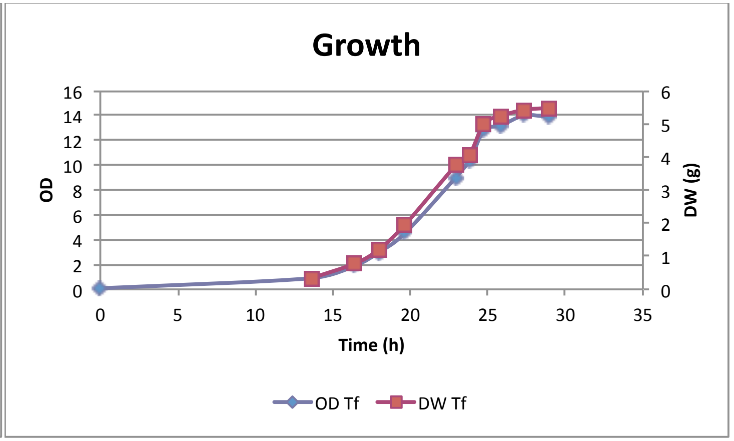
Growth
OD
DW (g)
Time (h)
OD Tf
DW Tf

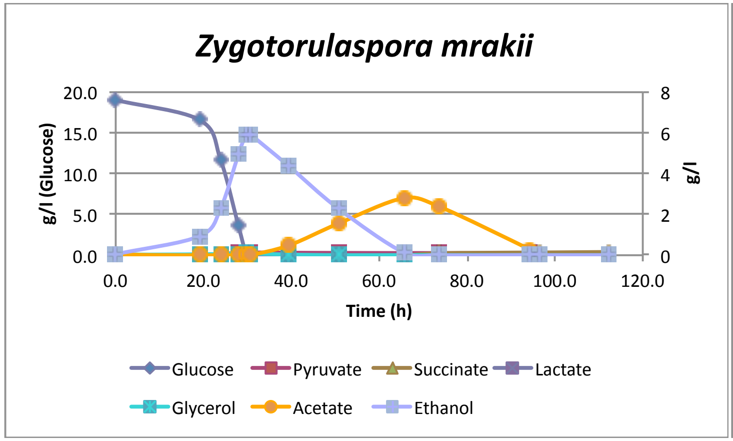
Zygotorulaspora mrakii
g/l (Glucose)
g/l
Time (h)
Glucose
Pyruvate
Succinate
Lactate
Glycerol
Acetate
Ethanol

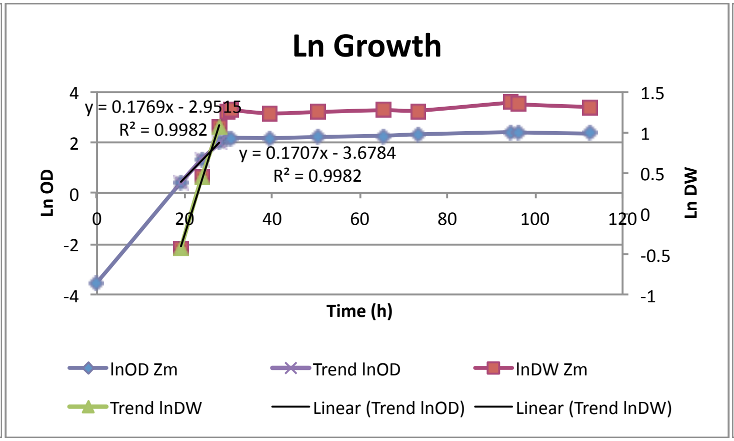
Ln Growth
y = 0.1769x - 2.9515
R² = 0.9982
y = 0.1707x - 3.6784
R² = 0.9982
Ln OD
Ln DW
Time (h)
lnOD Zm
Trend lnOD
lnDW Zm
Trend lnDW
Linear (Trend lnOD)
Linear (Trend lnDW)

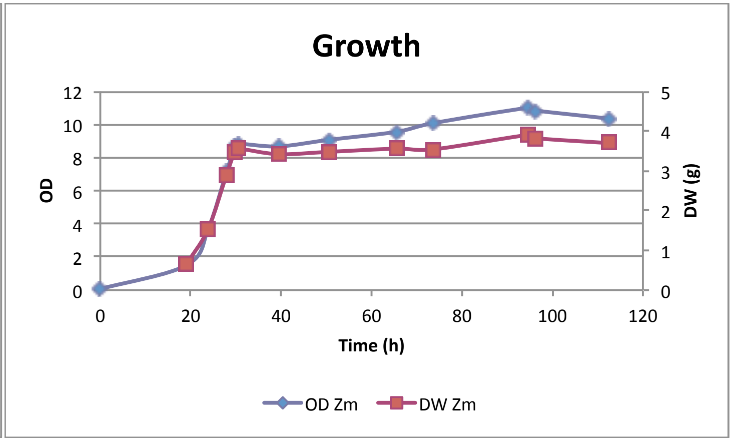
Growth
OD
DW (g)
Time (h)
OD Zm
DW Zm

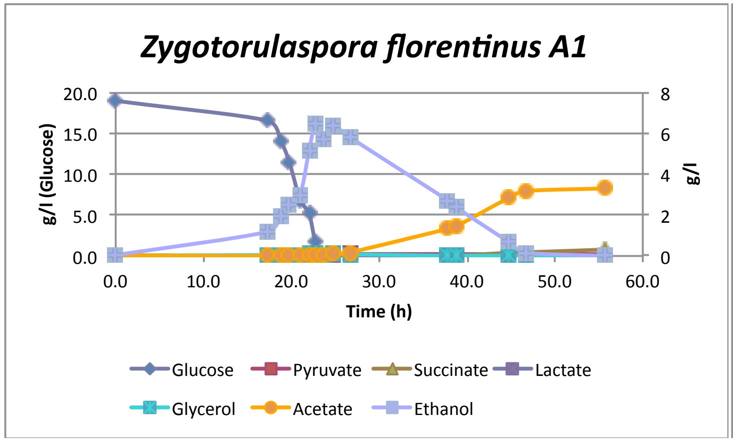
Zygotorulaspora florentinus A1
g/l (Glucose)
g/l
Time (h)
Glucose
Pyruvate
Succinate
Lactate
Glycerol
Acetate
Ethanol

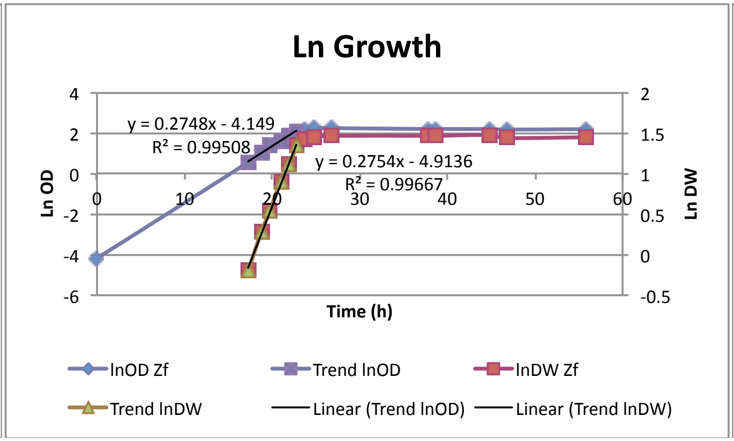
Ln Growth
y = 0.2748x - 4.149
R² = 0.99508
y = 0.2754x - 4.9136
R² = 0.99667
Ln OD
Ln DW
Time (h)
lnOD Zf
Trend lnOD
lnDW Zf
Trend lnDW
Linear (Trend lnOD)
Linear (Trend lnDW)

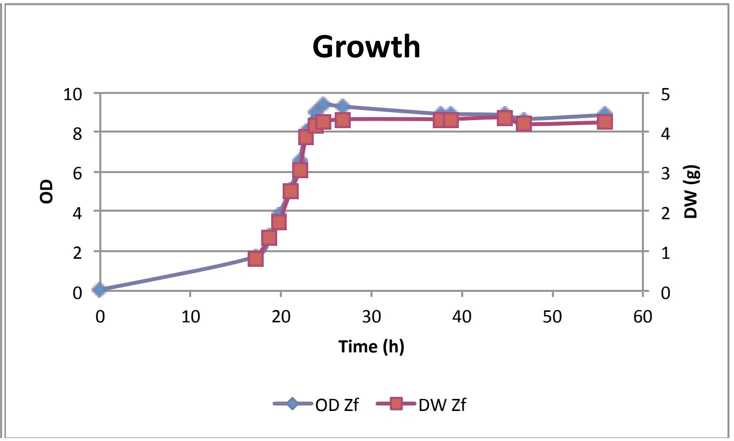
Growth
OD
DW (g)
Time (h)
OD Zf
DW Zf

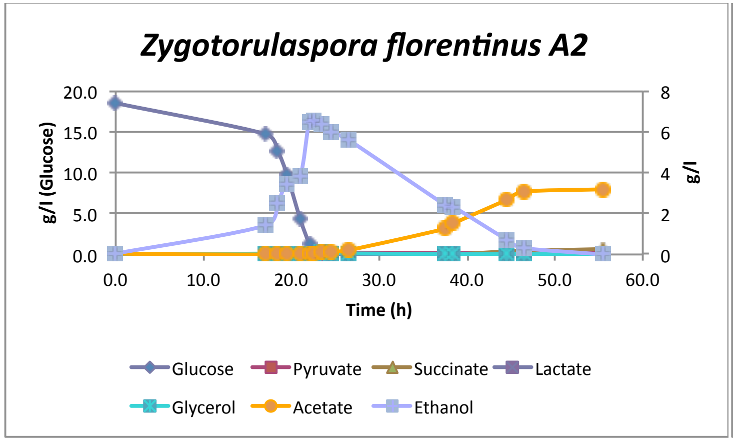
Zygotorulaspora florentinus A2
g/l (Glucose)
g/l
20.0
15.0
10.0
5.0
0.0
8
6
4
2
0
0.0
10.0
20.0
30.0
40.0
50.0
60.0
Time (h)
Glucose
Pyruvate
Succinate
Lactate
Glycerol
Acetate
Ethanol

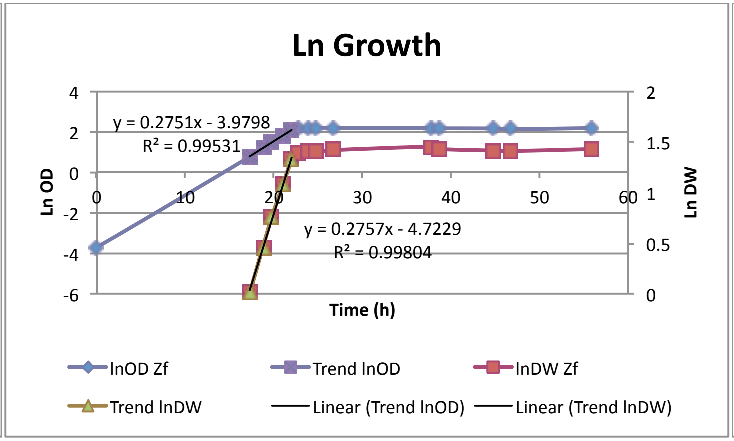
Ln Growth
y = 0.2751x - 3.9798
R² = 0.99531
y = 0.2757x - 4.7229
R² = 0.99804
Ln OD
Ln DW
4
2
0
-2
-4
-6
2
1.5
1
0.5
0
0
10
20
30
40
50
60
Time (h)
lnOD Zf
Trend lnOD
lnDW Zf
Trend lnDW
Linear (Trend lnOD)
Linear (Trend lnDW)

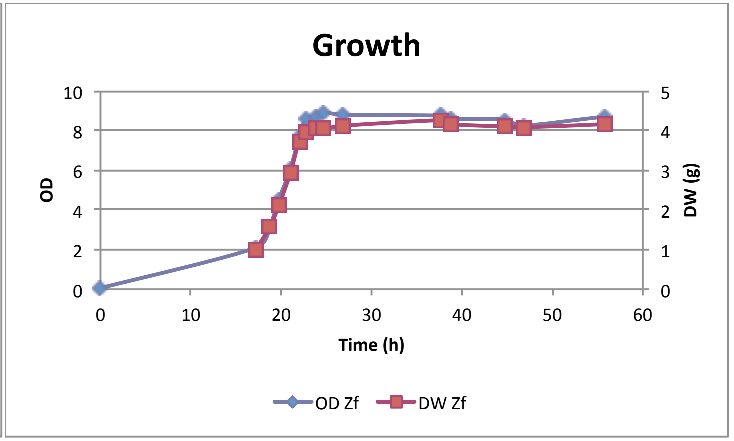
Growth
OD
DW (g)
10
8
6
4
2
0
5
4
3
2
1
0
0
10
20
30
40
50
60
Time (h)
OD Zf
DW Zf

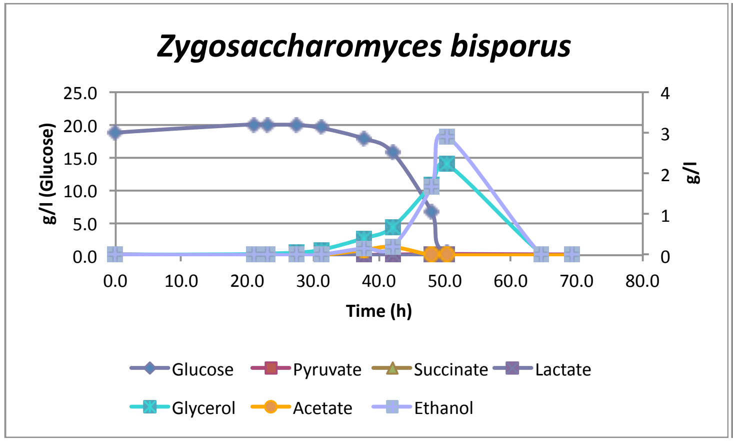
Zygosaccharomyces bisporus
g/l (Glucose)
g/l
25.0
20.0
15.0
10.0
5.0
0.0
4
3
2
1
0
0.0
10.0
20.0
30.0
40.0
50.0
60.0
70.0
80.0
Time (h)
Glucose
Pyruvate
Succinate
Lactate
Glycerol
Acetate
Ethanol

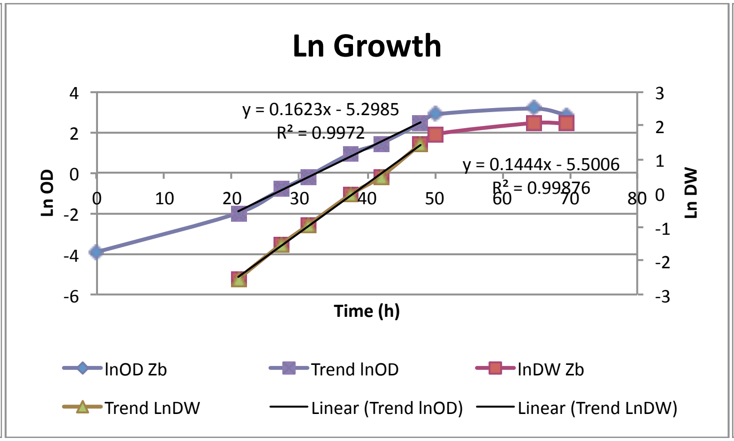
Ln Growth
y = 0.1623x - 5.2985
R² = 0.9972
y = 0.1444x - 5.5006
R² = 0.99876
Ln OD
Ln DW
4
2
0
-2
-4
-6
3
2
1
0
-1
-2
-3
0
10
20
30
40
50
60
70
80
Time (h)
lnOD Zb
Trend lnOD
lnDW Zb
Trend LnDW
Linear (Trend lnOD)
Linear (Trend LnDW)

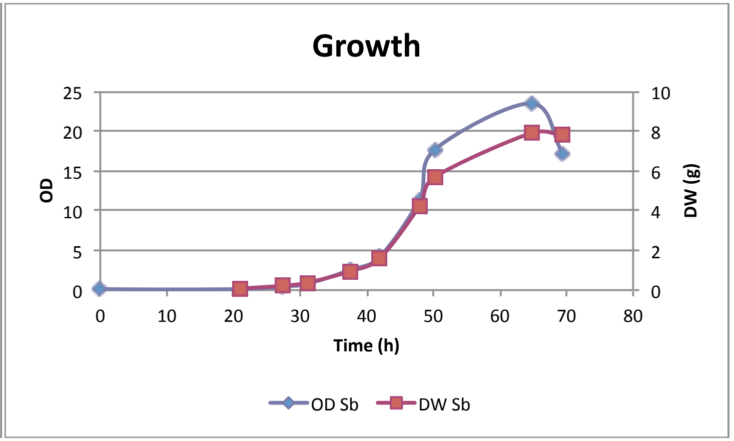
Growth
OD
DW (g)
25
20
15
10
5
0
10
8
6
4
2
0
0
10
20
30
40
50
60
70
80
Time (h)
OD Sb
DW Sb

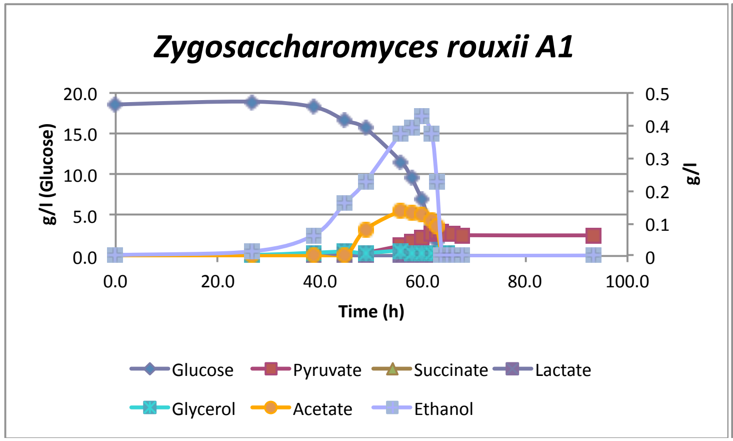
Zygosaccharomyces rouxii A1
g/l (Glucose)
g/l
20.0
15.0
10.0
5.0
0.0
0.5
0.4
0.3
0.2
0.1
0
0.0
20.0
40.0
60.0
80.0
100.0
Time (h)
Glucose
Pyruvate
Succinate
Lactate
Glycerol
Acetate
Ethanol

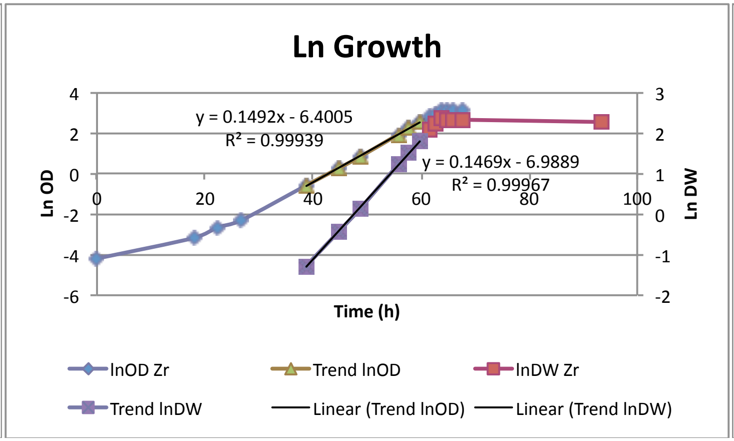
Ln Growth
y = 0.1492x - 6.4005
R² = 0.99939
y = 0.1469x - 6.9889
R² = 0.99967
Ln OD
Ln DW
4
2
0
-2
-4
-6
3
2
1
0
-1
-2
0
20
40
60
80
100
Time (h)
lnOD Zr
Trend lnOD
lnDW Zr
Trend lnDW
Linear (Trend lnOD)
Linear (Trend lnDW)

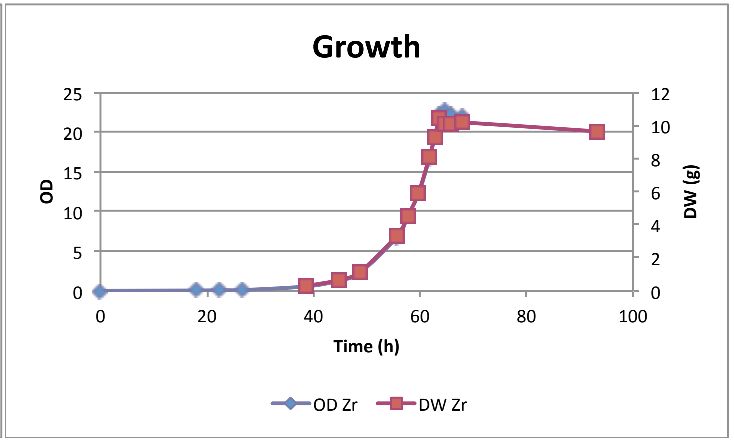
Growth
OD
DW (g)
25
20
15
10
5
0
12
10
8
6
4
2
0
0
20
40
60
80
100
Time (h)
OD Zr
DW Zr

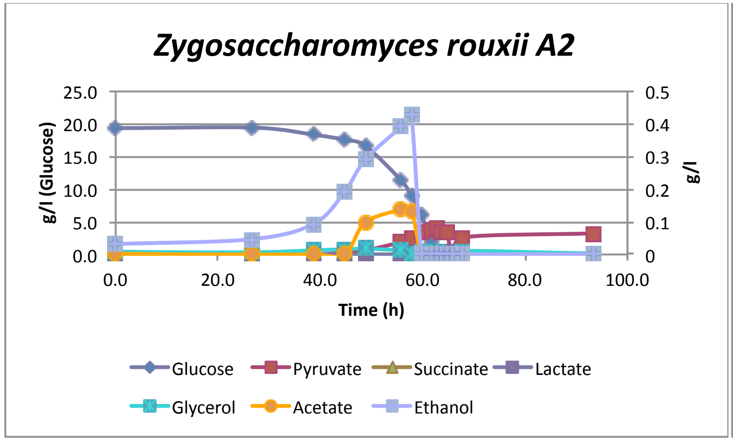
Zygosaccharomyces rouxii A2
g/l (Glucose)
g/l
Time (h)
Glucose
Pyruvate
Succinate
Lactate
Glycerol
Acetate
Ethanol

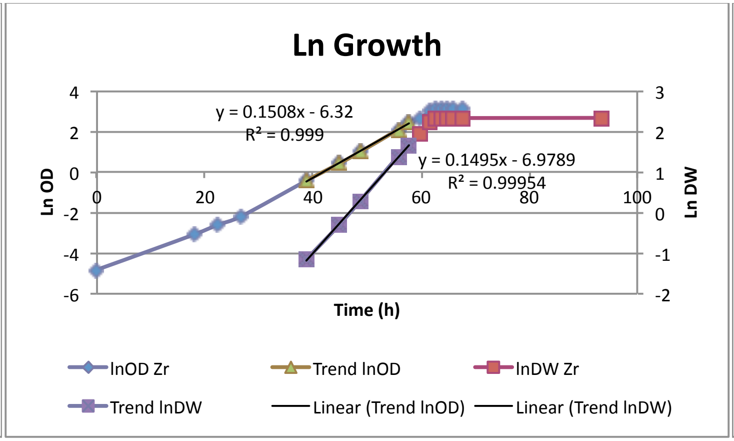
Ln Growth
Ln OD
Ln DW
Time (h)
y = 0.1508x - 6.32
R² = 0.999
y = 0.1495x - 6.9789
R² = 0.99954
lnOD Zr
Trend lnOD
lnDW Zr
Trend lnDW
Linear (Trend lnOD)
Linear (Trend lnDW)

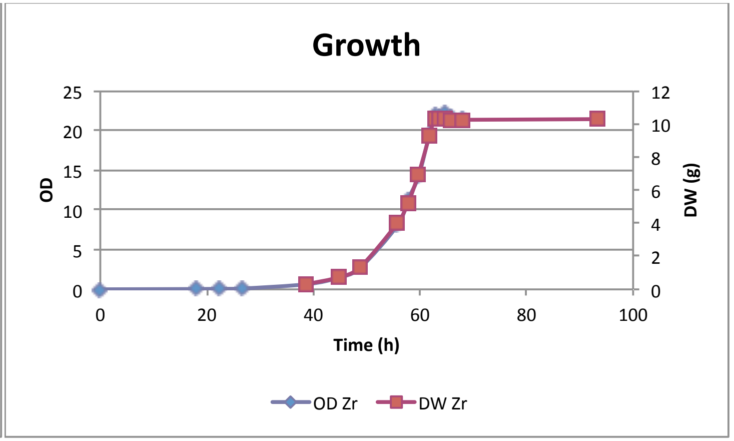
Growth
OD
DW (g)
Time (h)
OD Zr
DW Zr

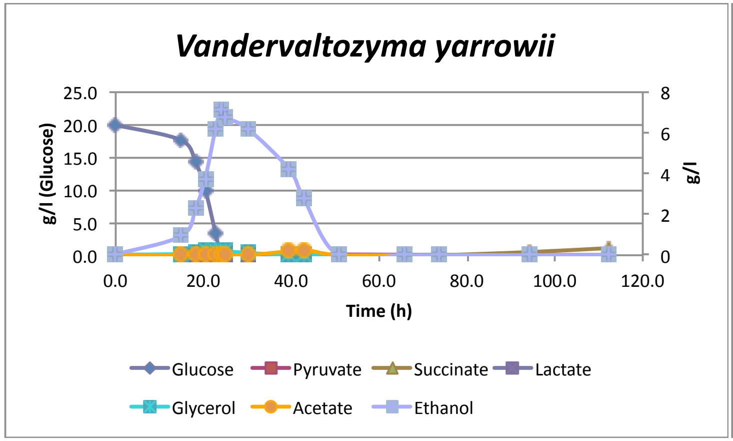
Vandervaltozyma yarrowii
g/l (Glucose)
g/l
Time (h)
Glucose
Pyruvate
Succinate
Lactate
Glycerol
Acetate
Ethanol

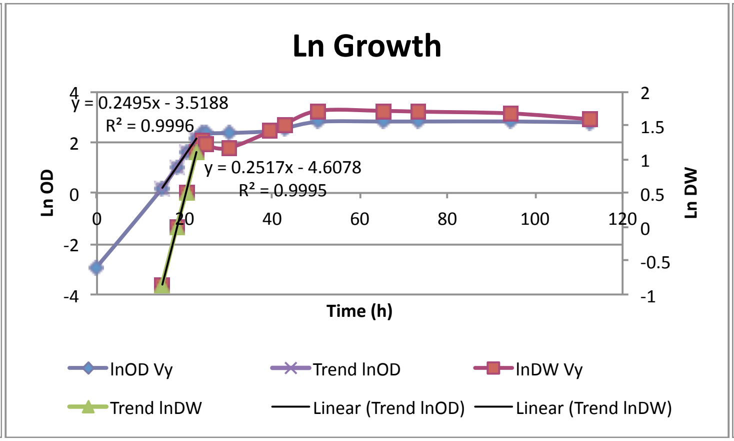
Ln Growth
Ln OD
Ln DW
Time (h)
y = 0.2495x - 3.5188
R² = 0.9996
y = 0.2517x - 4.6078
R² = 0.9995
lnOD Vy
Trend lnOD
lnDW Vy
Trend lnDW
Linear (Trend lnOD)
Linear (Trend lnDW)

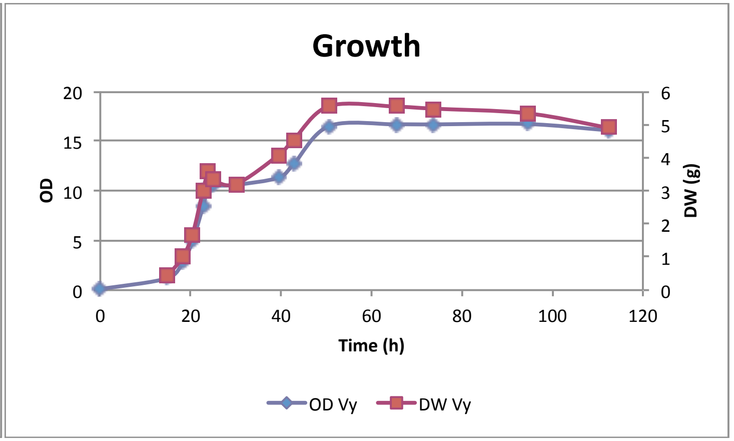
Growth
OD
DW (g)
Time (h)
OD Vy
DW Vy

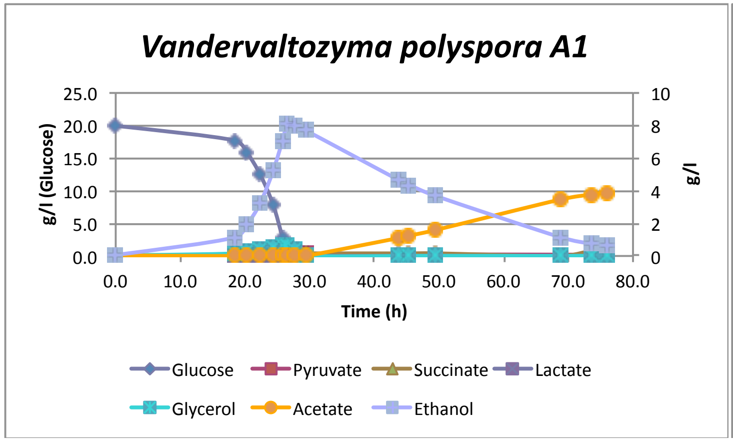
Vandervaltozyma polyspora A1
g/l (Glucose)
g/l
Time (h)
Glucose
Pyruvate
Succinate
Lactate
Glycerol
Acetate
Ethanol

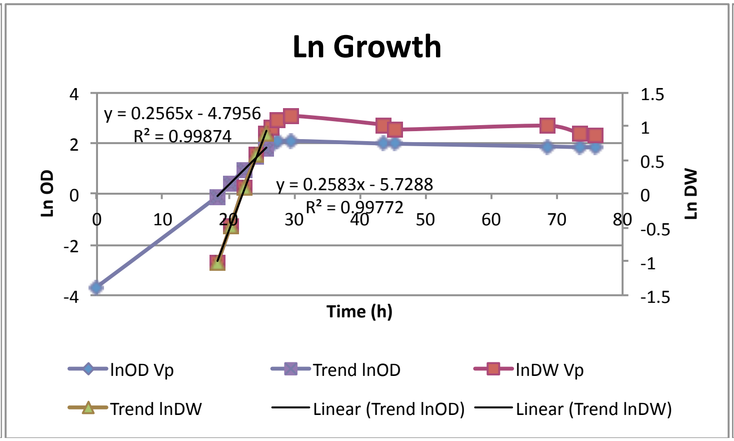
Ln Growth
Ln OD
Ln DW
Time (h)
y = 0.2565x - 4.7956
R² = 0.99874
y = 0.2583x - 5.7288
R² = 0.99772
lnOD Vp
Trend lnOD
lnDW Vp
Trend lnDW
Linear (Trend lnOD)
Linear (Trend lnDW)

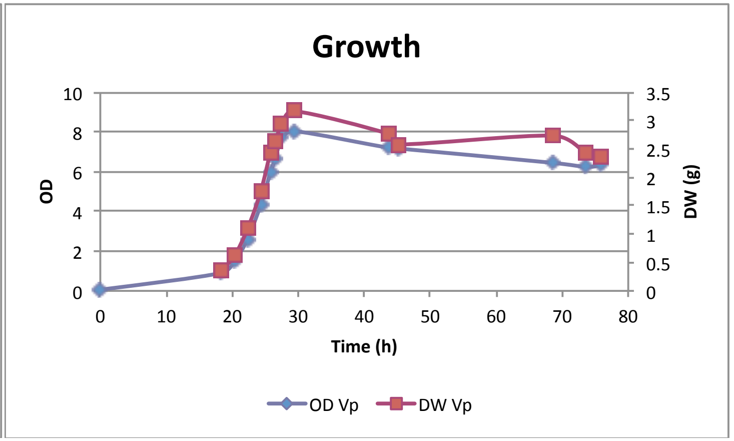
Growth
OD
DW (g)
Time (h)
OD Vp
DW Vp

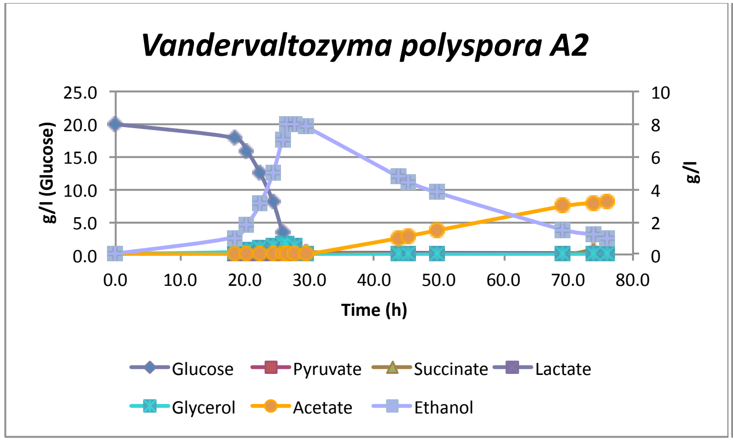
Vandervaltozyma polyspora A2
g/l (Glucose)
g/l
Time (h)
Glucose
Pyruvate
Succinate
Lactate
Glycerol
Acetate
Ethanol

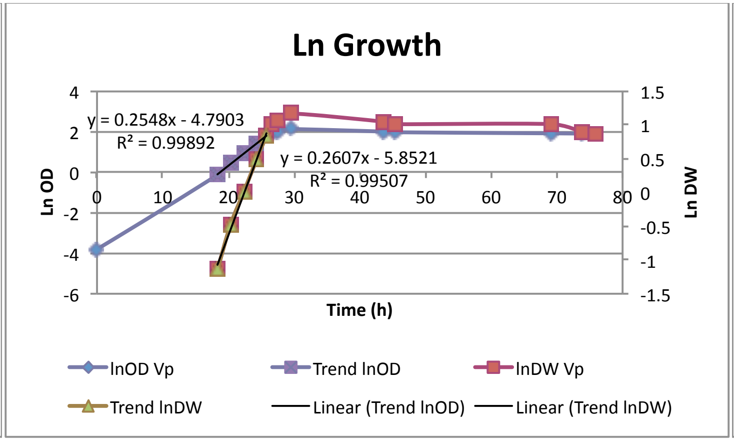
Ln Growth
Ln OD
Ln DW
Time (h)
y = 0.2548x - 4.7903
R² = 0.99892
y = 0.2607x - 5.8521
R² = 0.99507
lnOD Vp
Trend lnOD
lnDW Vp
Trend lnDW
Linear (Trend lnOD)
Linear (Trend lnDW)

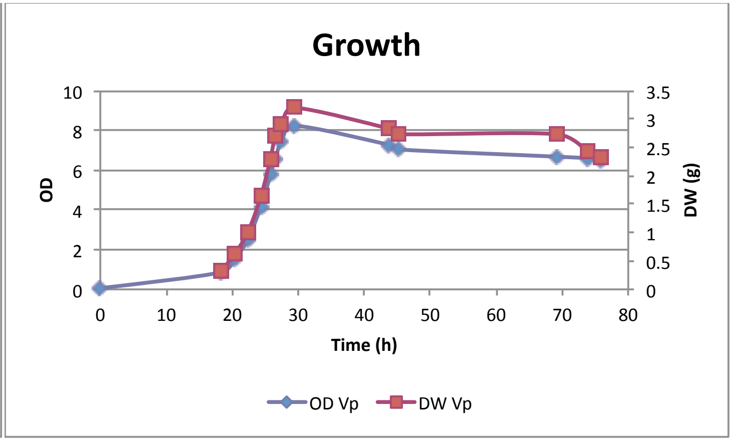
Growth
OD
DW (g)
Time (h)
OD Vp
DW Vp

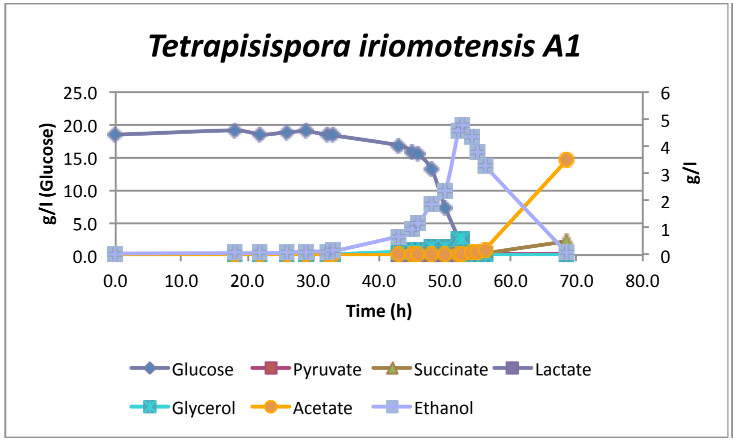
Tetrapisispora iriomotensis A1
g/l (Glucose)
g/l
Time (h)
Glucose
Pyruvate
Succinate
Lactate
Glycerol
Acetate
Ethanol

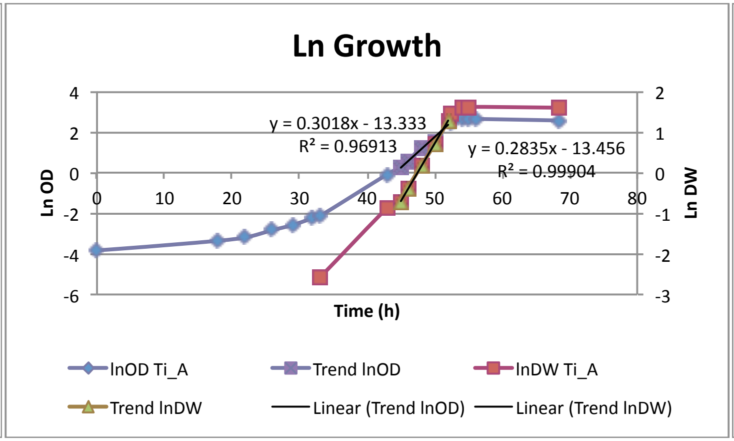
Ln Growth
Ln OD
Ln DW
Time (h)
y = 0.3018x - 13.333
R² = 0.96913
y = 0.2835x - 13.456
R² = 0.99904
lnOD Ti_A
Trend lnOD
lnDW Ti_A
Trend lnDW
Linear (Trend lnOD)
Linear (Trend lnDW)

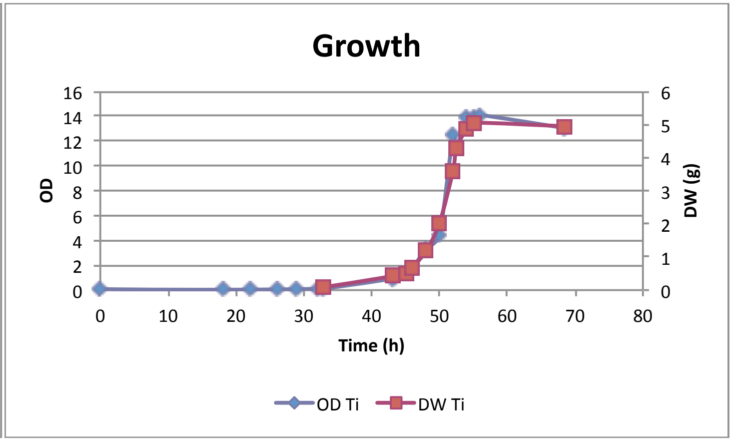
Growth
OD
DW (g)
Time (h)
OD Ti
DW Ti

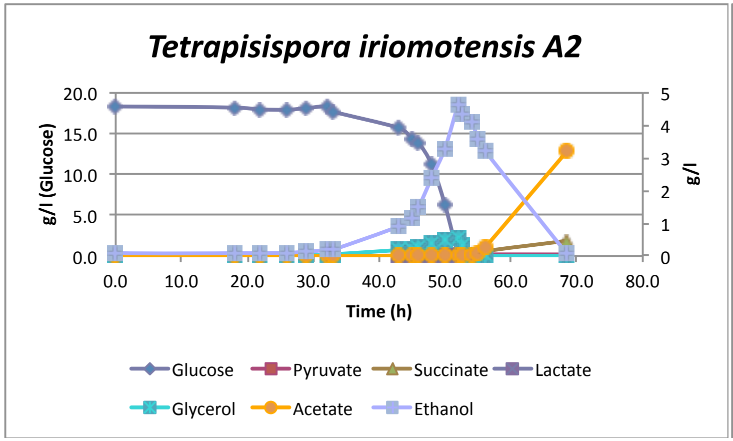
Tetrapisispora iriomotensis A2
g/l (Glucose)
g/l
Time (h)
Glucose
Pyruvate
Succinate
Lactate
Glycerol
Acetate
Ethanol

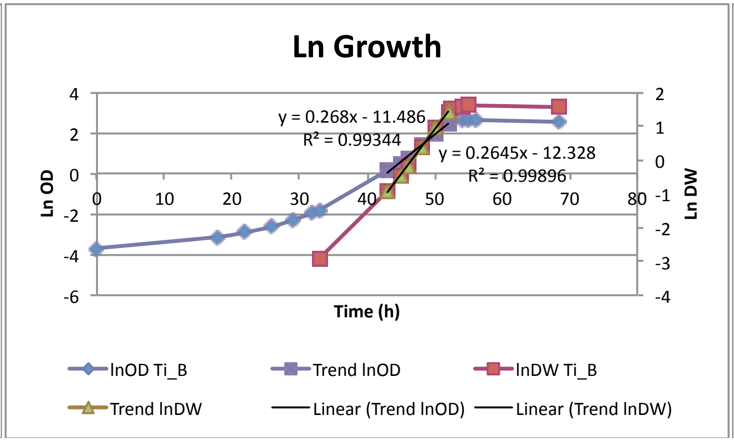
Ln Growth
Ln OD
Ln DW
Time (h)
y = 0.268x - 11.486
R² = 0.99344
y = 0.2645x - 12.328
R² = 0.99896
lnOD Ti_B
Trend lnOD
lnDW Ti_B
Trend lnDW
Linear (Trend lnOD)
Linear (Trend lnDW)

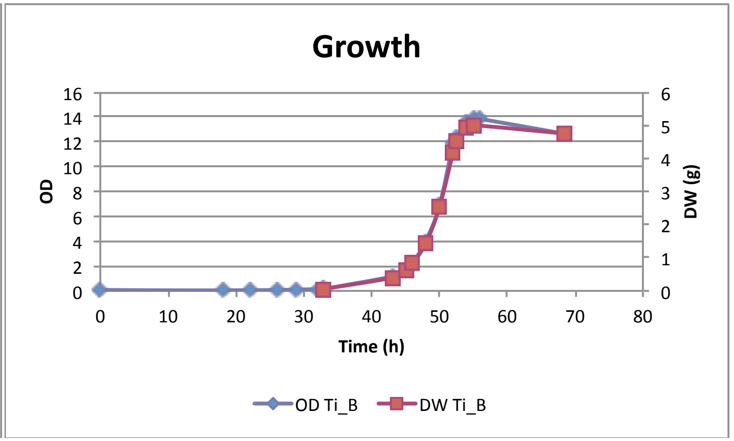
Growth
OD
DW (g)
Time (h)
OD Ti_B
DW Ti_B

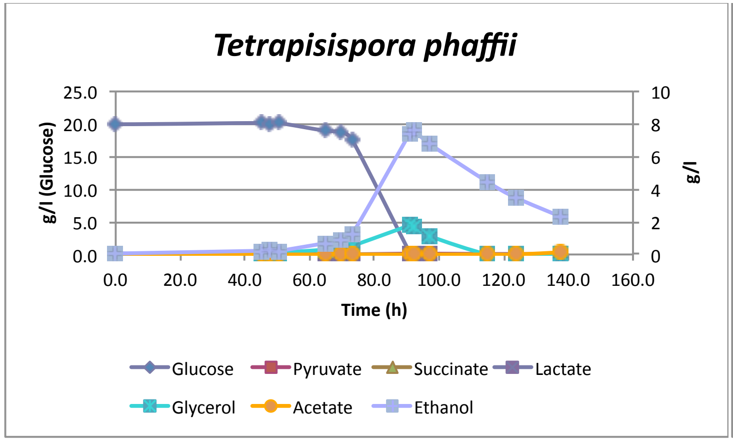
Tetrapisispora phaffii
g/l (Glucose)
g/l
Time (h)
Glucose
Pyruvate
Succinate
Lactate
Glycerol
Acetate
Ethanol

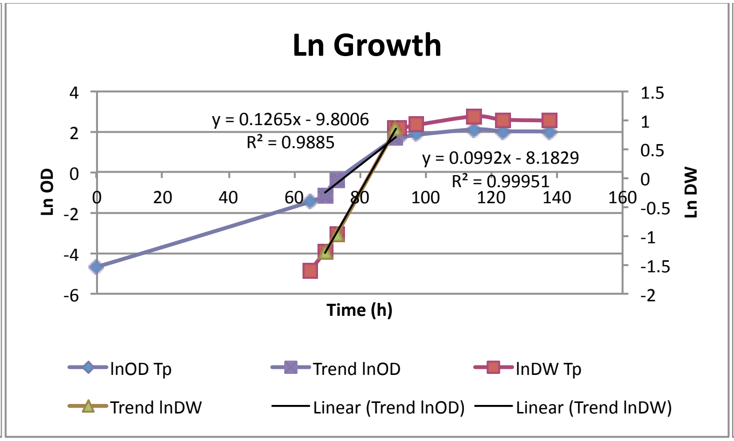
Ln Growth
y = 0.1265x - 9.8006
R² = 0.9885
y = 0.0992x - 8.1829
R² = 0.99951
Ln OD
Ln DW
Time (h)
lnOD Tp
Trend lnOD
lnDW Tp
Trend lnDW
Linear (Trend lnOD)
Linear (Trend lnDW)

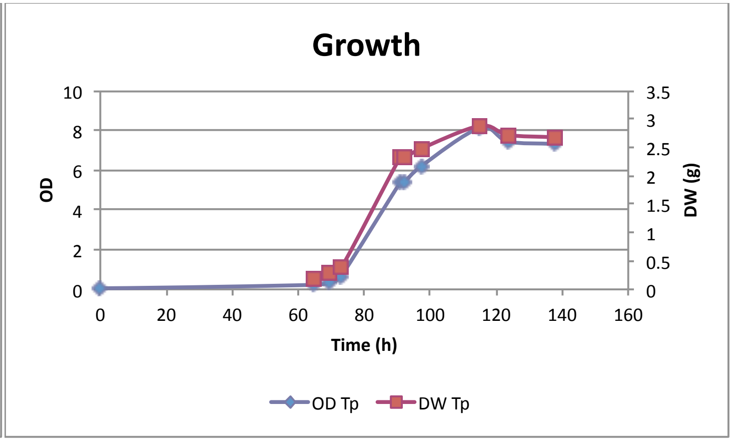
Growth
OD
DW (g)
Time (h)
OD Tp
DW Tp

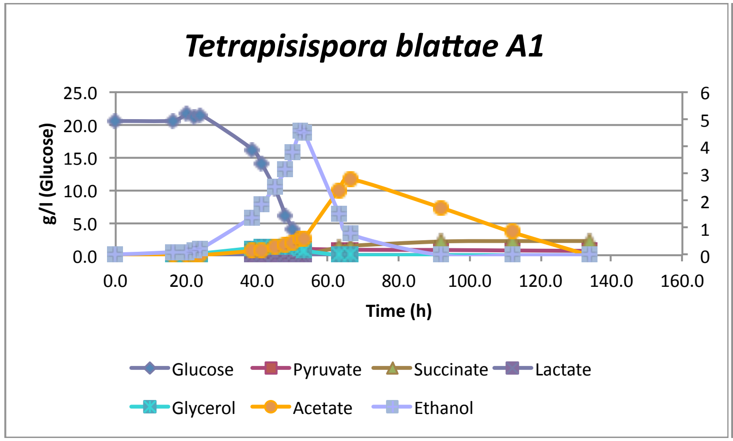
Tetrapisispora blattae A1
g/l (Glucose)
Time (h)
Glucose
Pyruvate
Succinate
Lactate
Glycerol
Acetate
Ethanol

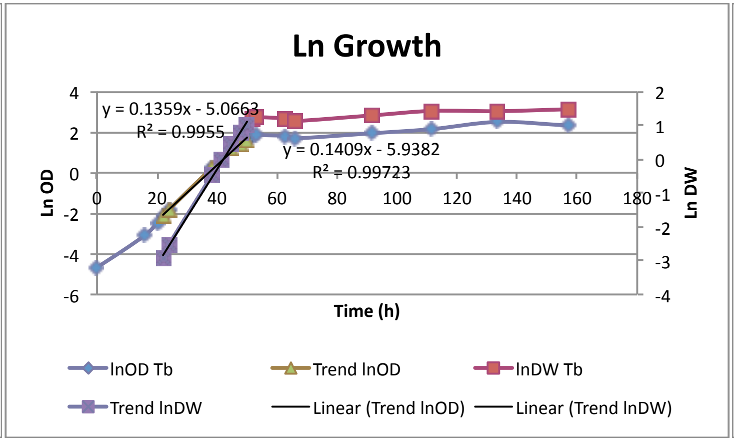
Ln Growth
y = 0.1359x - 5.0663
R² = 0.9955
y = 0.1409x - 5.9382
R² = 0.99723
Ln OD
Ln DW
Time (h)
lnOD Tb
Trend lnOD
lnDW Tb
Trend lnDW
Linear (Trend lnOD)
Linear (Trend lnDW)

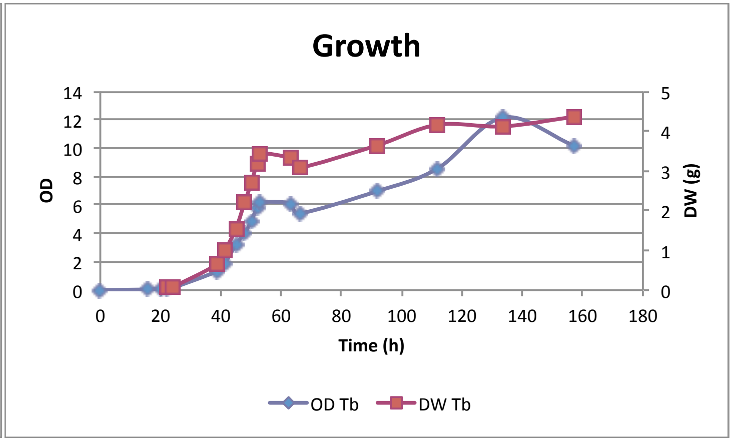
Growth
OD
DW (g)
Time (h)
OD Tb
DW Tb

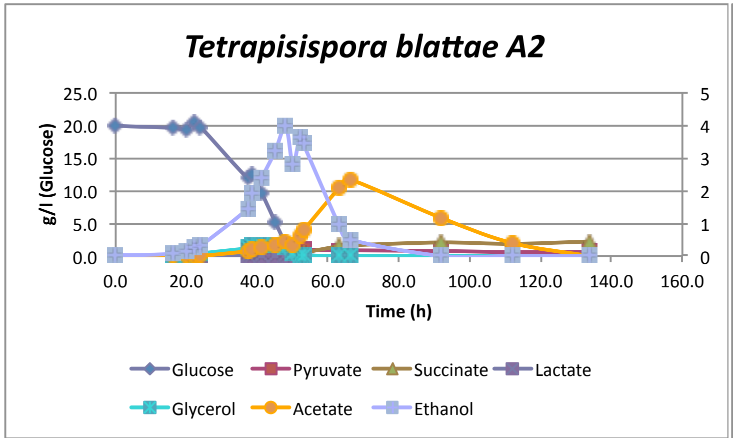
Tetrapisispora blattae A2
g/l (Glucose)
Time (h)
Glucose
Pyruvate
Succinate
Lactate
Glycerol
Acetate
Ethanol

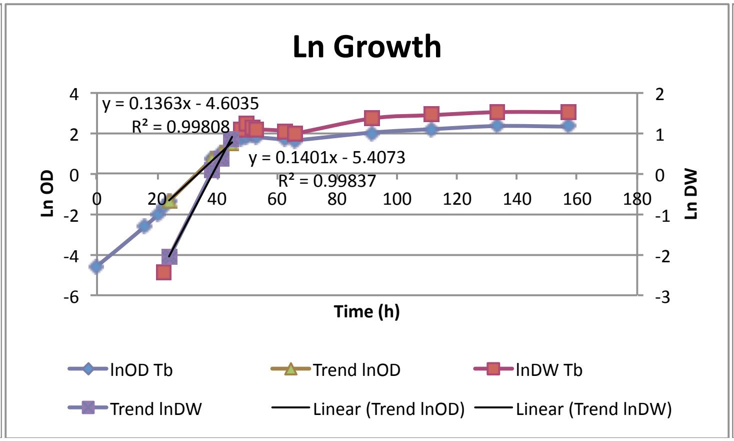
Ln Growth
y = 0.1363x - 4.6035
R² = 0.99808
y = 0.1401x - 5.4073
R² = 0.99837
Ln OD
Ln DW
Time (h)
lnOD Tb
Trend lnOD
lnDW Tb
Trend lnDW
Linear (Trend lnOD)
Linear (Trend lnDW)

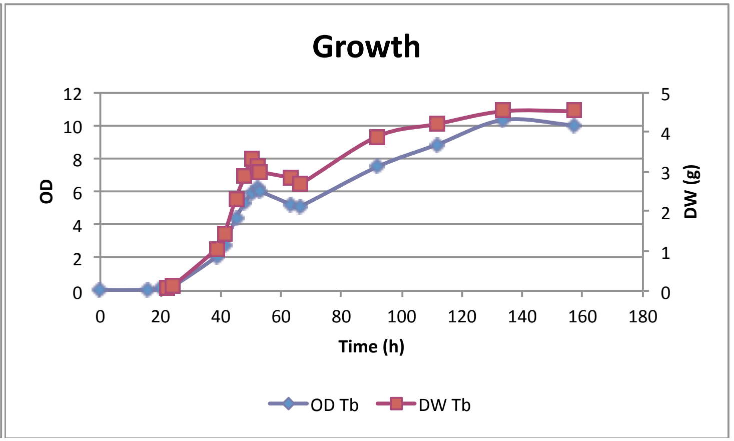
Growth
OD
DW (g)
Time (h)
OD Tb
DW Tb

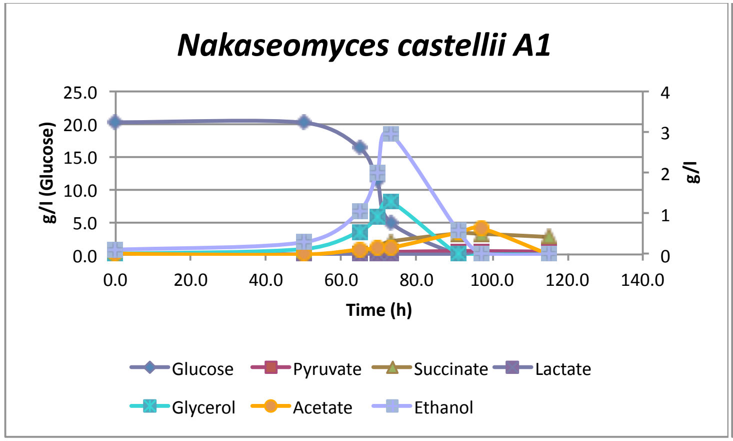
Nakaseomyces castellii A1
g/l (Glucose)
g/l
Time (h)
Glucose
Pyruvate
Succinate
Lactate
Glycerol
Acetate
Ethanol

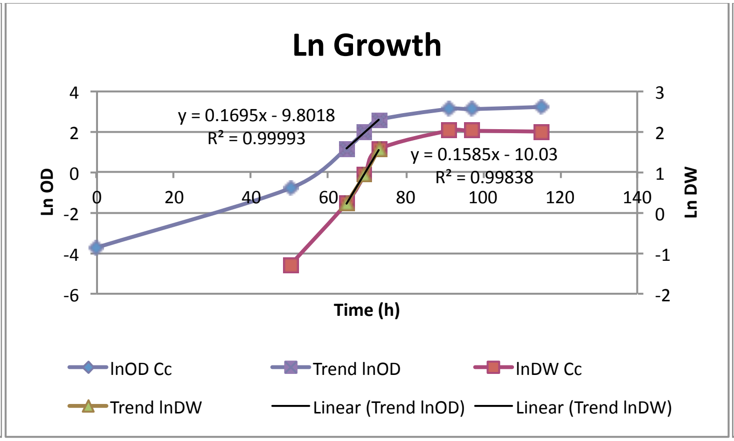
Ln Growth
y = 0.1695x - 9.8018
R² = 0.99993
y = 0.1585x - 10.03
R² = 0.99838
Ln OD
Ln DW
Time (h)
lnOD Cc
Trend lnOD
lnDW Cc
Trend lnDW
Linear (Trend lnOD)
Linear (Trend lnDW)

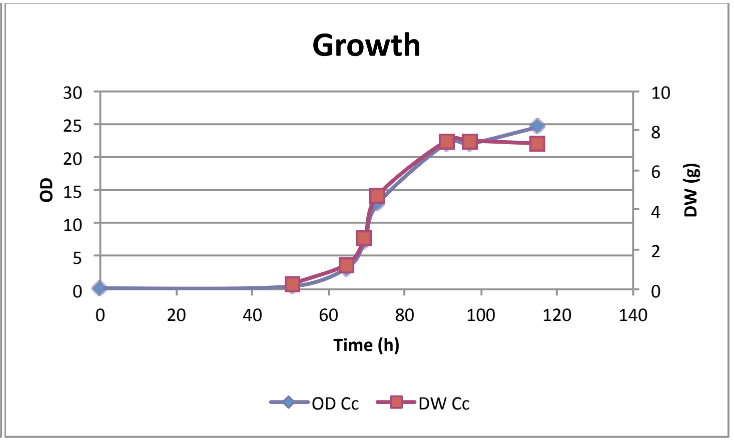
Growth
OD
DW (g)
Time (h)
OD Cc
DW Cc

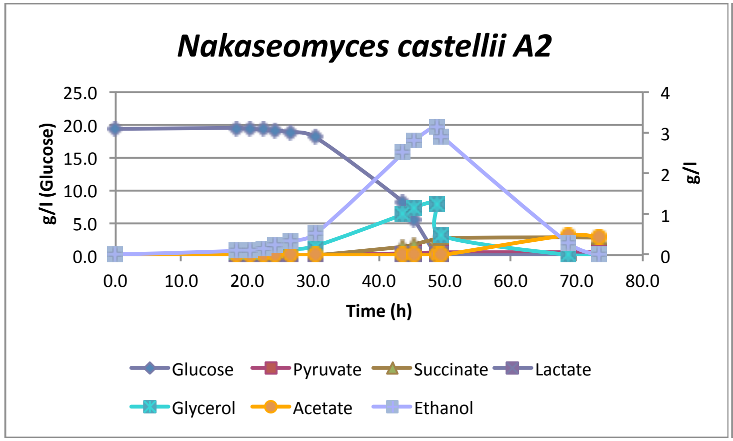
Nakaseomyces castellii A2
g/l (Glucose)
g/l
Time (h)
Glucose
Pyruvate
Succinate
Lactate
Glycerol
Acetate
Ethanol

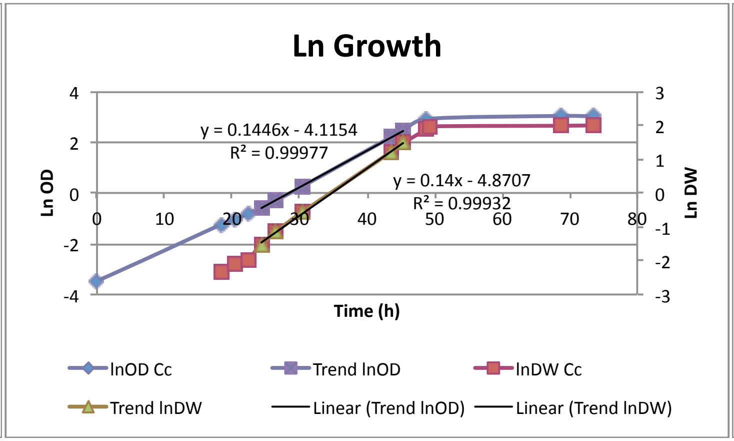
Ln Growth
y = 0.1446x - 4.1154
R² = 0.99977
y = 0.14x - 4.8707
R² = 0.99932
Ln OD
Ln DW
Time (h)
lnOD Cc
Trend lnOD
lnDW Cc
Trend lnDW
Linear (Trend lnOD)
Linear (Trend lnDW)

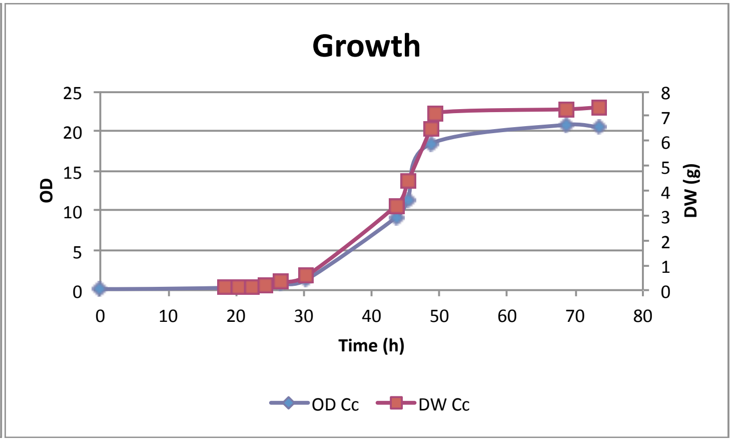
Growth
OD
DW (g)
Time (h)
OD Cc
DW Cc

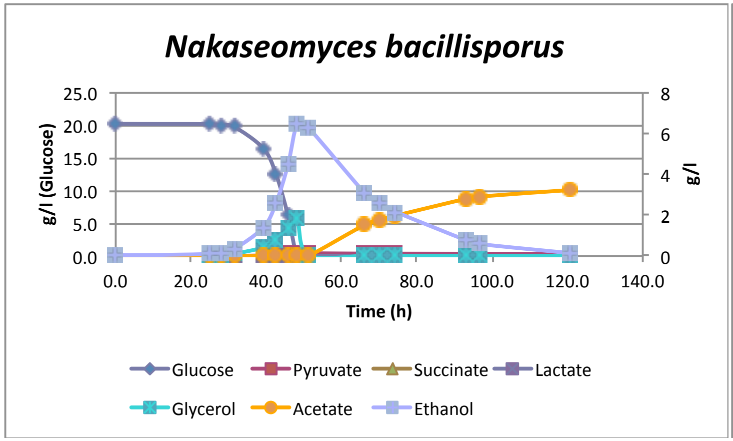
Nakaseomyces bacillisporus
g/l (Glucose)
g/l
Time (h)
Glucose
Pyruvate
Succinate
Lactate
Glycerol
Acetate
Ethanol

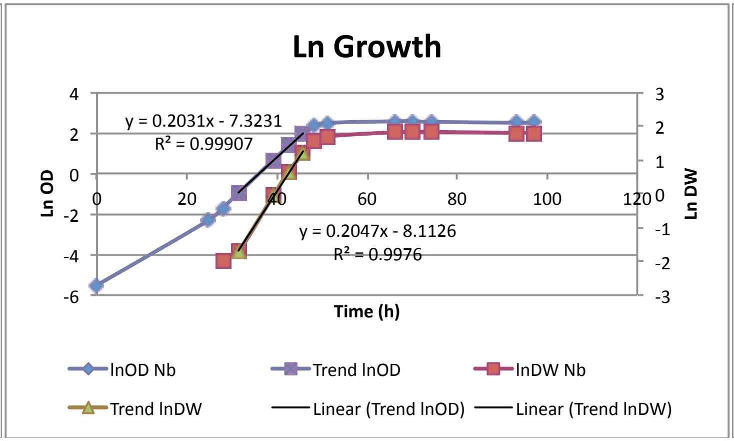
Ln Growth
y = 0.2031x - 7.3231
R² = 0.99907
y = 0.2047x - 8.1126
R² = 0.9976
Ln OD
Ln DW
Time (h)
lnOD Nb
Trend lnOD
lnDW Nb
Trend lnDW
Linear (Trend lnOD)
Linear (Trend lnDW)

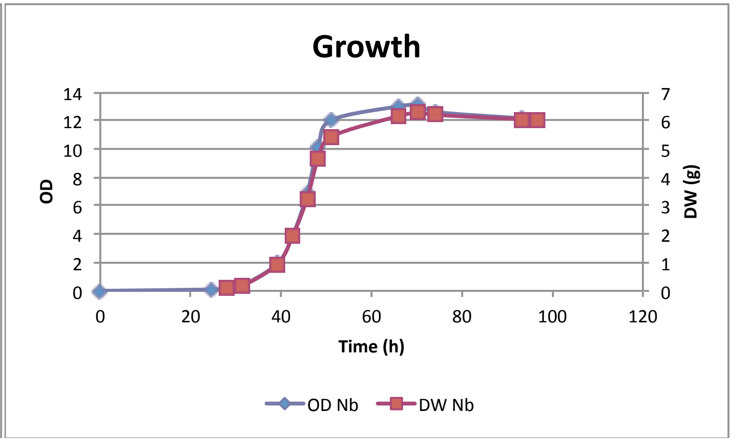
Growth
OD
DW (g)
Time (h)
OD Nb
DW Nb

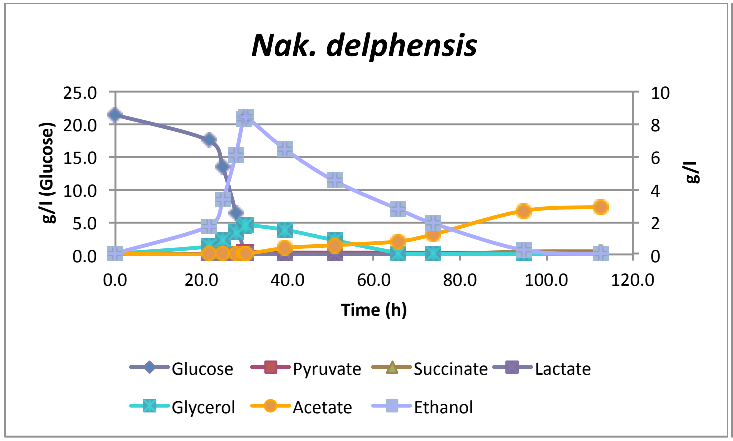
Nak. delphensis
g/l (Glucose)
g/l
Time (h)
Glucose
Pyruvate
Succinate
Lactate
Glycerol
Acetate
Ethanol

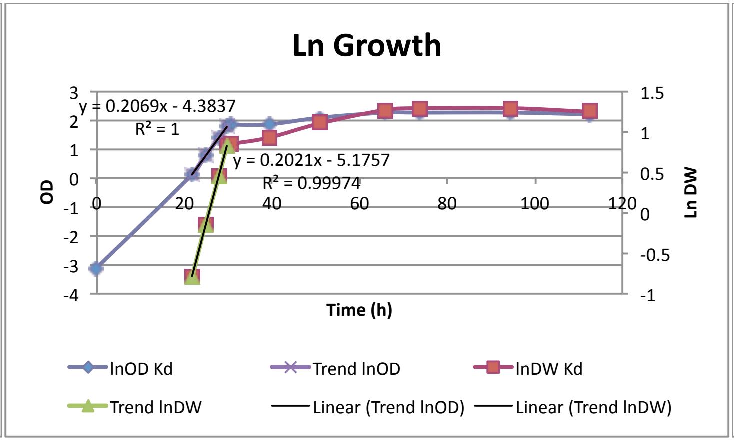
Ln Growth
y = 0.2069x - 4.3837
R² = 1
y = 0.2021x - 5.1757
R² = 0.99974
OD
Ln DW
Time (h)
lnOD Kd
Trend lnOD
lnDW Kd
Trend lnDW
Linear (Trend lnOD)
Linear (Trend lnDW)

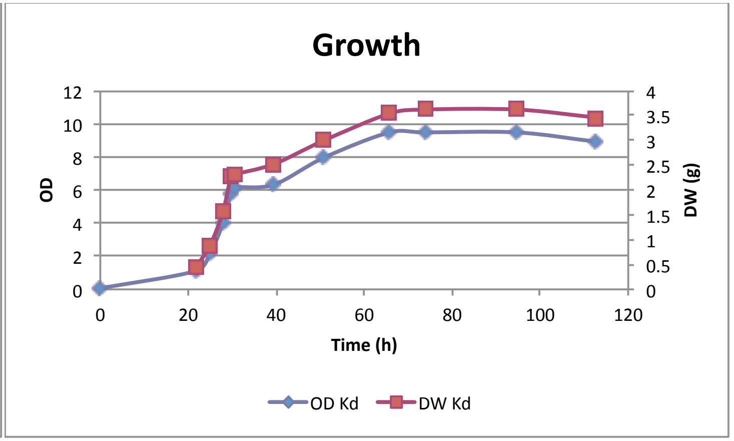
Growth
OD
DW (g)
Time (h)
OD Kd
DW Kd

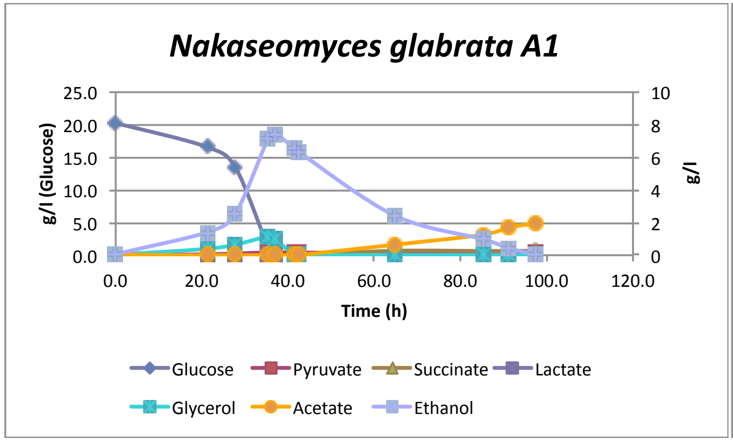
Nakaseomyces glabrata A1
g/l (Glucose)
g/l
Time (h)
Glucose
Pyruvate
Succinate
Lactate
Glycerol
Acetate
Ethanol

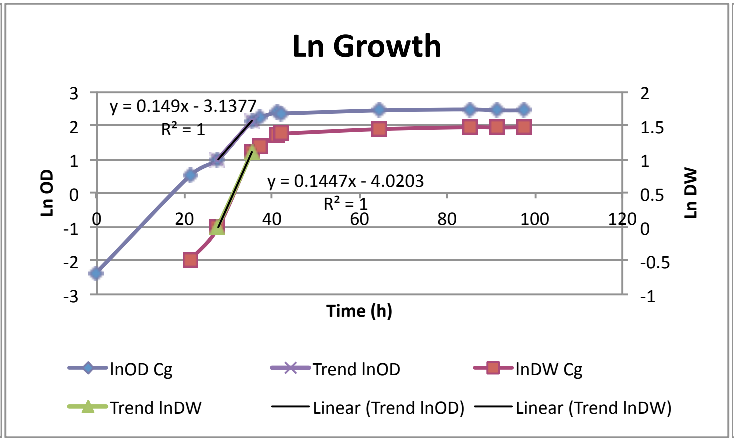
Ln Growth
y = 0.149x - 3.1377
R² = 1
y = 0.1447x - 4.0203
R² = 1
Ln OD
Ln DW
Time (h)
lnOD Cg
Trend lnOD
lnDW Cg
Trend lnDW
Linear (Trend lnOD)
Linear (Trend lnDW)

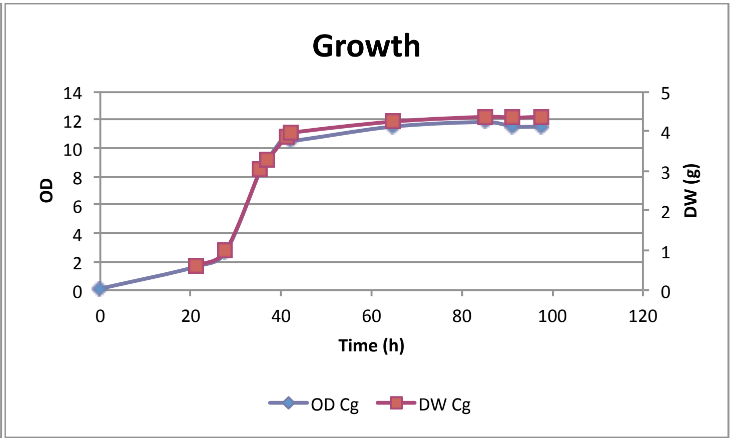
Growth
OD
DW (g)
Time (h)
OD Cg
DW Cg

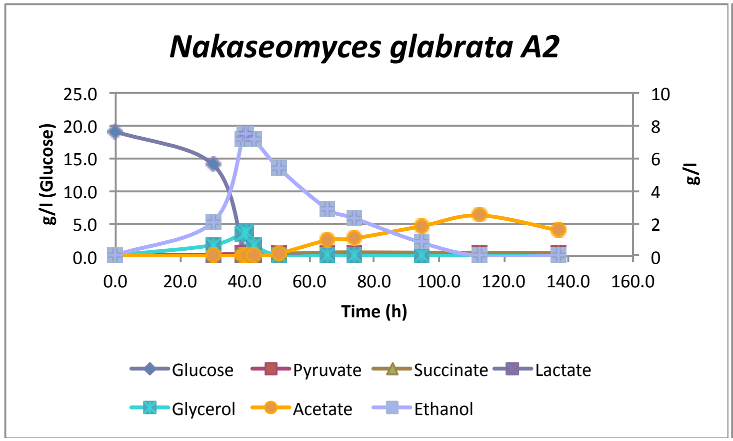
Nakaseomyces glabrata A2
g/l (Glucose)
g/l
Time (h)
Glucose
Pyruvate
Succinate
Lactate
Glycerol
Acetate
Ethanol

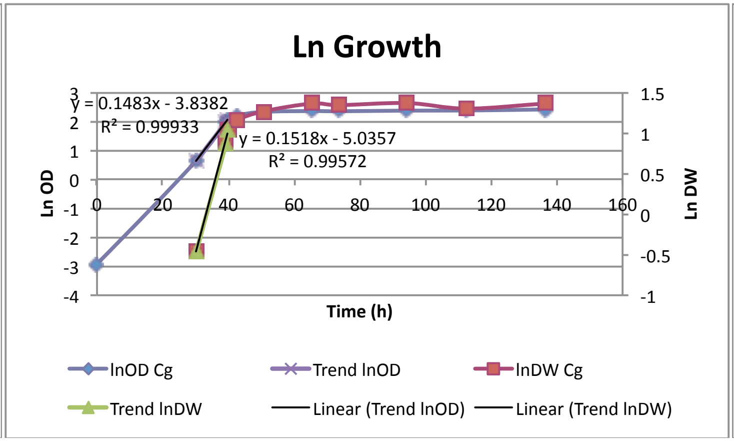
Ln Growth
y = 0.1483x - 3.8382
R² = 0.99933
y = 0.1518x - 5.0357
R² = 0.99572
Ln OD
Ln DW
Time (h)
lnOD Cg
Trend lnOD
lnDW Cg
Trend lnDW
Linear (Trend lnOD)
Linear (Trend lnDW)

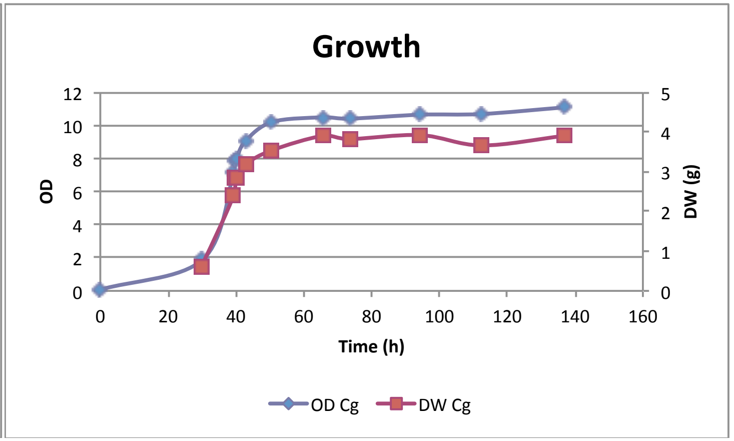
Growth
OD
DW (g)
Time (h)
OD Cg
DW Cg

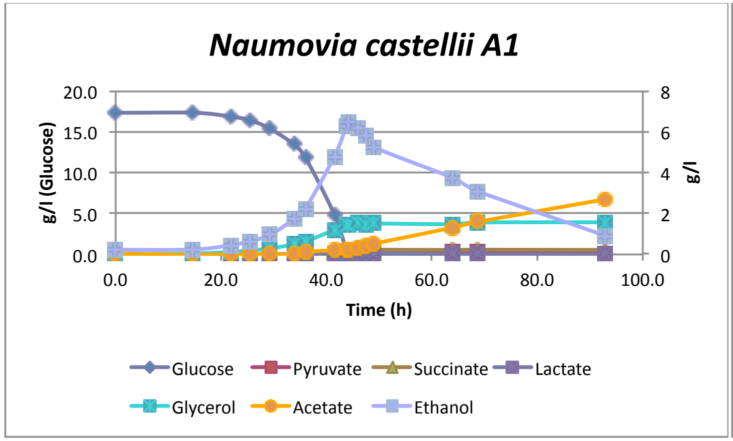
Naumovia castellii A1
g/l (Glucose)
g/l
Time (h)
Glucose
Pyruvate
Succinate
Lactate
Glycerol
Acetate
Ethanol

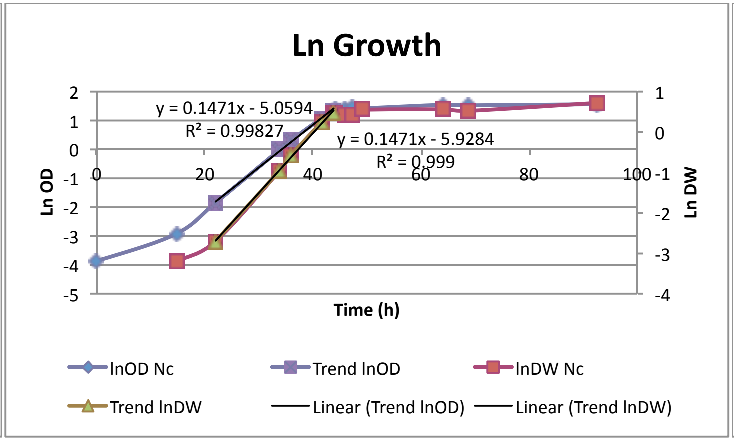
Ln Growth
y = 0.1471x - 5.0594
R² = 0.99827
y = 0.1471x - 5.9284
R² = 0.999
Ln OD
Ln DW
Time (h)
lnOD Nc
Trend lnOD
lnDW Nc
Trend lnDW
Linear (Trend lnOD)
Linear (Trend lnDW)

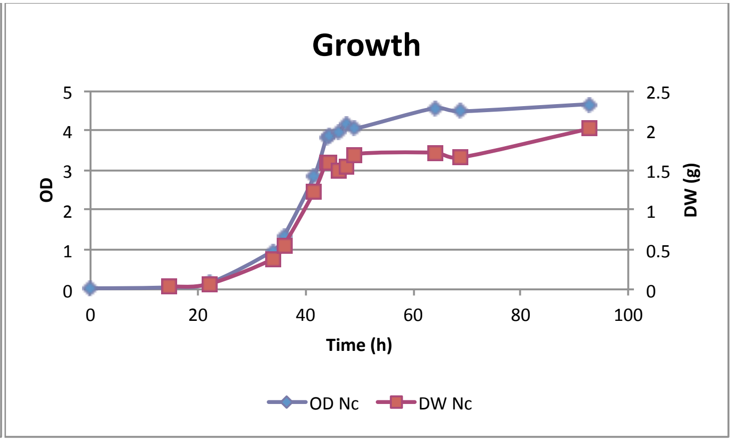
Growth
OD
DW (g)
Time (h)
OD Nc
DW Nc

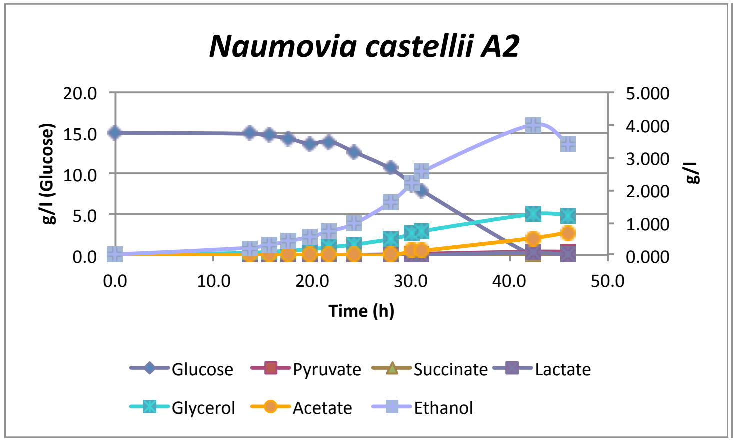
Naumovia castellii A2
g/l (Glucose)
g/l
Time (h)
Glucose
Pyruvate
Succinate
Lactate
Glycerol
Acetate
Ethanol

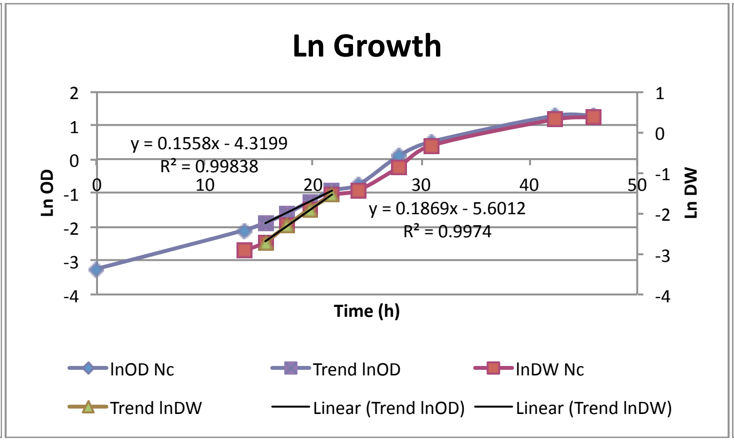
Ln Growth
y = 0.1558x - 4.3199
R² = 0.99838
y = 0.1869x - 5.6012
R² = 0.9974
Ln OD
Ln DW
Time (h)
lnOD Nc
Trend lnOD
lnDW Nc
Trend lnDW
Linear (Trend lnOD)
Linear (Trend lnDW)

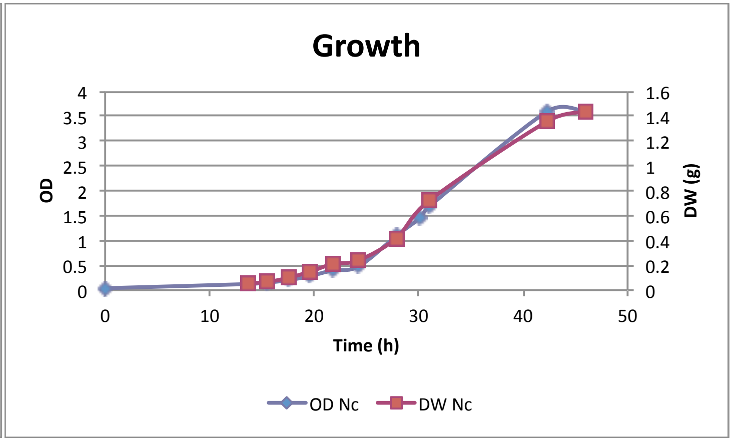
Growth
OD
DW (g)
Time (h)
OD Nc
DW Nc

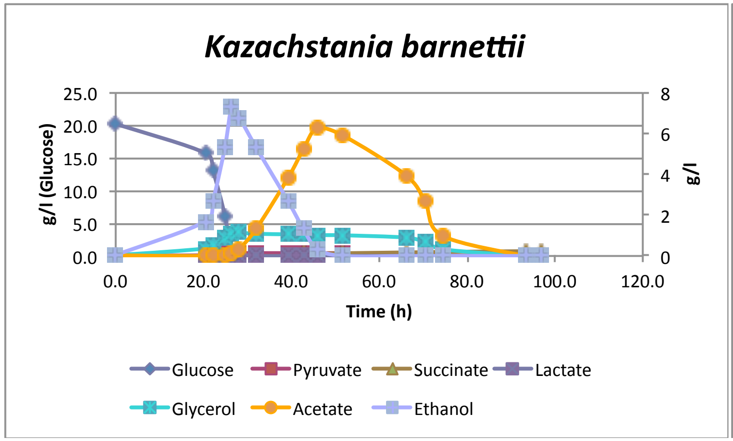
Kazachstania barnettii
g/l (Glucose)
g/l
Time (h)
Glucose
Pyruvate
Succinate
Lactate
Glycerol
Acetate
Ethanol

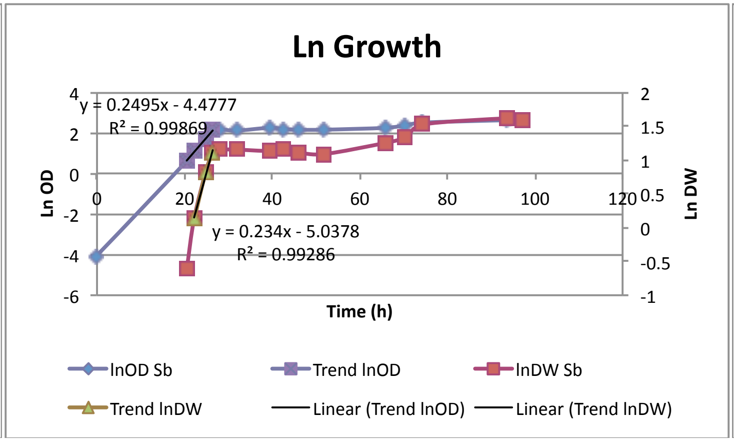
Ln Growth
y = 0.2495x - 4.4777
R² = 0.99869
y = 0.234x - 5.0378
R² = 0.99286
Ln OD
Ln DW
Time (h)
lnOD Sb
Trend lnOD
lnDW Sb
Trend lnDW
Linear (Trend lnOD)
Linear (Trend lnDW)

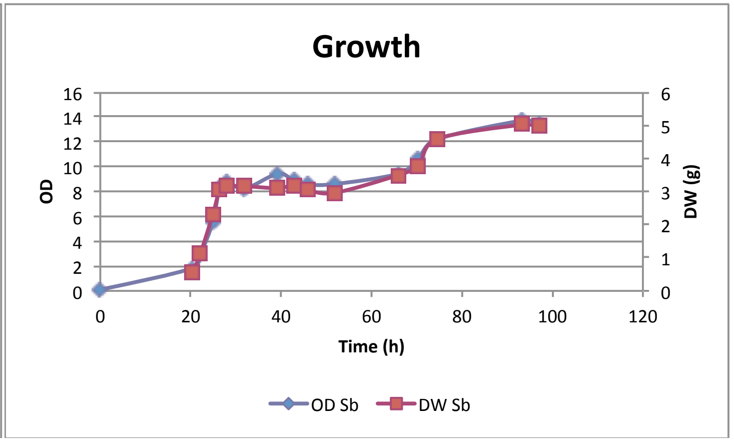
Growth
OD
DW (g)
Time (h)
OD Sb
DW Sb

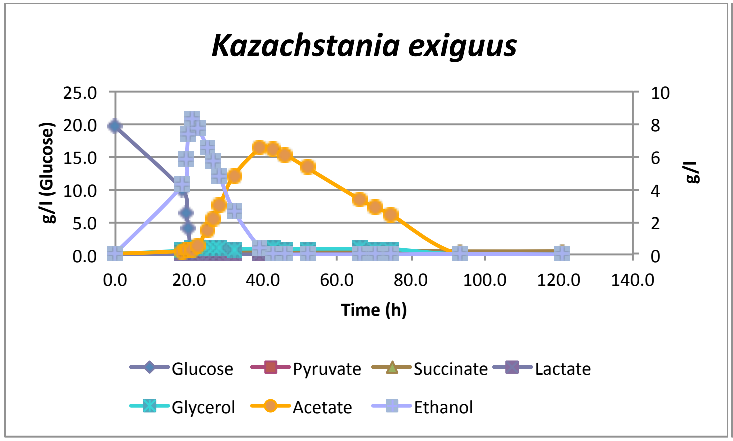
Kazachstania exiguus
g/l (Glucose)
g/l
Time (h)
Glucose
Pyruvate
Succinate
Lactate
Glycerol
Acetate
Ethanol

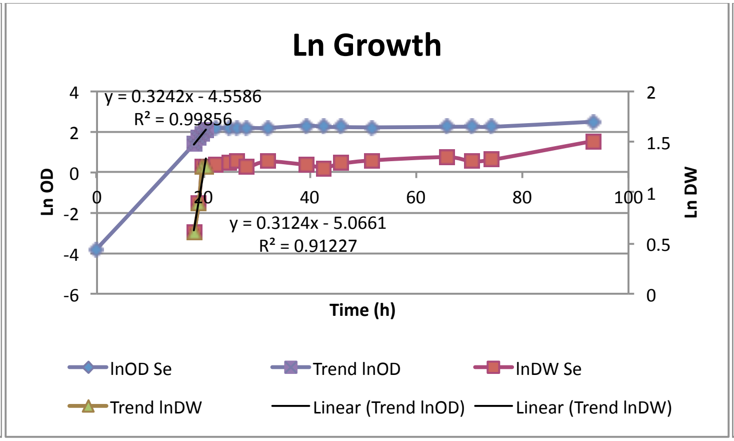
Ln Growth
y = 0.3242x - 4.5586
R² = 0.99856
y = 0.3124x - 5.0661
R² = 0.91227
Ln OD
Ln DW
Time (h)
lnOD Se
Trend lnOD
lnDW Se
Trend lnDW
Linear (Trend lnOD)
Linear (Trend lnDW)

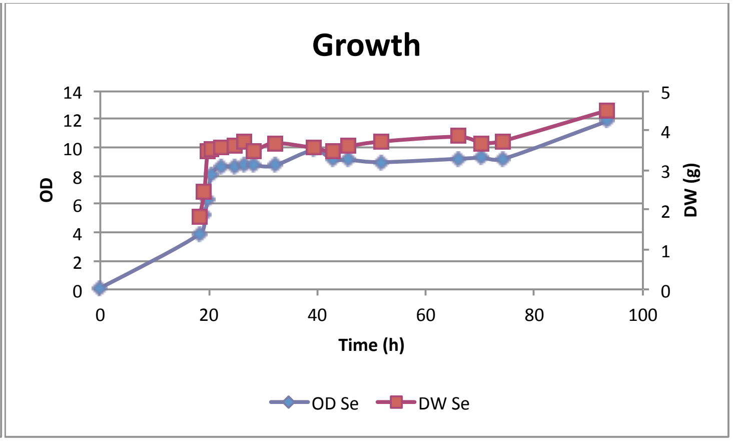
Growth
OD
DW (g)
Time (h)
OD Se
DW Se

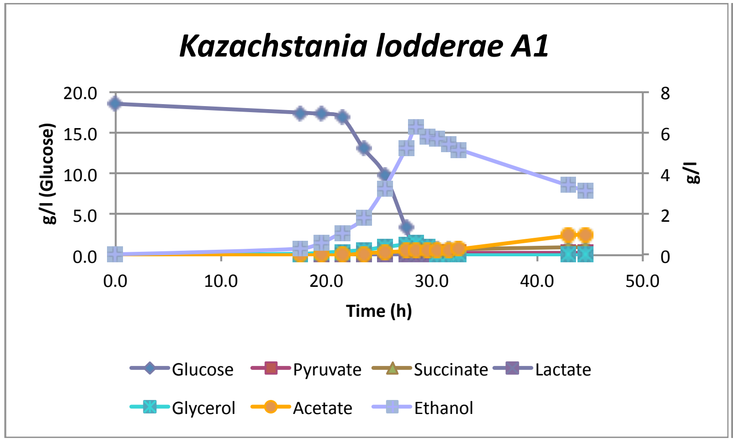
Kazachstania lodderae A1
g/l (Glucose)
g/l
Time (h)
Glucose
Pyruvate
Succinate
Lactate
Glycerol
Acetate
Ethanol

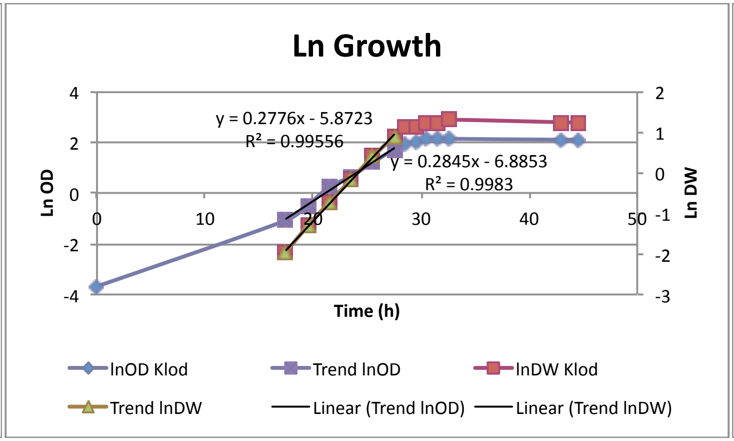
Ln Growth
y = 0.2776x - 5.8723
R² = 0.99556
y = 0.2845x - 6.8853
R² = 0.9983
Ln OD
Ln DW
Time (h)
lnOD Klod
Trend lnOD
lnDW Klod
Trend lnDW
Linear (Trend lnOD)
Linear (Trend lnDW)

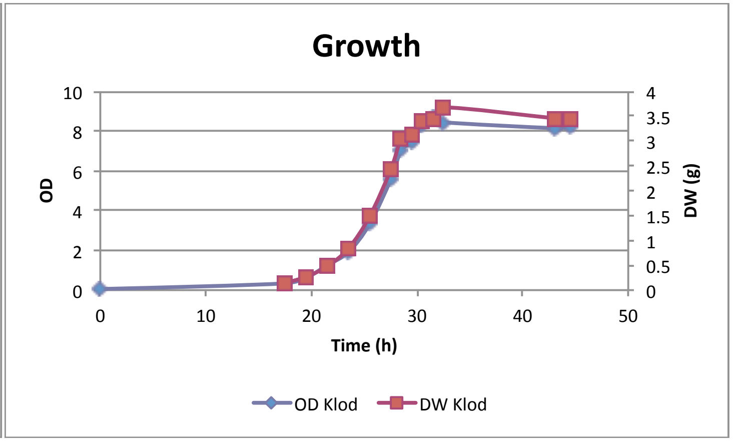
Growth
OD
DW (g)
Time (h)
OD Klod
DW Klod

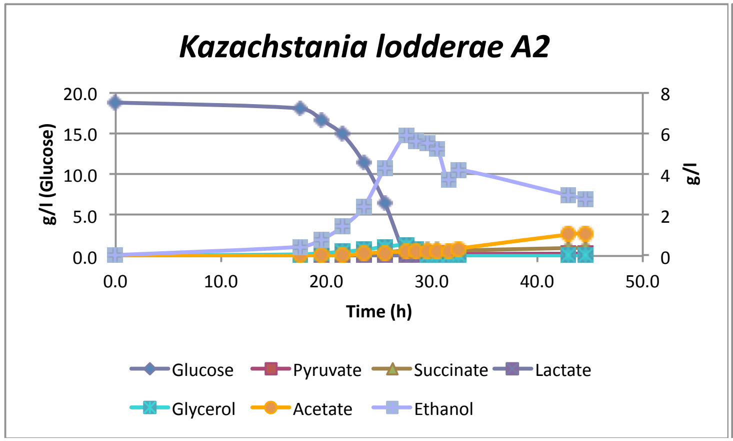
Kazachstania lodderae A2
g/l (Glucose)
g/l
Time (h)
Glucose
Pyruvate
Succinate
Lactate
Glycerol
Acetate
Ethanol

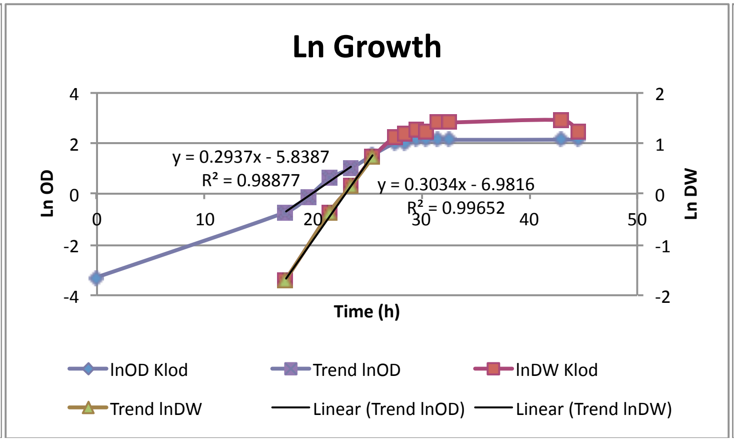
Ln Growth
y = 0.2937x - 5.8387
R² = 0.98877
y = 0.3034x - 6.9816
R² = 0.99652
Ln OD
Ln DW
Time (h)
lnOD Klod
Trend lnOD
lnDW Klod
Trend lnDW
Linear (Trend lnOD)
Linear (Trend lnDW)

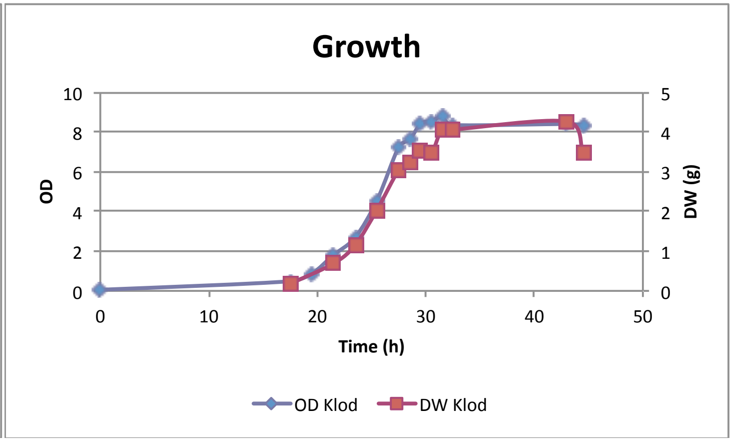
Growth
OD
DW (g)
Time (h)
OD Klod
DW Klod

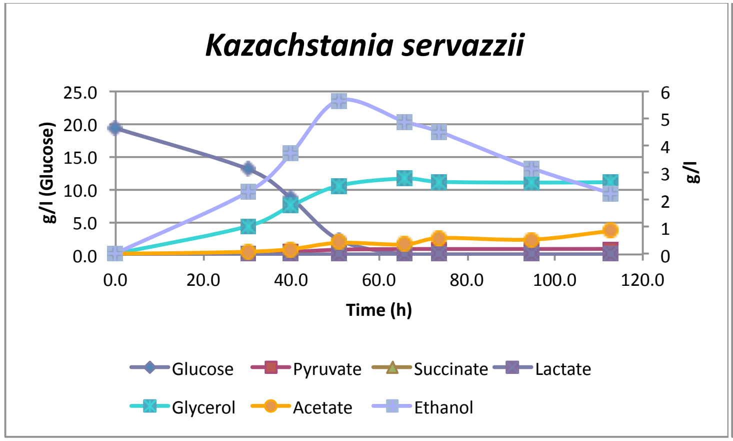
Kazachstania servazzii
g/l (Glucose)
g/l
Time (h)
Glucose
Pyruvate
Succinate
Lactate
Glycerol
Acetate
Ethanol

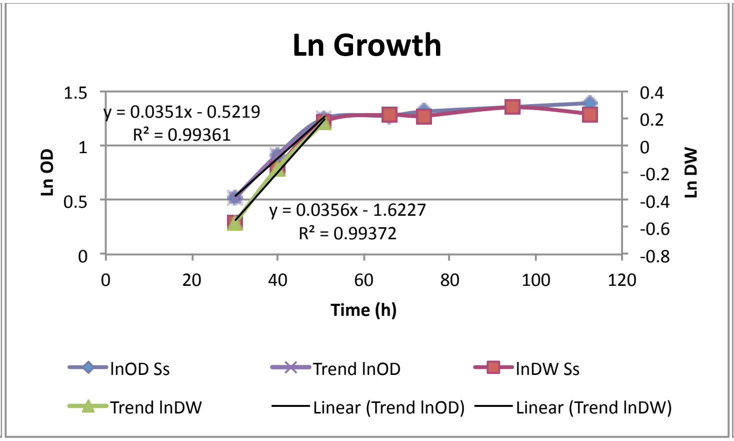
Ln Growth
y = 0.0351x - 0.5219
R² = 0.99361
y = 0.0356x - 1.6227
R² = 0.99372
Ln OD
Ln DW
Time (h)
lnOD Ss
Trend lnOD
lnDW Ss
Trend lnDW
Linear (Trend lnOD)
Linear (Trend lnDW)

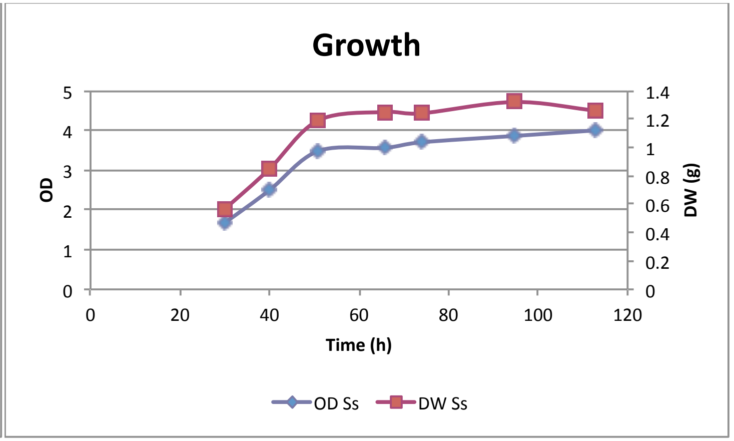
Growth
OD
DW (g)
Time (h)
OD Ss
DW Ss

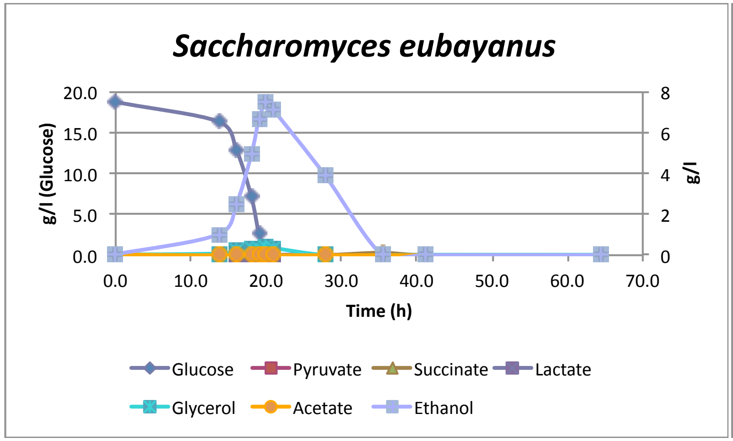
Saccharomyces eubayanus
g/l (Glucose)
g/l
Time (h)
Glucose
Pyruvate
Succinate
Lactate
Glycerol
Acetate
Ethanol

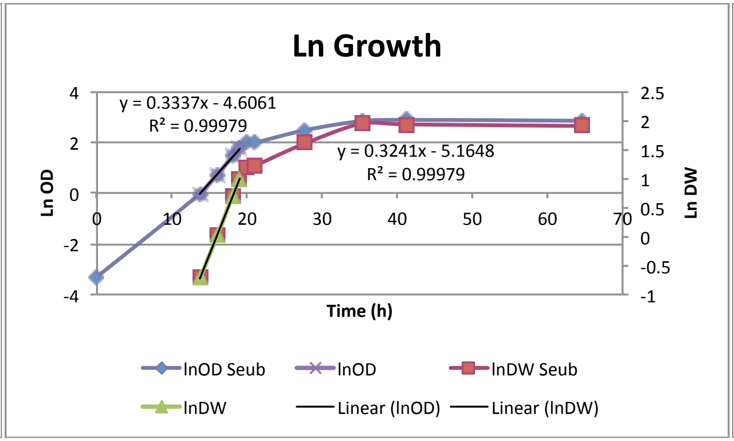
Ln Growth
y = 0.3337x - 4.6061
R² = 0.99979
y = 0.3241x - 5.1648
R² = 0.99979
Ln OD
Ln DW
Time (h)
lnOD Seub
lnOD
lnDW Seub
lnDW
Linear (lnOD)
Linear (lnDW)

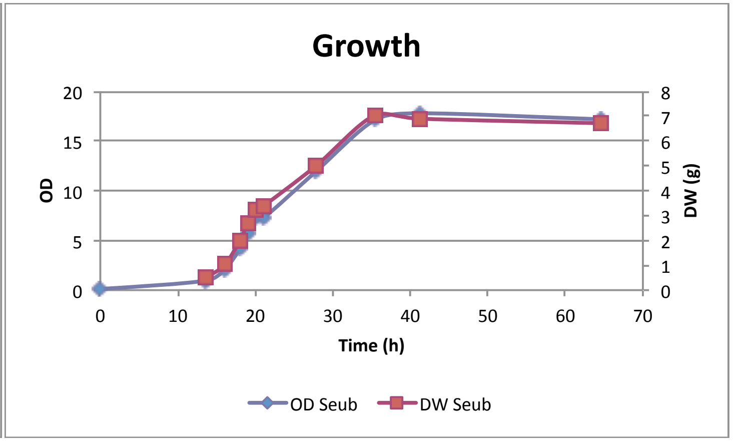
Growth
OD
DW (g)
Time (h)
OD Seub
DW Seub

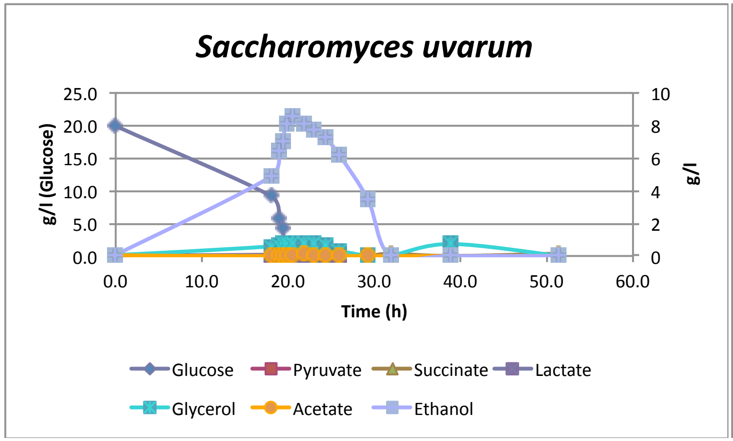
Saccharomyces uvarum
g/l (Glucose)
g/l
Time (h)
Glucose
Pyruvate
Succinate
Lactate
Glycerol
Acetate
Ethanol

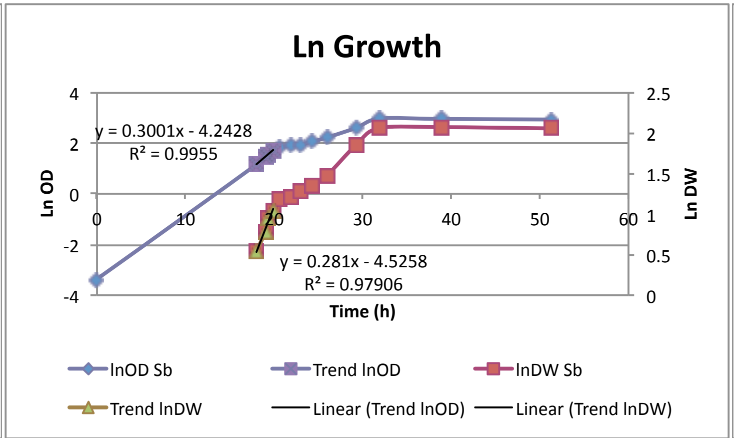
Ln Growth
y = 0.3001x - 4.2428
R² = 0.9955
y = 0.281x - 4.5258
R² = 0.97906
Ln OD
Ln DW
Time (h)
lnOD Sb
Trend lnOD
lnDW Sb
Trend lnDW
Linear (Trend lnOD)
Linear (Trend lnDW)

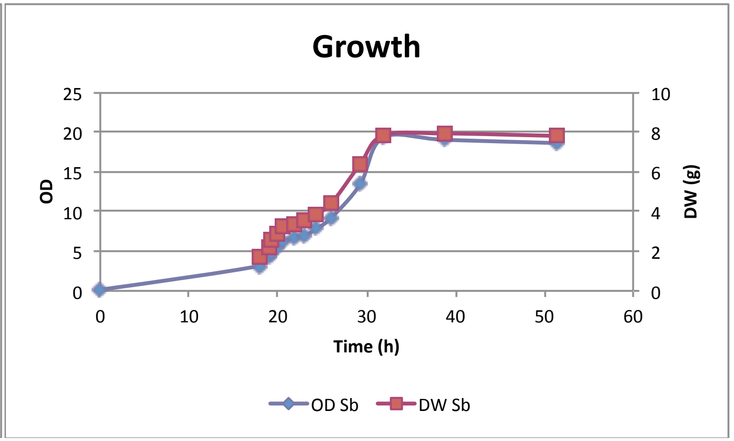
Growth
OD
DW (g)
Time (h)
OD Sb
DW Sb

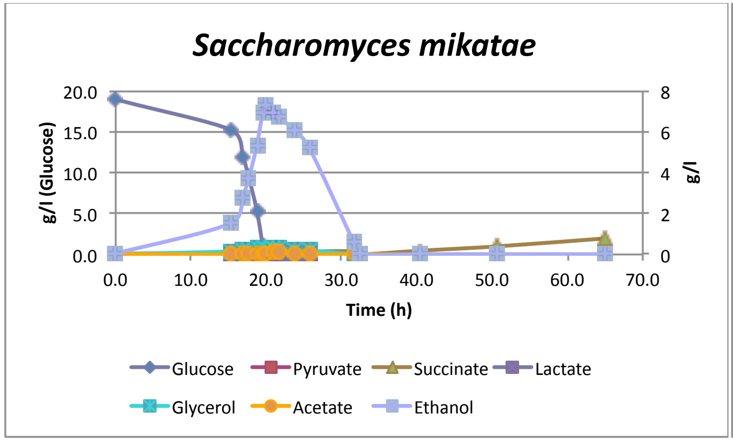
Saccharomyces mikatae
g/l (Glucose)
g/l
Time (h)
Glucose
Pyruvate
Succinate
Lactate
Glycerol
Acetate
Ethanol

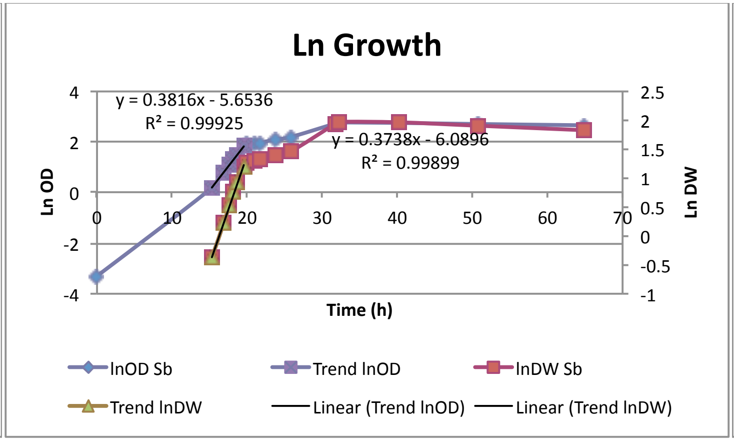
Ln Growth
y = 0.3816x - 5.6536
R² = 0.99925
y = 0.3738x - 6.0896
R² = 0.99899
Ln OD
Ln DW
Time (h)
lnOD Sb
Trend lnOD
lnDW Sb
Trend lnDW
Linear (Trend lnOD)
Linear (Trend lnDW)

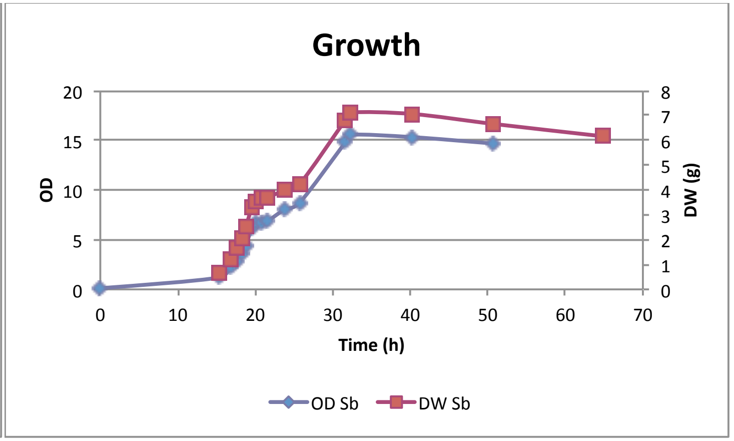
Growth
OD
DW (g)
Time (h)
OD Sb
DW Sb

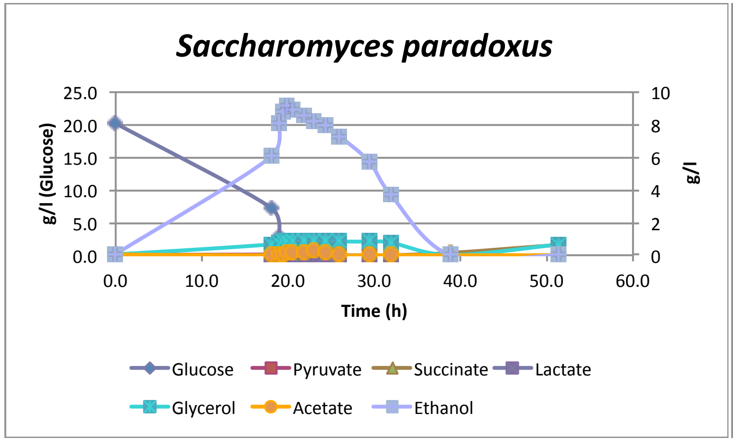
Saccharomyces paradoxus
g/l (Glucose)
g/l
Time (h)
Glucose
Pyruvate
Succinate
Lactate
Glycerol
Acetate
Ethanol

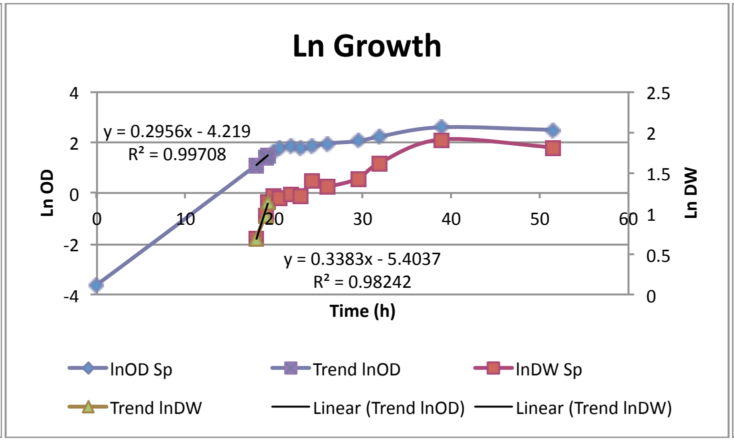
Ln Growth
y = 0.2956x - 4.219
R² = 0.99708
y = 0.3383x - 5.4037
R² = 0.98242
Ln OD
Ln DW
Time (h)
lnOD Sp
Trend lnOD
lnDW Sp
Trend lnDW
Linear (Trend lnOD)
Linear (Trend lnDW)

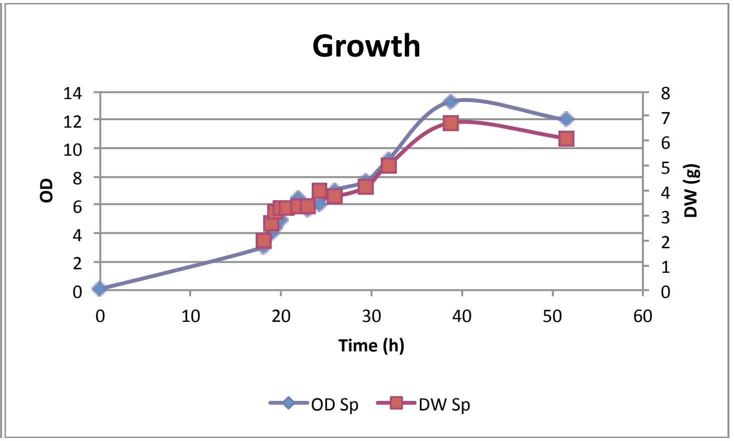
Growth
OD
DW (g)
Time (h)
OD Sp
DW Sp

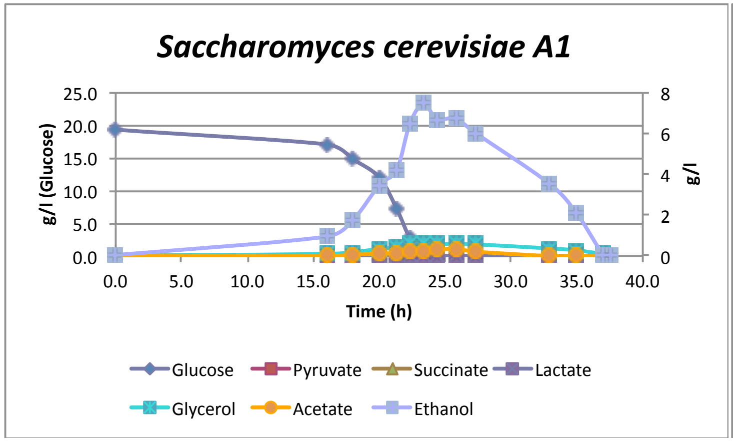
Saccharomyces cerevisiae A1
g/l (Glucose)
g/l
Time (h)
Glucose
Pyruvate
Succinate
Lactate
Glycerol
Acetate
Ethanol

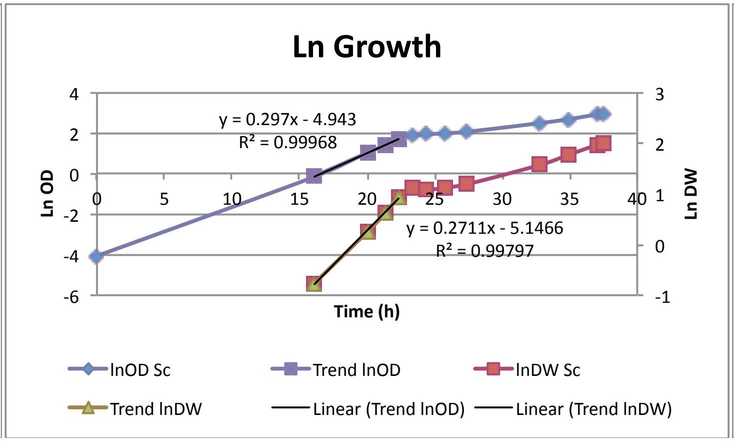
Ln Growth
y = 0.297x - 4.943
R² = 0.99968
y = 0.2711x - 5.1466
R² = 0.99797
Ln OD
Ln DW
Time (h)
lnOD Sc
Trend lnOD
lnDW Sc
Trend lnDW
Linear (Trend lnOD)
Linear (Trend lnDW)

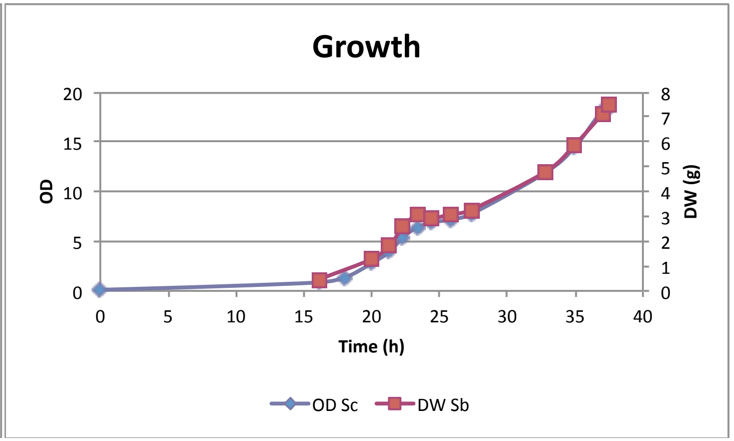
Growth
OD
DW (g)
Time (h)
OD Sc
DW Sb

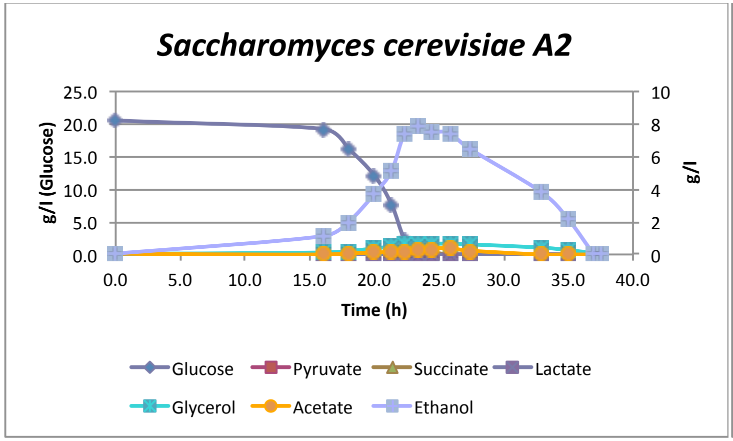
Saccharomyces cerevisiae A2
g/l (Glucose)
g/l
Time (h)
Glucose
Pyruvate
Succinate
Lactate
Glycerol
Acetate
Ethanol

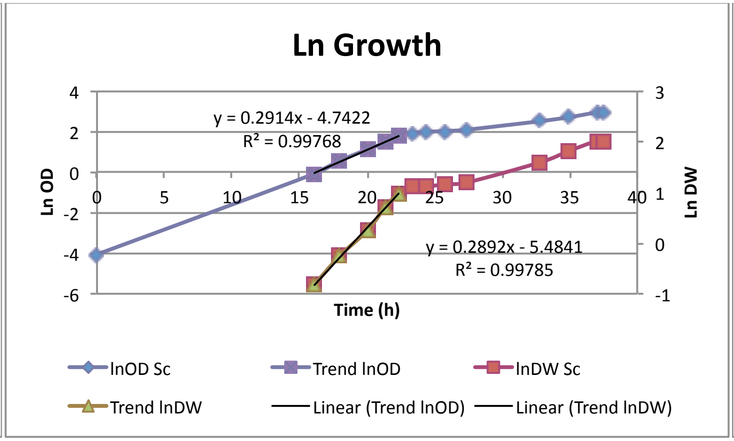
Ln Growth
Ln OD
Ln DW
Time (h)
y = 0.2914x - 4.7422
R² = 0.99768
y = 0.2892x - 5.4841
R² = 0.99785
lnOD Sc
Trend lnOD
lnDW Sc
Trend lnDW
Linear (Trend lnOD)
Linear (Trend lnDW)

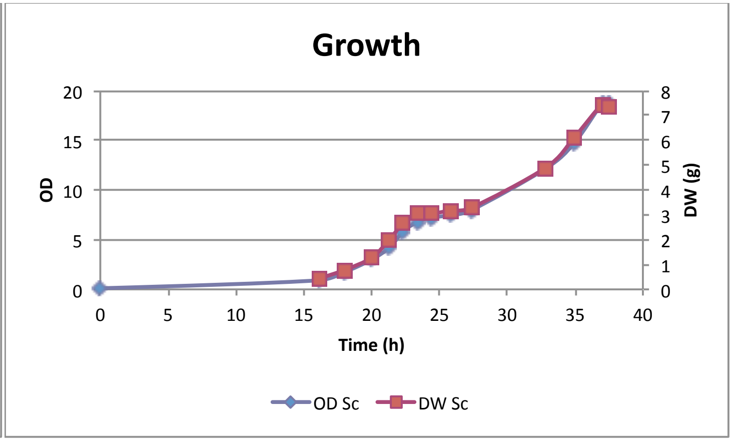
Growth
OD
DW (g)
Time (h)
OD Sc
DW Sc

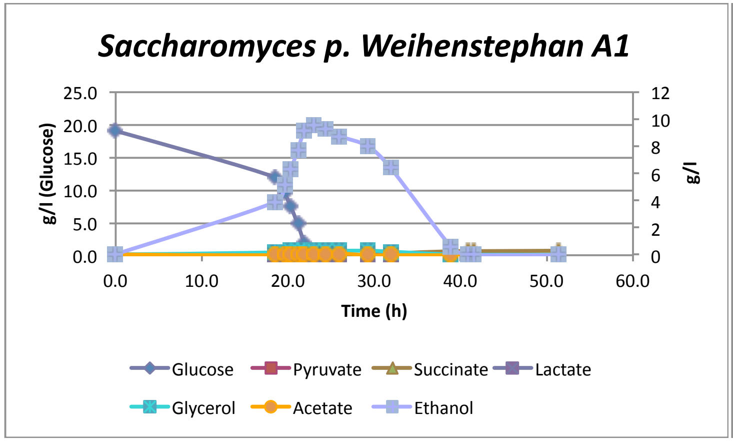
Saccharomyces p. Weihenstephan A1
g/l (Glucose)
g/l
Time (h)
Glucose
Pyruvate
Succinate
Lactate
Glycerol
Acetate
Ethanol

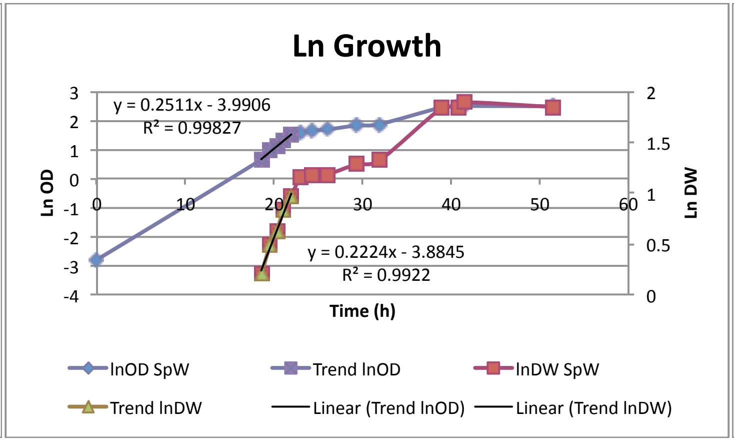
Ln Growth
Ln OD
Ln DW
Time (h)
y = 0.2511x - 3.9906
R² = 0.99827
y = 0.2224x - 3.8845
R² = 0.9922
lnOD SpW
Trend lnOD
lnDW SpW
Trend lnDW
Linear (Trend lnOD)
Linear (Trend lnDW)

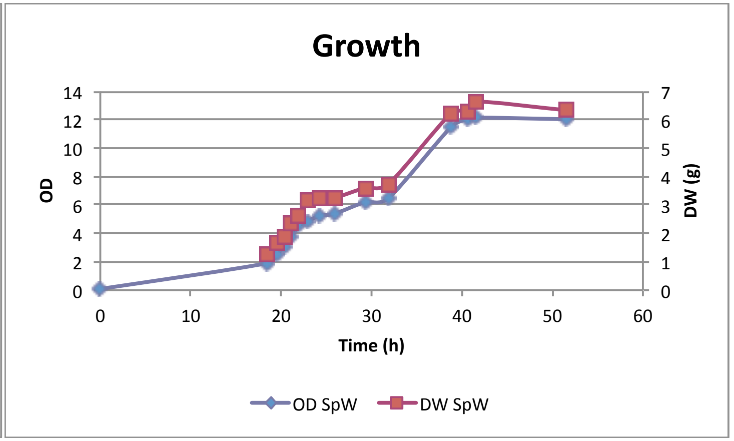
Growth
OD
DW (g)
Time (h)
OD SpW
DW SpW

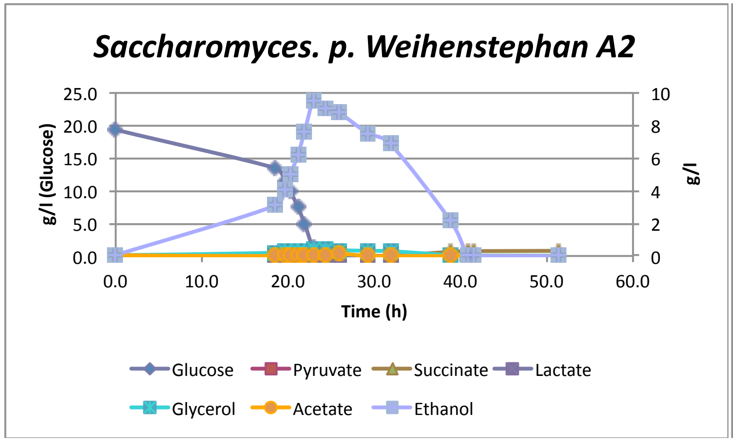
Saccharomyces. p. Weihenstephan A2
g/l (Glucose)
g/l
Time (h)
Glucose
Pyruvate
Succinate
Lactate
Glycerol
Acetate
Ethanol

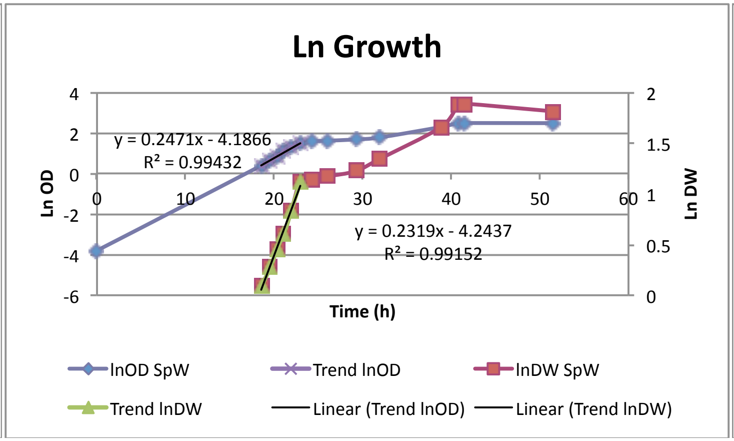
Ln Growth
Ln OD
Ln DW
Time (h)
y = 0.2471x - 4.1866
R² = 0.99432
y = 0.2319x - 4.2437
R² = 0.99152
lnOD SpW
Trend lnOD
lnDW SpW
Trend lnDW
Linear (Trend lnOD)
Linear (Trend lnDW)

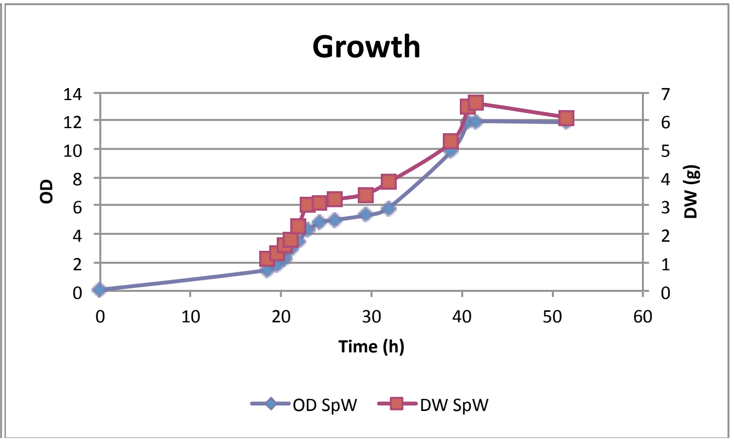
Growth
OD
DW (g)
Time (h)
OD SpW
DW SpW
