# Supplementary figures and images for: Yeast “Make-Accumulate-Consume” Life Strategy Evolved as a Multi-Step Process That Predates the Whole Genome Duplication
Source: PLoS One. 2013 Jul 15;8(7):e68734. doi: 10.1371/journal.pone.0068734 (PMC3711898; doi:10.1371/journal.pone.0068734)

# Respiration ratio

Species

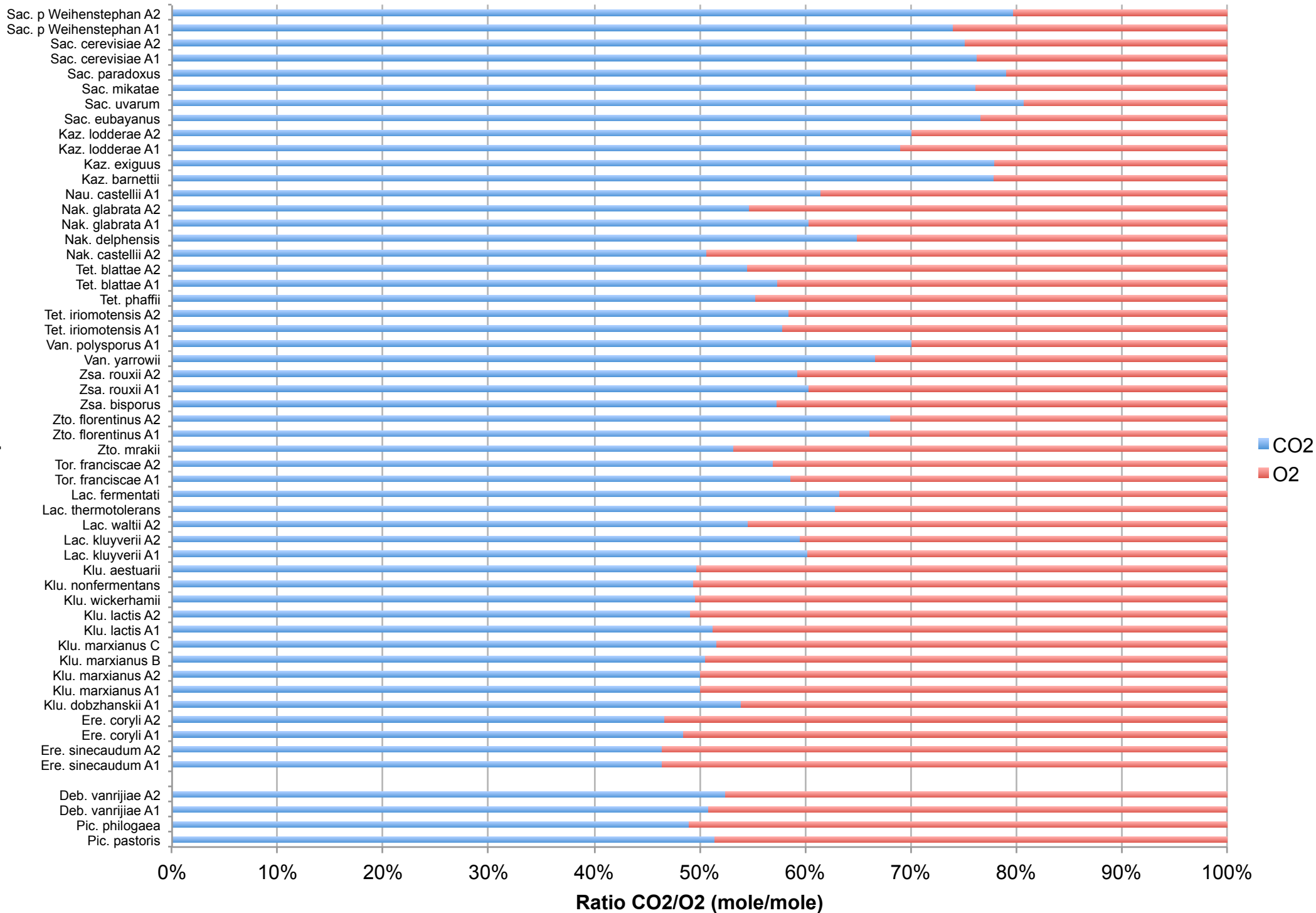

Supplement: Figure S2 — Respiration ratio. The respiration ratios for different species illustrates the activity of alcohol fermentation pathway as compared to respiratory pathway and was calculated by dividing the total amount of CO2 produced (blue bar) with total amount O2 consumed (red bar) in the unit mole/mole (see also table S1). The yeast species are presented starting with the Saccharomyces genus at the top and then following a decreasing phylogenetic relationship, following figure 1. The species related the least to Saccharomyces cerevisiae are at the bottom, and the gap divides the Saccharomycotina and non-Saccharomycotina yeasts. In general, the respiration ratio gradually drops with the phylogenetic distance from the Saccharomyces yeasts. (PDF) [file pone.0068734.s002.pdf]

**Biomass yield vs. Glucose cons. rates**

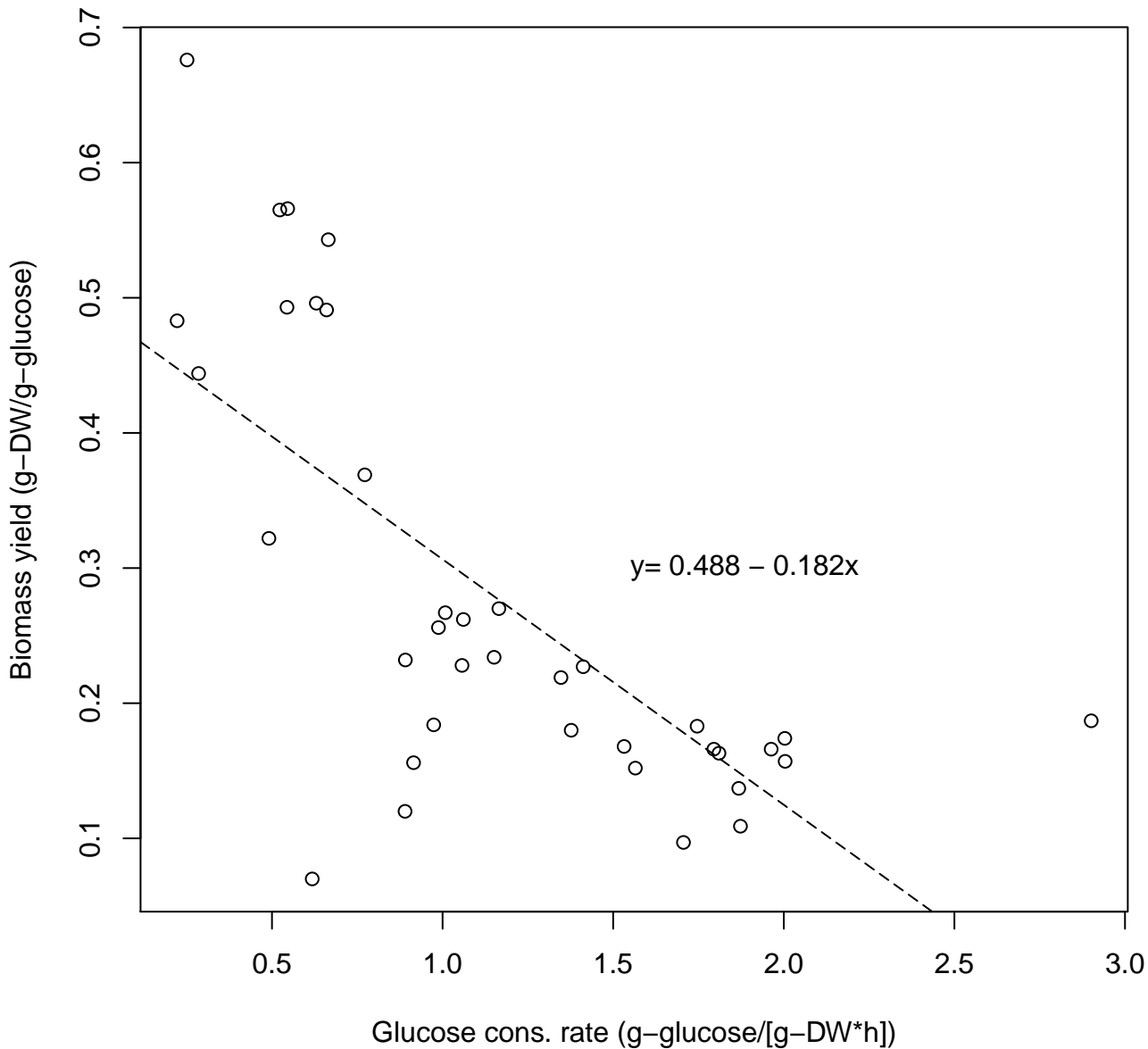

Supplement: Figure S4 — Correlation between biomass yield and glucose consumption rate. A significant correlation can be observed between the determined biomass yield and glucose consumption rate for each species (table 1). The rate-yield trade-off is a known phenomenon, which has been observed previously and hypothesized to act as an evolutionary constraint [31]. (PDF) [file pone.0068734.s004.pdf]
