# Supplementary material for: Yeast “Make-Accumulate-Consume” Life Strategy Evolved as a Multi-Step Process That Predates the Whole Genome Duplication
Source: PLoS One. 2013 Jul 15;8(7):e68734. doi: 10.1371/journal.pone.0068734 (PMC3711898; doi:10.1371/journal.pone.0068734)
Supplement: Figure S3 — Carbon balance. To verify the quality of each experiment, carbon balance was calculated by taking the yield ratio between measured products in C-mole and consumed substrate in C-mole, see also tables 1 and S1 for data on products yield and substrate consumption. (PDF) [file pone.0068734.s003.pdf]

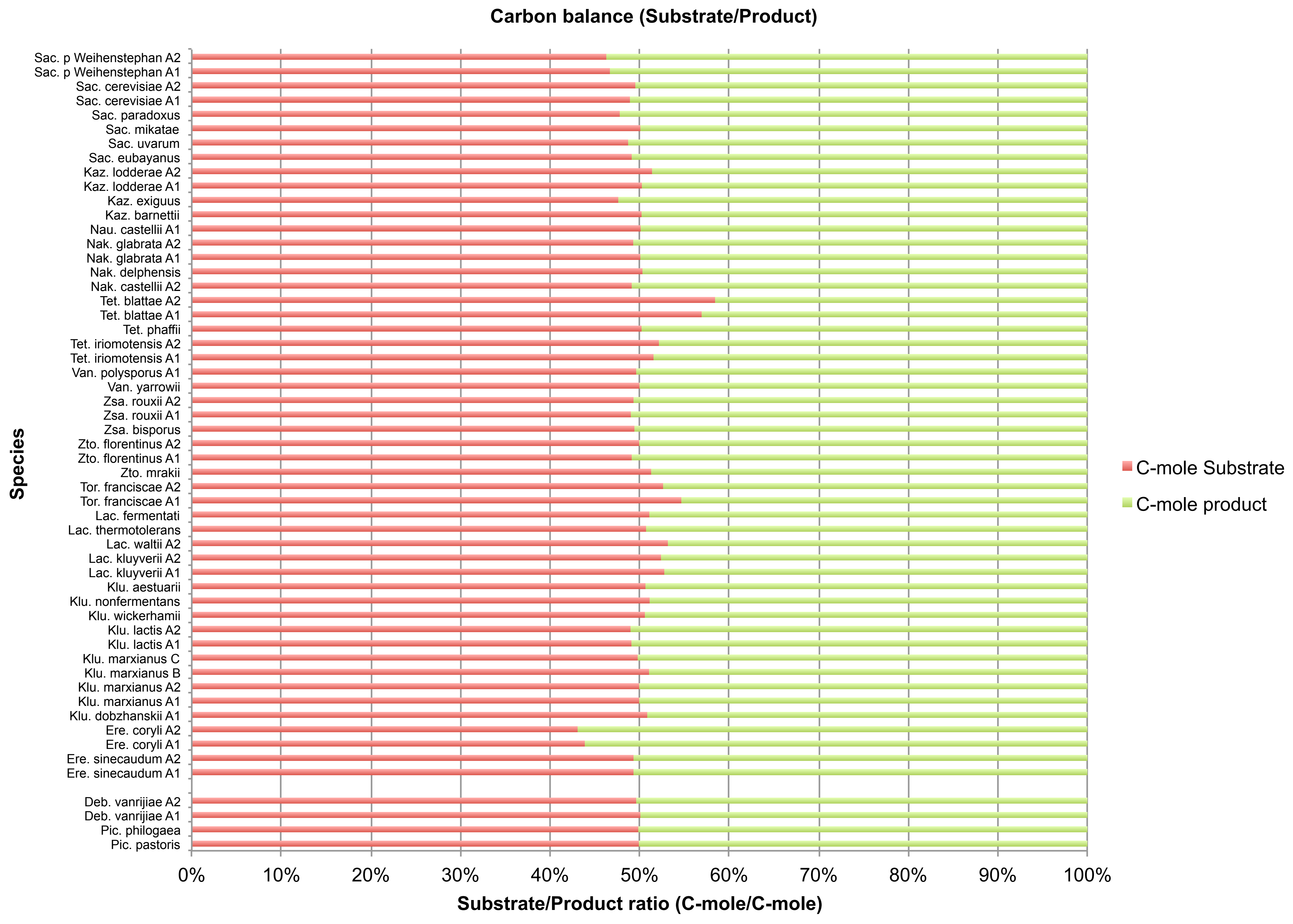
Carbon balance (Substrate/Product)
Sac. p Weihenstephan A2
Sac. p Weihenstephan A1
Sac. cerevisiae A2
Sac. cerevisiae A1
Sac. paradoxus
Sac. mikatae
Sac. uvarum
Sac. eubayanus
Kaz. lodderae A2
Kaz. lodderae A1
Kaz. exiguus
Kaz. barnettii
Nau. castellii A1
Nak. glabrata A2
Nak. glabrata A1
Nak. delphensis
Nak. castellii A2
Tet. blattae A2
Tet. blattae A1
Tet. phaffii
Tet. iriomotensis A2
Tet. iriomotensis A1
Van. polysporus A1
Van. yarrowii
Zsa. rouxii A2
Zsa. rouxii A1
Zsa. bisporus
Zto. florentinus A2
Zto. florentinus A1
Zto. mrakii
Tor. franciscae A2
Tor. franciscae A1
Lac. fermentati
Lac. thermotolerans
Lac. waltii A2
Lac. kluyverii A2
Lac. kluyverii A1
Klu. aestuarii
Klu. nonfermentans
Klu. wickerhamii
Klu. lactis A2
Klu. lactis A1
Klu. marxianus C
Klu. marxianus B
Klu. marxianus A2
Klu. marxianus A1
Klu. dobzhanskii A1
Ere. coryli A2
Ere. coryli A1
Ere. sinecaudum A2
Ere. sinecaudum A1
Deb. vanrijiae A2
Deb. vanrijiae A1
Pic. philogaea
Pic. pastoris
Species
0%
10%
20%
30%
40%
50%
60%
70%
80%
90%
100%
Substrate/Product ratio (C-mole/C-mole)
C-mole Substrate
C-mole product
